# Supplementary material for: Dissecting the chain of information processing and its interplay with neurochemicals and fluid intelligence across development
Source: eLife. 2023 Sep 29;12:e84086. doi: 10.7554/eLife.84086 (PMC10541179; doi:10.7554/eLife.84086)
Supplement: Supplementary file 1. [file elife-84086-supp1.docx]

**Supplementary File 1.** Gender and mean age (standard deviation in parentheses) during the first (A1, top half) and the second (A2, bottom half) assessment.

| **Group** | **Females** | **Males** | **Age** |
| --- | --- | --- | --- |
| First Assessment (A1) | | | |
| Early Childhood | 28 | 23 | 6.5 (.29) |
| Late Childhood | 27 | 24 | 10.45 (.32) |
| Early Adolescence | 25 | 25 | 14.4 (.33) |
| Late Adolescence | 56 | 31 | 16.89 (.39) |
| Early Adulthood | 21 | 33 | 18.89 (.62) |
| Second Assessment (A2) | | | |
| Early Childhood | 22 | 21 | 8.19 (.37) |
| Late Childhood | 22 | 18 | 12.29 (.49) |
| Early Adolescence | 16 | 19 | 16.13 (.40) |
| Late Adolescence | 26 | 17 | 18.45 (.54) |
| Early Adulthood | 12 | 22 | 20.60 (.65) |
