# Supplementary material for: Dissecting the chain of information processing and its interplay with neurochemicals and fluid intelligence across development
Source: eLife. 2023 Sep 29;12:e84086. doi: 10.7554/eLife.84086 (PMC10541179; doi:10.7554/eLife.84086)

**Supplementary File 2.** Scatterplots depicting the association between chronological age (x-axis) and the diffusion parameters (y-axis) either during the first assessment (A1) or during the second assessment (A2) with a linear (yellow), quadratic (purple) and a cubic (green) fit, in the Attention Network Task (ANT), the Digit Comparison Task (DC), and the Mental Rotation Task (MRT).

**Supplementary File 2.1.** Mean Drift Rate calculated across all the trials.

**
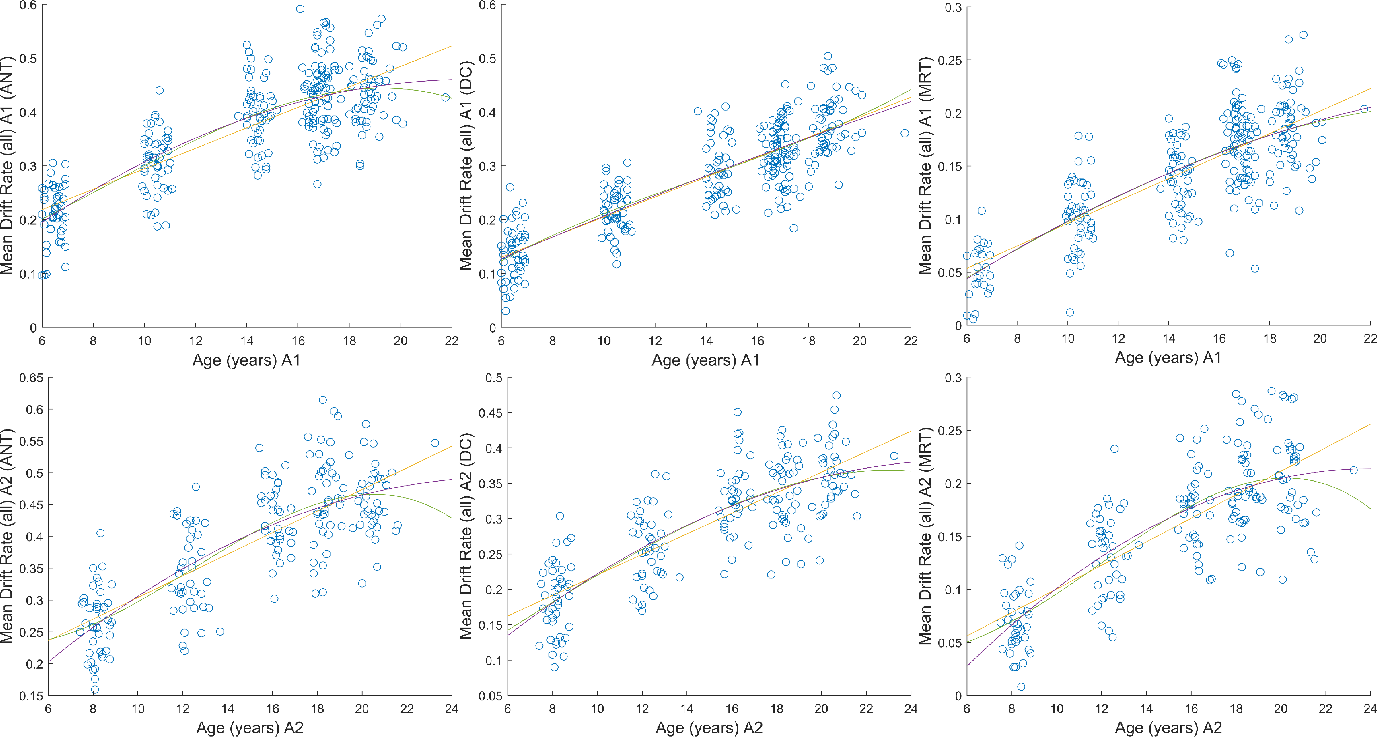
**

**Supplementary File 2.2.** Boundary separation calculated across all the trials.

**
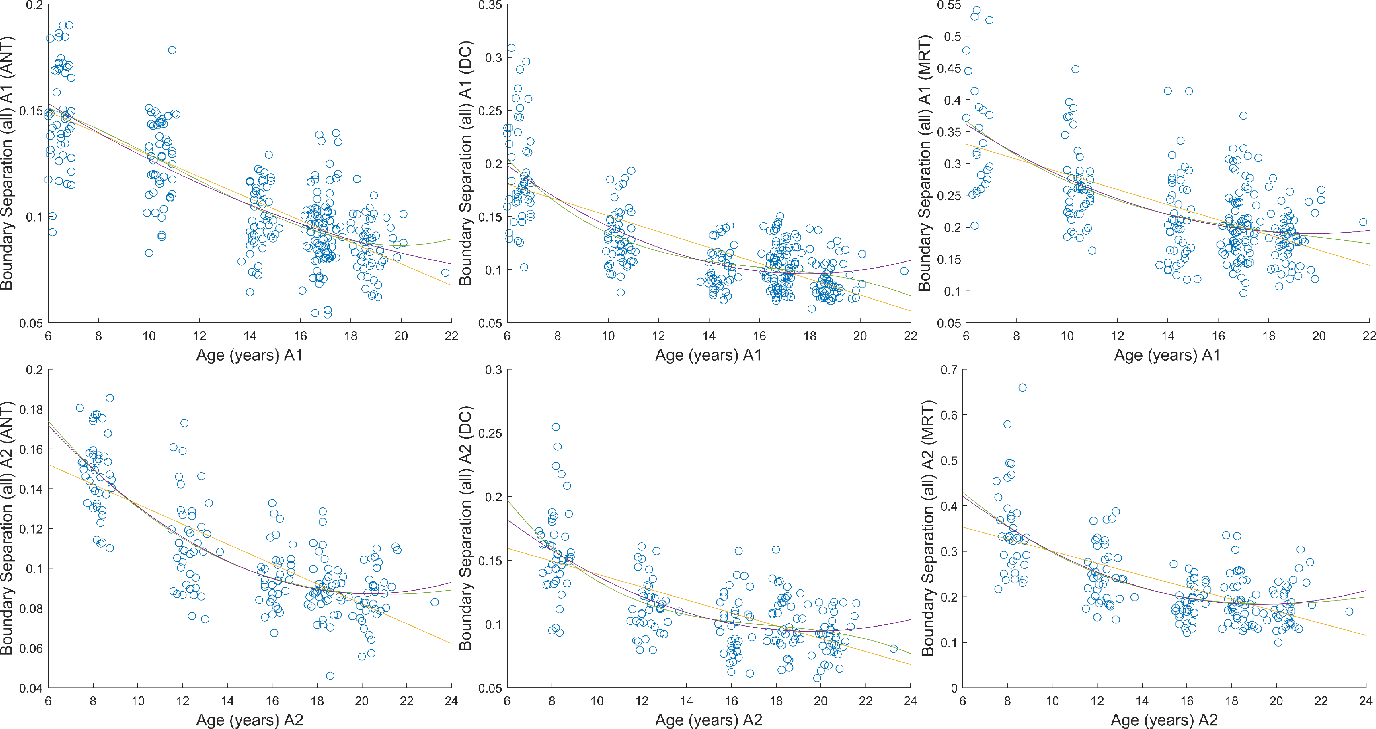
**

**Supplementary File 2.3.** Non-decision time calculated across all the trials.


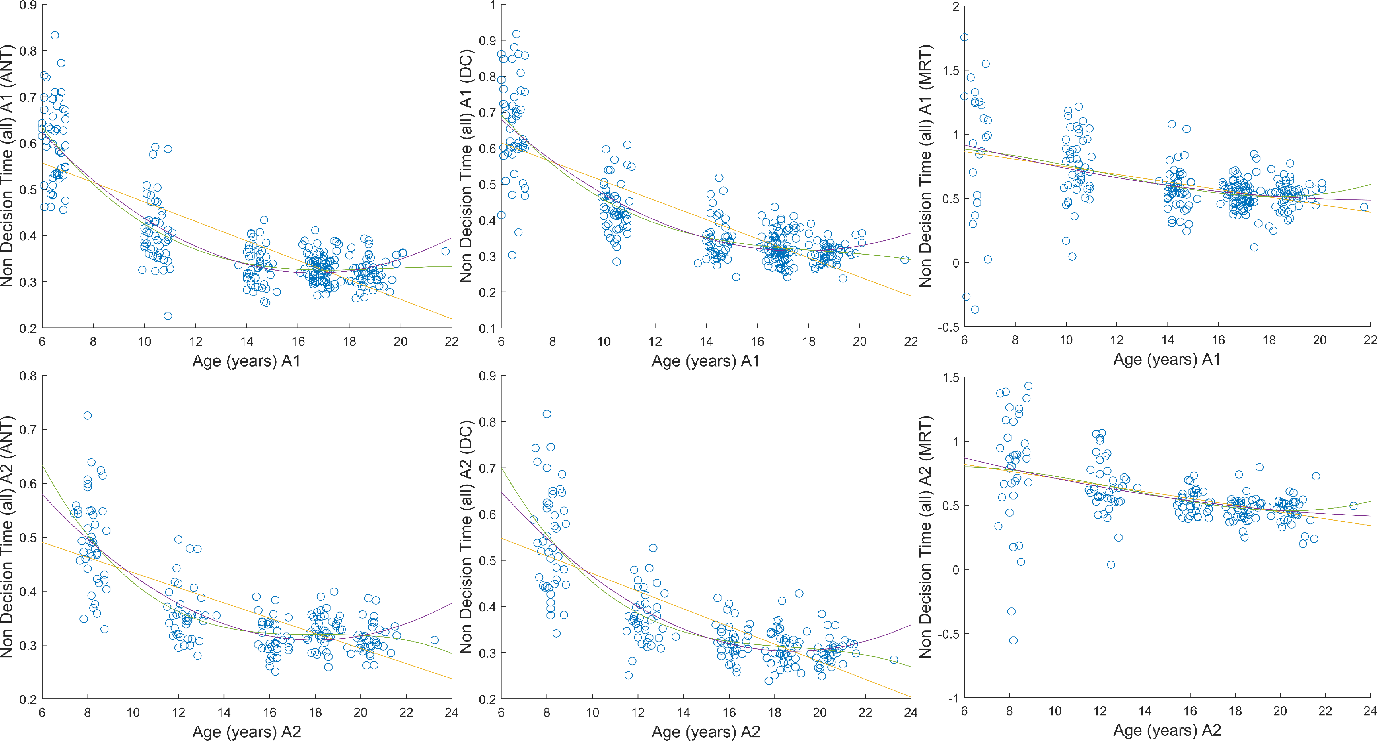


**Supplementary File 2.4.** The three parameters of the alerting network (**Task 1**). The alerting network was calculated by subtracting the diffusion parameter during the double-cue condition from the same diffusion parameter in the no-cue condition.


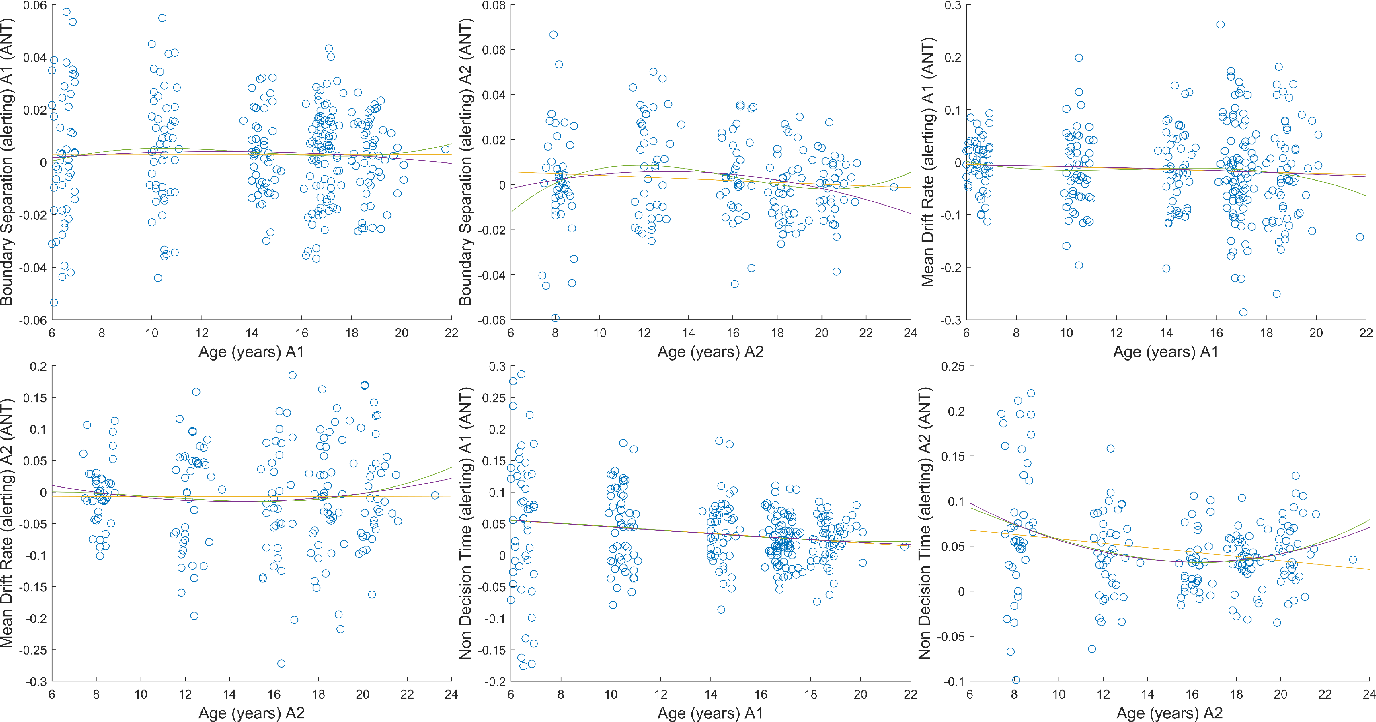


**Supplementary File 2.5.** The three parameters of the orienting network (**Task 1**). The orienting network was calculated by subtracting the diffusion parameter during the spatial cue condition from the same diffusion parameter in the central cue condition.


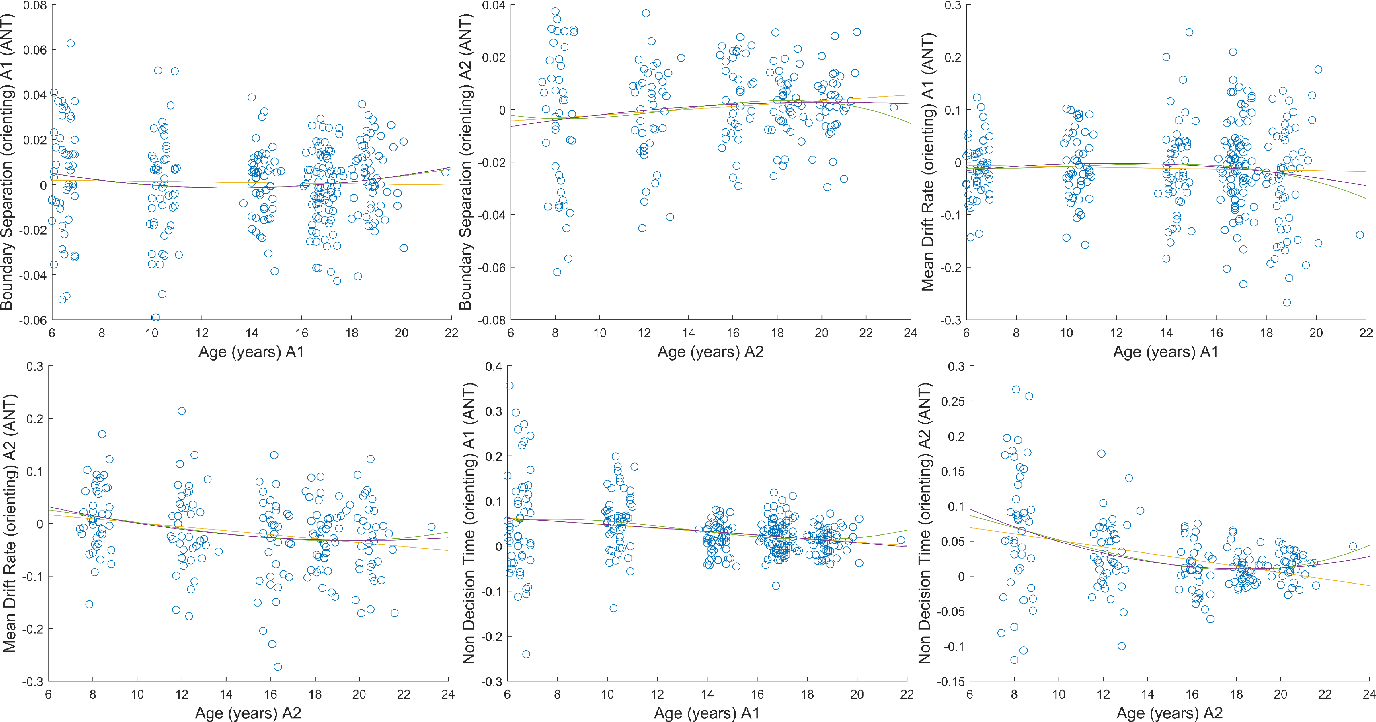


**Supplementary File 2.6.** The three parameters of the executive network (**Task 1**). The executive network was calculated by subtracting the diffusion parameter during the congruent condition from the same diffusion parameter in the incongruent condition.


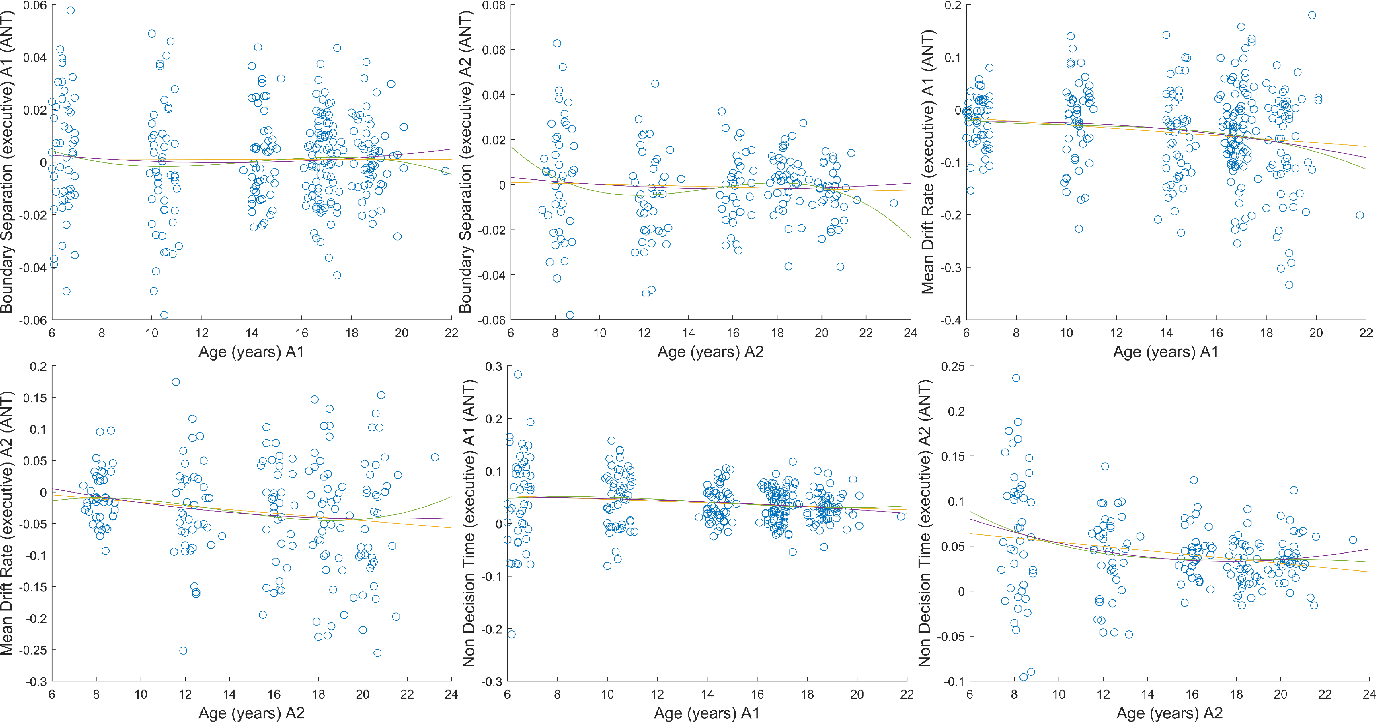


**Supplementary File 2.7.** The three diffusion parameters of the distance effect obtained from contrasting “far” trials vs. “near” trials (**Task 2**).


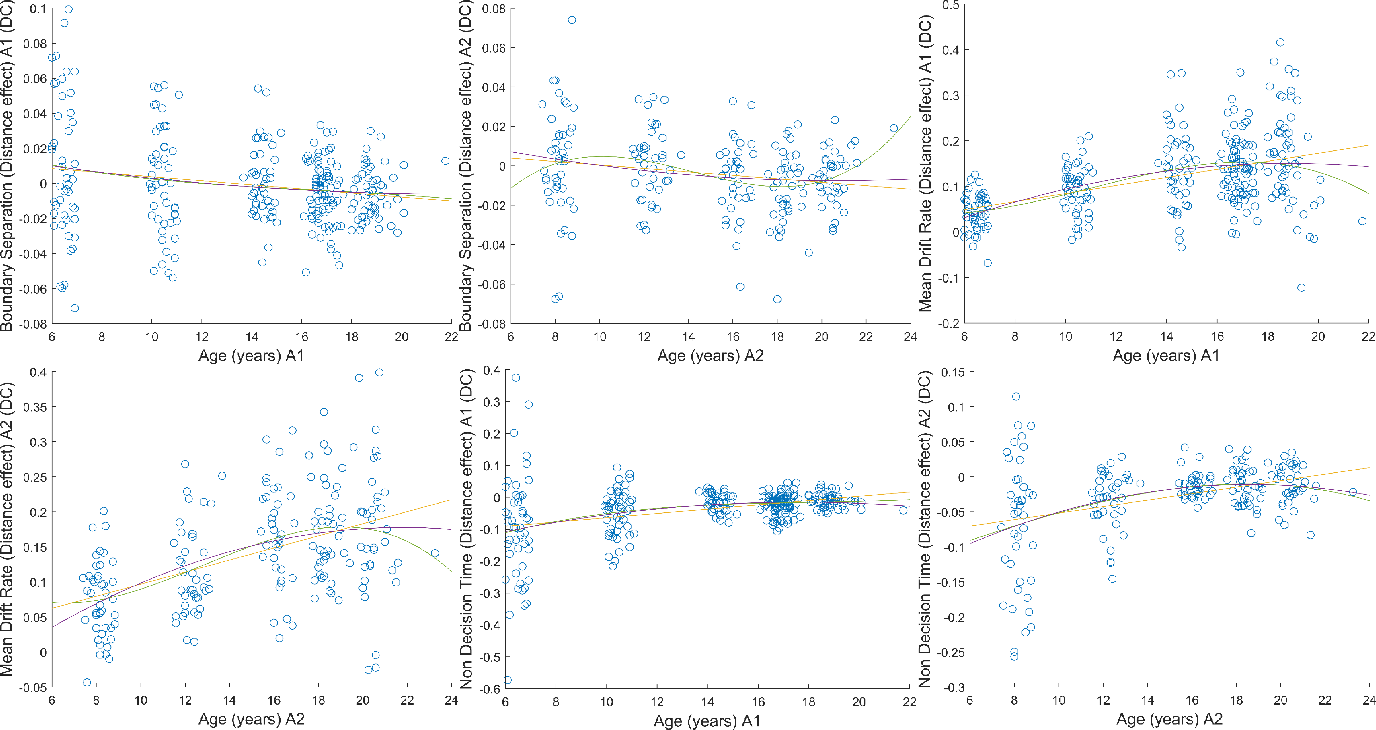


**Supplementary File 2.8.** The three diffusion parameters of the distance effect obtained from contrasting 135 degrees trials vs. 45 degrees trials (**Task 3**).


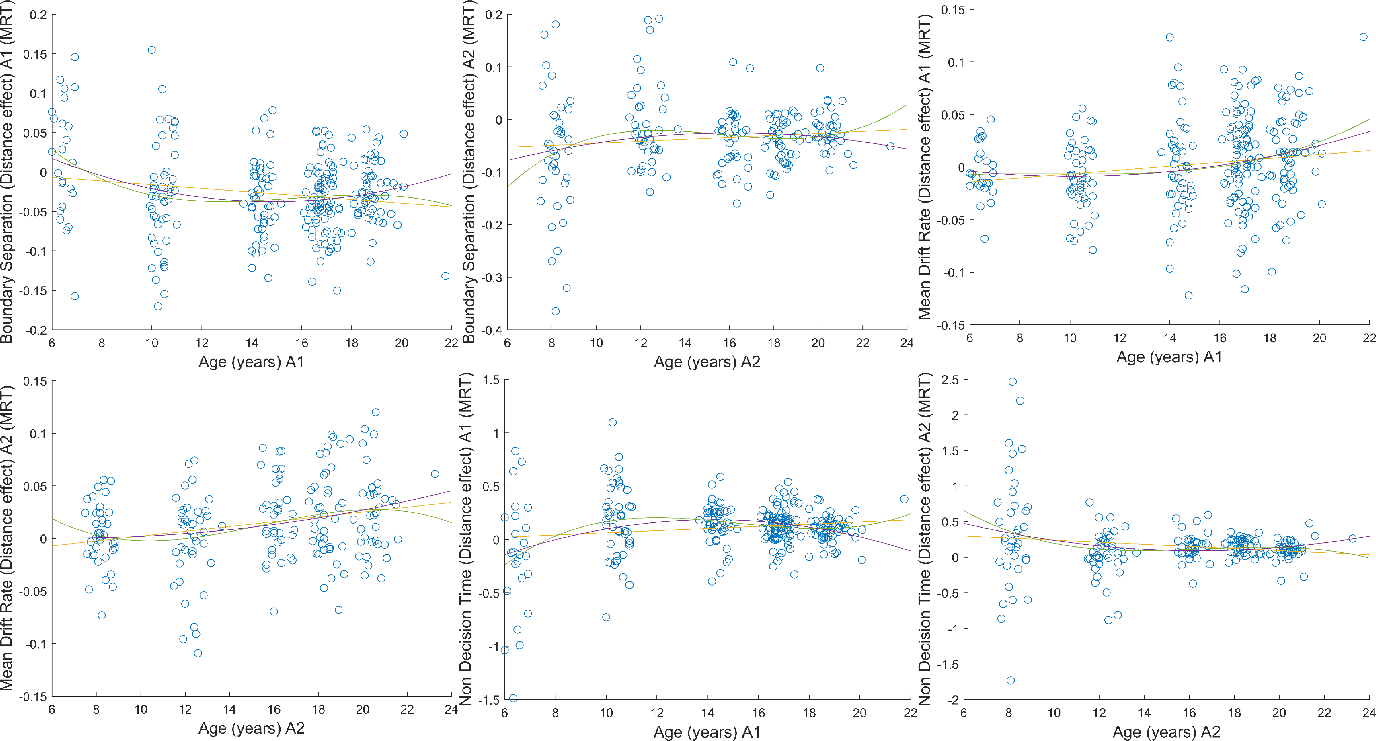


**Supplementary File 2.9.** The three diffusion parameters of the SNARC effect obtained from contrasting low SNARC trials vs high SNARC trials (**Task 2**).


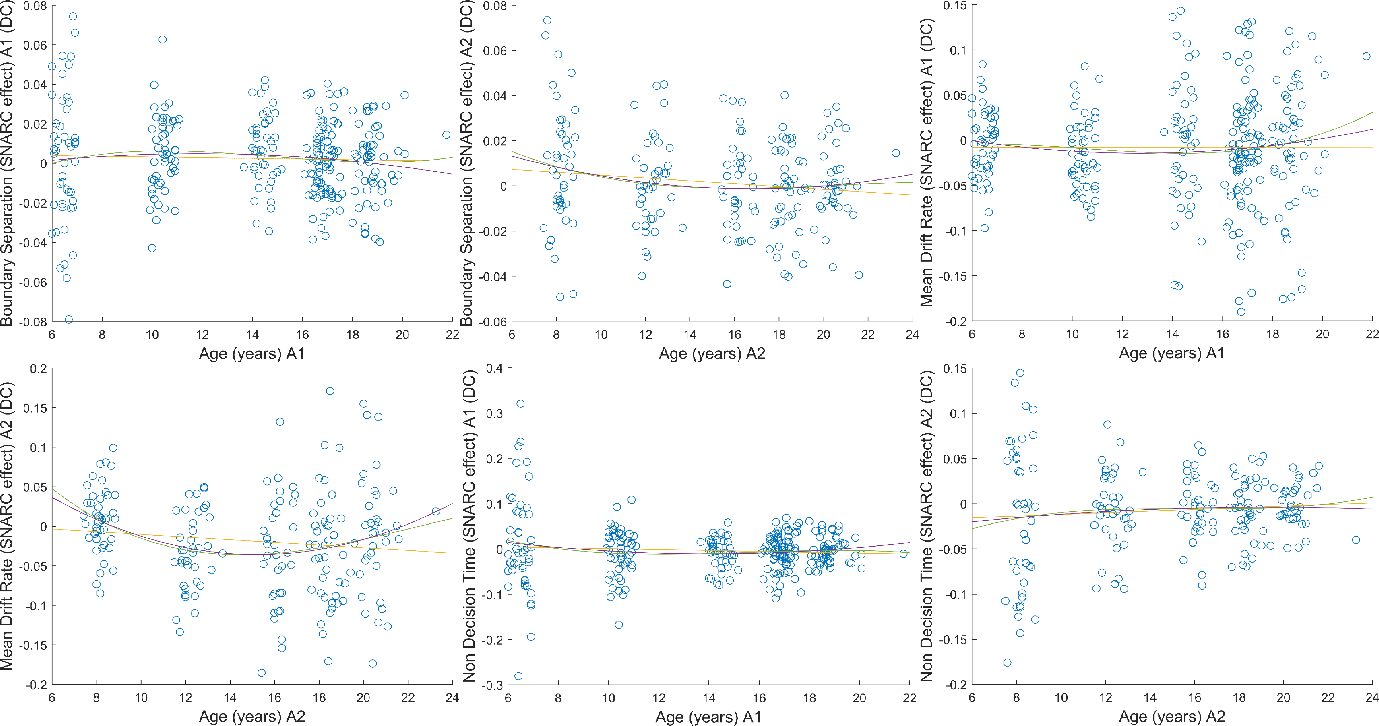

Supplement: Supplementary file 2. — 1. Mean Drift Rate calculated across all the trials. 2. Boundary separation calculated across all the trials. 3. Non-decision time calculated across all the trials. 4. The three parameters of the alerting network (Task 1). The alerting network was calculated by subtracting the diffusion parameter during the double-cue condition from the same diffusion parameter in the no-cue condition. 5. The three parameters of the orienting network (Task 1). The orienting network was calculated by subtracting the diffusion parameter during the spatial cue condition from the same diffusion parameter in the central cue condition. 6. The three parameters of the executive network (Task 1). The executive network was calculated by subtracting the diffusion parameter during the congruent condition from the same diffusion parameter in the incongruent condition. 7. The three diffusion parameters of the distance effect obtained from contrasting “far” trials vs. “near” trials (Task 2). 8. The three diffusion parameters of the distance effect obtained from contrasting 135 degrees trials vs. 45 degrees trials (Task 3). 9. The three diffusion parameters of the SNARC effect obtained from contrasting low SNARC trials vs high SNARC trials (Task 2). [file elife-84086-supp2.docx]
