# Supplementary material for: Dissecting the chain of information processing and its interplay with neurochemicals and fluid intelligence across development
Source: eLife. 2023 Sep 29;12:e84086. doi: 10.7554/eLife.84086 (PMC10541179; doi:10.7554/eLife.84086)
Supplement: Supplementary file 3. [file elife-84086-supp3.docx]

**Supplementary File 3**. Multiple linear regressions with bootstrapping predicting overall visuomotor processing (A1: first assessment, A2: second assessment, β=the regression coefficient of the variable listed in the “Effect” column, df=degrees of freedom, T=t-statistic, P_B_=Bootstrapped P-value, CI_L=lower bound of the confidence intervals obtained from bootstrapping, CI_U=upper bound of the confidence intervals obtained from bootstrapping) for **Task 1** (Attention network task, top third), **Task 2** (Digit comparison task, middle third), and **Task 3** (Mental rotation task, bottom third).

| **Assessment** | **Task** | **Effect** | **df** | **β** | **T** | **CI_L** | **CI_U** | **P_B_** |
| --- | --- | --- | --- | --- | --- | --- | --- | --- |
| A1 | Task 1 | IPS Glutamate*age | 252 | -0.22 | -5.38 | -0.31 | -0.11 | 0.00004 |
| A1 | Task 1 | IPS GABA*age | 252 | 0.23 | 5.71 | 0.14 | 0.32 | <.0001 |
| A2 | Task 1 | IPS Glutamate*age | 174 | -0.24 | -4.47 | -0.35 | -0.11 | 0.00014 |
| A2 | Task 1 | IPS GABA*age | 175 | 0.24 | 4.41 | 0.11 | 0.41 | 0.00168 |
| A1 | Task 2 | IPS Glutamate*age | 240 | -0.17 | -4.25 | -0.26 | -0.09 | 0.00006 |
| A1 | Task 2 | IPS GABA*age | 240 | 0.18 | 5.1 | 0.08 | 0.27 | 0.00019 |
| A2 | Task 2 | IPS Glutamate*age | 170 | -0.16 | -3.17 | -0.28 | -0.02 | 0.01661 |
| A2 | Task 2 | IPS GABA*age | 168 | 0.29 | 6.02 | 0.18 | 0.4 | <.0001 |
| A1 | Task 3 | IPS Glutamate*age | 222 | -0.27 | -5.02 | -0.42 | -0.11 | 0.00055 |
| A1 | Task 3 | IPS GABA*age | 224 | 0.27 | 4.66 | 0.11 | 0.43 | 0.00111 |
| A2 | Task 3 | IPS Glutamate*age | 164 | 0.11 | 1.67 | -0.05 | 0.3 | 0.2063 |
| A2 | Task 3 | IPS GABA*age | 166 | 0.39 | 5.46 | 0.22 | 0.56 | 0.00001 |
