# Supplementary material for: Dissecting the chain of information processing and its interplay with neurochemicals and fluid intelligence across development
Source: eLife. 2023 Sep 29;12:e84086. doi: 10.7554/eLife.84086 (PMC10541179; doi:10.7554/eLife.84086)
Supplement: Supplementary file 4. — Regarding the column “Ord”, here we run three models (i) M1 which featured the intercept and the linear fit, (ii) M2 which featured the intercept, the linear fit and the quadratic fit and (iii) M3 which featured the intercept, the linear fit, the quadratic fit and the cubic fit. Following that, we assigned three p-values, (i) the p-value of the linear fit from M1, (ii) the p-value of the quadratic fit from M2, and (iii) the p-value of the cubic fit from M3. If none of these p-values was less than .05, the “Ord” value is N/A. If only the p-value of the linear fit from M1 is significant then “Ord” is 1, if the p-value of the quadratic fit from M2 is significant but the p-value of the cubic fit from M3 is not significant then “Ord” is 2, and if the p-value of the cubic fit from M3 is significant then “Ord” is 3. Essentially, the “Ord” column indicates the highest order fit that significantly contributes to the data above and beyond the less higher order fit/s. The variables (both for the first and the second assessment) are sorted based on the Pearson’s p-value of the first assessment. [file elife-84086-supp4.docx]

**Supplementary File 4.** Additional data regarding linear, quadratic, and cubic fits between diffusion parameters and chronological age (Task 1=Attention Network Task, Task 2=Digit Comparison Task, Task 3=Mental Rotation Task, v= mean drift rate, a=boundary separation, Ter=non-decision time, DF= degrees of freedom, rP=Pearson r, pP=p-value of rP, Spearman’s rho=rS, pS=p-value of rS, l.aR^2^=adjusted R^2^ of the model featuring the intercept and the linear fit, q.aR^2^=adjusted R^2^ of the model featuring the intercept, the linear fit, and the quadratic fit, c.aR^2^=adjusted R^2^ of the model featuring the intercept, the linear fit the quadratic and the cubit fit. Regarding the column “Ord”, here we run three models (i) M1 which featured the intercept and the linear fit, (ii) M2 which featured the intercept, the linear fit and the quadratic fit and (iii) M3 which featured the intercept, the linear fit, the quadratic fit and the cubic fit. Following that, we assigned three p-values, (i) the p-value of the linear fit from M1, (ii) the p-value of the quadratic fit from M2, and (iii) the p-value of the cubic fit from M3. If none of these p-values was less than .05, the “Ord” value is N/A. If only the p-value of the linear fit from M1 is significant then “Ord” is 1, if the p-value of the quadratic fit from M2 is significant but the p-value of the cubic fit from M3 is not significant then “Ord” is 2, and if the p-value of the cubic fit from M3 is significant then “Ord” is 3. Essentially, the “Ord” column indicates the highest order fit that significantly contributes to the data above and beyond the less higher order fit/s. The variables (both for the first and the second assessment) are sorted based on the Pearson’s p-value of the first assessment.

| First Assessment | | | | | | | | | |
| --- | --- | --- | --- | --- | --- | --- | --- | --- | --- |
|  | **DF** | **rP** | **pP** | **rS** | **pS** | **l.aR^2^** | **q.aR^2^** | **c.aR^2^** | **Ord.** |
| Task 2 v | 278 | 0.85 | 0.0000 | 0.81 | 0.0000 | 0.72 | 0.72 | 0.72 | 1 |
| Task 2 Ter | 277 | -0.81 | 0.0000 | -0.73 | 0.0000 | 0.66 | 0.74 | 0.74 | 3 |
| Task 1 v | 286 | 0.80 | 0.0000 | 0.73 | 0.0000 | 0.64 | 0.66 | 0.66 | 2 |
| Task 1 Ter | 284 | -0.80 | 0.0000 | -0.68 | 0.0000 | 0.65 | 0.77 | 0.78 | 3 |
| Task 1 a | 285 | -0.77 | 0.0000 | -0.70 | 0.0000 | 0.60 | 0.60 | 0.60 | 2 |
| Task 3 v | 252 | 0.77 | 0.0000 | 0.69 | 0.0000 | 0.59 | 0.59 | 0.59 | 1 |
| Task 2 a | 278 | -0.74 | 0.0000 | -0.66 | 0.0000 | 0.54 | 0.60 | 0.61 | 3 |
| Task 3 a | 252 | -0.59 | 0.0000 | -0.50 | 0.0000 | 0.35 | 0.38 | 0.38 | 2 |
| Task 2 v Distance effect | 277 | 0.46 | 0.0000 | 0.44 | 0.0000 | 0.21 | 0.22 | 0.23 | 2 |
| Task 3 Ter | 251 | -0.45 | 0.0000 | -0.38 | 0.0000 | 0.20 | 0.21 | 0.21 | 1 |
| Task 2 Ter Distance effect | 276 | 0.34 | 0.0000 | 0.30 | 0.0000 | 0.12 | 0.13 | 0.12 | 2 |
| Task 1 Ter orienting | 286 | -0.26 | 0.0000 | -0.21 | 0.0003 | 0.06 | 0.06 | 0.06 | 1 |
| Task 2 a Distance effect | 278 | -0.20 | 0.0009 | -0.15 | 0.0102 | 0.04 | 0.03 | 0.03 | 1 |
| Task 1 Ter alerting | 285 | -0.18 | 0.0026 | -0.18 | 0.0029 | 0.03 | 0.02 | 0.02 | 1 |
| Task 1 v executive | 286 | -0.17 | 0.0035 | -0.14 | 0.0176 | 0.03 | 0.03 | 0.02 | 1 |
| Task 3 v Distance effect | 252 | 0.17 | 0.0055 | 0.18 | 0.0032 | 0.03 | 0.03 | 0.03 | 1 |
| Task 3 a Distance effect | 251 | -0.17 | 0.0068 | -0.05 | 0.3957 | 0.02 | 0.06 | 0.06 | 2 |
| Task 1 Ter executive | 286 | -0.14 | 0.0149 | -0.14 | 0.0177 | 0.02 | 0.01 | 0.01 | 1 |
| Task 3 Ter Distance effect | 250 | 0.14 | 0.0238 | -0.03 | 0.6395 | 0.02 | 0.08 | 0.10 | 3 |
| Task 2 Ter SNARC effect | 276 | -0.08 | 0.2113 | -0.01 | 0.9092 | 0.00 | 0.01 | 0.01 | N/A |
| Task 1 v alerting | 284 | -0.07 | 0.2640 | -0.09 | 0.1447 | 0.00 | 0.00 | 0.00 | N/A |
| Task 1 v orienting | 286 | -0.04 | 0.4971 | -0.02 | 0.7548 | 0.00 | 0.00 | 0.00 | N/A |
| Task 2 a SNARC effect | 279 | -0.04 | 0.5499 | -0.04 | 0.4791 | 0.00 | 0.00 | 0.00 | N/A |
| Task 1 a orienting | 286 | -0.03 | 0.6017 | -0.01 | 0.9238 | 0.00 | 0.00 | 0.00 | N/A |
| Task 1 a executive | 286 | 0.00 | 0.9541 | 0.01 | 0.8855 | 0.00 | 0.00 | 0.00 | N/A |
| Task 2 v SNARC effect | 277 | 0.00 | 0.9877 | 0.05 | 0.4393 | 0.00 | 0.00 | 0.00 | N/A |
| Task 1 a alerting | 286 | 0.00 | 0.9944 | 0.02 | 0.7662 | 0.00 | -0.01 | -0.01 | N/A |
| Second Assessment | | | | | | | | | |
|  | **DF** | **rP** | **pP** | **rS** | **pS** | **l.aR^2^** | **q.aR^2^** | **c.aR^2^** | **Ord.** |
| Task 2 v | 190 | 0.79 | 0.0000 | 0.77 | 0.0000 | 0.63 | 0.64 | 0.64 | 2 |
| Task 2 Ter | 191 | -0.76 | 0.0000 | -0.74 | 0.0000 | 0.58 | 0.67 | 0.67 | 3 |
| Task 1 v | 193 | 0.77 | 0.0000 | 0.73 | 0.0000 | 0.60 | 0.61 | 0.61 | 2 |
| Task 1 Ter | 192 | -0.73 | 0.0000 | -0.65 | 0.0000 | 0.53 | 0.65 | 0.66 | 3 |
| Task 1 a | 193 | -0.77 | 0.0000 | -0.71 | 0.0000 | 0.59 | 0.64 | 0.64 | 2 |
| Task 3 v | 187 | 0.77 | 0.0000 | 0.73 | 0.0000 | 0.60 | 0.62 | 0.62 | 2 |
| Task 2 a | 190 | -0.66 | 0.0000 | -0.61 | 0.0000 | 0.43 | 0.48 | 0.48 | 2 |
| Task 3 a | 186 | -0.65 | 0.0000 | -0.61 | 0.0000 | 0.42 | 0.48 | 0.48 | 2 |
| Task 2 v Distance effect | 192 | 0.47 | 0.0000 | 0.46 | 0.0000 | 0.22 | 0.23 | 0.23 | 1 |
| Task 3 Ter | 183 | -0.45 | 0.0000 | -0.51 | 0.0000 | 0.19 | 0.19 | 0.19 | 1 |
| Task 2 Ter Distance effect | 190 | 0.38 | 0.0000 | 0.29 | 0.0000 | 0.14 | 0.16 | 0.15 | 2 |
| Task 1 Ter orienting | 192 | -0.36 | 0.0000 | -0.30 | 0.0000 | 0.13 | 0.15 | 0.14 | 2 |
| Task 2 a Distance effect | 191 | -0.19 | 0.0070 | -0.17 | 0.0158 | 0.03 | 0.03 | 0.06 | 3 |
| Task 1 Ter alerting | 192 | -0.22 | 0.0024 | -0.12 | 0.0926 | 0.04 | 0.08 | 0.07 | 2 |
| Task 1 v executive | 192 | -0.16 | 0.0264 | -0.14 | 0.0542 | 0.02 | 0.02 | 0.01 | 1 |
| Task 3 v Distance effect | 187 | 0.25 | 0.0005 | 0.22 | 0.0026 | 0.06 | 0.06 | 0.06 | 1 |
| Task 3 a Distance effect | 186 | 0.11 | 0.1354 | 0.08 | 0.2727 | 0.01 | 0.01 | 0.02 | N/A |
| Task 1 Ter executive | 192 | -0.23 | 0.0014 | -0.19 | 0.0073 | 0.05 | 0.06 | 0.05 | 1 |
| Task 3 Ter Distance effect | 185 | -0.15 | 0.0380 | -0.05 | 0.4873 | 0.02 | 0.03 | 0.03 | 1 |
| Task 2 Ter SNARC effect | 191 | 0.08 | 0.2455 | 0.03 | 0.7209 | 0.00 | 0.00 | -0.01 | N/A |
| Task 1 v alerting | 192 | 0.00 | 0.9658 | 0.02 | 0.8296 | -0.01 | 0.00 | -0.01 | N/A |
| Task 1 v orienting | 191 | -0.23 | 0.0015 | -0.21 | 0.0030 | 0.05 | 0.05 | 0.04 | 1 |
| Task 2 a SNARC effect | 192 | -0.13 | 0.0643 | -0.07 | 0.3469 | 0.01 | 0.02 | 0.01 | N/A |
| Task 1 a orienting | 193 | 0.14 | 0.0589 | 0.06 | 0.4067 | 0.01 | 0.01 | 0.01 | N/A |
| Task 1 a executive | 190 | -0.05 | 0.4675 | -0.03 | 0.6358 | 0.00 | -0.01 | 0.01 | 3 |
| Task 2 v SNARC effect | 192 | -0.12 | 0.0914 | -0.12 | 0.1012 | 0.01 | 0.05 | 0.05 | 2 |
| Task 1 a alerting | 192 | -0.09 | 0.2115 | -0.10 | 0.1601 | 0.00 | 0.01 | 0.02 | N/A |
