# Supplementary material for: Dissecting the chain of information processing and its interplay with neurochemicals and fluid intelligence across development
Source: eLife. 2023 Sep 29;12:e84086. doi: 10.7554/eLife.84086 (PMC10541179; doi:10.7554/eLife.84086)
Supplement: Supplementary file 5. — The first column denotes the task (i.e., Task 1, Task 2, or Task 3), the second column denotes the assessment where “A1” concerns the first assessment analyses corresponding to Equation 3, “A2” concerns the second assessment analyses corresponding to Equation 4, and “Prediction” concerns predicting behaviour during the second assessment based on neurochemicals during the first assessment corresponding to Equation 5. The third column has three names separated by underscores, the first name corresponds to the region and the neurochemical used where IPS=intraparietal sulcus and MFG=middle frontal gyrus, and GLU=glutamate, GABA=gamma-Aminobutyric acid and NAA=N-acetylaspartate, the third name corresponds to the diffusion parameter that was used as the dependent variable, and the second name corresponds to the way each diffusion parameter was calculated where overall=the diffusion parameter was calculated across all the trials, AL=alerting network, OR=orienting network, EX=executive network, DISTANCE=the effect of distance, SNARC=the effect of SNARC. For the rest of the columns (df=degrees of freedom, β=standardized coefficient, PBO=bootstrapped P-value), where β (column 5) and PBO (column 6) correspond to the neurochemical*age interaction and β (column 7) and PBO (column 8) correspond to the main effect of the neurochemical. The column “max_VIF” shows the maximum variance inflation factor assessing multicollinearity, and the column “SW” and “SW_P” shows the Shapiro-Wilk statistic and Shapiro-Wilk P-value, respectively, assessing the normality of the residuals in each model. The column “int_R2” is the adjusted R-squared of the model, and the column “non_int_R2” is the adjusted R-squared of the same model, but when omitting the interaction predictor and the column “delta_R2” is the difference between “int_R2” and “non_int_R2”. Of note, the variance inflation factor in the “Prediction” analyses was calculated, including all predictors apart from the predictor “age during t [file elife-84086-supp5.docx]

**Supplementary File 5.** Statistical results of neurochemicals in tracking behavioural performance. The first column denotes the task (i.e., **Task 1**, **Task 2,** or **Task 3**), the second column denotes the assessment where “A1” concerns the first assessment analyses corresponding to eq1, “A2” concerns the second assessment analyses corresponding to eq2, and “Prediction” concerns predicting behaviour during the second assessment based on neurochemicals during the first assessment corresponding to eq3. The third column has three names separated by underscores, the first name corresponds to the region and the neurochemical used where IPS=intraparietal sulcus and MFG=middle frontal gyrus, and GLU=glutamate, GABA=gamma-Aminobutyric acid and NAA=N-acetylaspartate, the third name corresponds to the diffusion parameter that was used as the dependent variable, and the second name corresponds to the way each diffusion parameter was calculated where overall=the diffusion parameter was calculated across all the trials, AL=alerting network, OR=orienting network, EX=executive network, DISTANCE=the effect of distance, SNARC= the effect of SNARC. For the rest of the columns (df=degrees of freedom, β=standardized coefficient, P_BO_=bootstrapped P-value), where β (column 5) and P_BO_ (column 6) correspond to the neurochemical*age interaction and β (column 7) and P_BO_ (column 8) correspond to the main effect of the neurochemical. The column “max_VIF” shows the maximum variance inflation factor assessing multicollinearity, and the column “SW” and “SW_P” shows the Shapiro-Wilk statistic and Shapiro-Wilk P-value, respectively, assessing the normality of the residuals in each model. The column “int_R^2^” is the adjusted R-squared of the model, and the column “non_int_R^2^” is the adjusted R-squared of the same model, but when omitting the interaction predictor and the column “delta_R^2^” is the difference between “int_R^2^” and “non_int_R^2^”. Of note, the variance inflation factor in the “Prediction” analyses was calculated, including all predictors apart from the predictor “age during the second assessment”, as this predictor is bound to be positively correlated to the predictor “age during the first assessment”.

|  |  |  | **df** | **β** | **P_BO_** | **β** | **P_BO_** | **max_VIF** | **SW** | **SW_P** | **int_R^2^** | **non_int_R^2^** | **delta_R^2^** |
| --- | --- | --- | --- | --- | --- | --- | --- | --- | --- | --- | --- | --- | --- |
| Task 1 | A1 | IPSGLU_overall_MeanDriftRate | 254 | 0.02 | 6.3E-01 | -0.14 | 2.1E-03 | 4.51 | 0.99 | 0.17 | 0.67 | 0.67 | 0.00 |
| Task 1 | A1 | IPSGLU_AL_MeanDriftRate | 252 | -0.04 | 5.1E-01 | -0.05 | 4.9E-01 | 1.70 | 0.98 | 0.00 | 0.26 | 0.26 | 0.00 |
| Task 1 | A1 | IPSGLU_OR_MeanDriftRate | 254 | 0.03 | 5.6E-01 | 0.01 | 8.4E-01 | 1.76 | 0.99 | 0.02 | 0.41 | 0.41 | 0.00 |
| Task 1 | A1 | IPSGLU_EX_MeanDriftRate | 256 | 0.02 | 8.1E-01 | -0.03 | 6.8E-01 | 1.73 | 0.97 | 0.00 | 0.20 | 0.20 | 0.00 |
| Task 1 | A1 | IPSGABA_overall_MeanDriftRate | 254 | 0.03 | 4.3E-01 | -0.05 | 1.5E-01 | 4.06 | 0.99 | 0.18 | 0.66 | 0.66 | 0.00 |
| Task 1 | A1 | IPSGABA_AL_MeanDriftRate | 253 | 0.00 | 9.5E-01 | 0.08 | 2.0E-01 | 1.45 | 0.98 | 0.01 | 0.25 | 0.25 | 0.00 |
| Task 1 | A1 | IPSGABA_OR_MeanDriftRate | 254 | -0.04 | 5.2E-01 | -0.06 | 3.2E-01 | 1.59 | 0.99 | 0.02 | 0.42 | 0.42 | 0.00 |
| Task 1 | A1 | IPSGABA_EX_MeanDriftRate | 256 | 0.02 | 8.2E-01 | 0.04 | 5.4E-01 | 1.43 | 0.97 | 0.00 | 0.21 | 0.21 | 0.00 |
| Task 1 | A1 | IPSNAA_overall_MeanDriftRate | 255 | -0.04 | 3.5E-01 | -0.07 | 1.1E-01 | 4.02 | 0.99 | 0.11 | 0.66 | 0.66 | 0.00 |
| Task 1 | A1 | IPSNAA_AL_MeanDriftRate | 253 | 0.04 | 5.9E-01 | -0.05 | 4.0E-01 | 1.39 | 0.98 | 0.00 | 0.26 | 0.26 | 0.00 |
| Task 1 | A1 | IPSNAA_OR_MeanDriftRate | 255 | -0.03 | 6.5E-01 | 0.02 | 7.1E-01 | 1.58 | 0.99 | 0.03 | 0.41 | 0.41 | 0.00 |
| Task 1 | A1 | IPSNAA_EX_MeanDriftRate | 257 | -0.01 | 8.4E-01 | -0.01 | 8.6E-01 | 1.38 | 0.97 | 0.00 | 0.20 | 0.20 | 0.00 |
| Task 1 | A1 | MFGGLU_overall_MeanDriftRate | 250 | 0.07 | 6.3E-02 | -0.04 | 3.5E-01 | 4.36 | 0.99 | 0.35 | 0.68 | 0.67 | 0.00 |
| Task 1 | A1 | MFGGLU_AL_MeanDriftRate | 250 | 0.08 | 1.8E-01 | -0.01 | 8.5E-01 | 1.54 | 0.98 | 0.01 | 0.25 | 0.25 | 0.00 |
| Task 1 | A1 | MFGGLU_OR_MeanDriftRate | 251 | -0.06 | 2.9E-01 | -0.01 | 9.1E-01 | 1.61 | 0.99 | 0.02 | 0.39 | 0.39 | 0.00 |
| Task 1 | A1 | MFGGLU_EX_MeanDriftRate | 253 | -0.01 | 9.0E-01 | 0.02 | 7.7E-01 | 1.53 | 0.97 | 0.00 | 0.20 | 0.20 | 0.00 |
| Task 1 | A1 | MFGGABA_overall_MeanDriftRate | 244 | 0.05 | 2.0E-01 | -0.03 | 5.0E-01 | 3.93 | 0.99 | 0.25 | 0.67 | 0.67 | 0.00 |
| Task 1 | A1 | MFGGABA_AL_MeanDriftRate | 243 | 0.00 | 9.6E-01 | -0.04 | 5.0E-01 | 1.36 | 0.98 | 0.01 | 0.25 | 0.25 | 0.00 |
| Task 1 | A1 | MFGGABA_OR_MeanDriftRate | 245 | 0.04 | 5.0E-01 | -0.10 | 8.1E-02 | 1.62 | 0.99 | 0.04 | 0.40 | 0.40 | 0.00 |
| Task 1 | A1 | MFGGABA_EX_MeanDriftRate | 247 | -0.05 | 4.0E-01 | 0.03 | 6.7E-01 | 1.45 | 0.97 | 0.00 | 0.21 | 0.21 | 0.00 |
| Task 1 | A1 | MFGNAA_overall_MeanDriftRate | 249 | 0.01 | 8.4E-01 | 0.01 | 8.9E-01 | 3.86 | 0.99 | 0.16 | 0.67 | 0.67 | 0.00 |
| Task 1 | A1 | MFGNAA_AL_MeanDriftRate | 248 | 0.08 | 1.8E-01 | -0.01 | 9.1E-01 | 1.53 | 0.98 | 0.00 | 0.26 | 0.26 | 0.00 |
| Task 1 | A1 | MFGNAA_OR_MeanDriftRate | 250 | -0.10 | 6.5E-02 | -0.08 | 1.5E-01 | 1.62 | 0.99 | 0.05 | 0.40 | 0.40 | 0.01 |
| Task 1 | A1 | MFGNAA_EX_MeanDriftRate | 252 | 0.03 | 6.3E-01 | -0.04 | 5.4E-01 | 1.45 | 0.97 | 0.00 | 0.20 | 0.20 | 0.00 |
| Task 1 | A1 | IPSGLU_overall_BoundarySeparation | 254 | 0.00 | 9.3E-01 | -0.04 | 3.8E-01 | 4.21 | 0.99 | 0.12 | 0.67 | 0.67 | 0.00 |
| Task 1 | A1 | IPSGLU_AL_BoundarySeparation | 251 | 0.09 | 1.3E-01 | -0.04 | 5.2E-01 | 1.71 | 0.98 | 0.00 | 0.41 | 0.41 | 0.00 |
| Task 1 | A1 | IPSGLU_OR_BoundarySeparation | 253 | 0.00 | 9.6E-01 | -0.01 | 8.4E-01 | 1.73 | 0.99 | 0.04 | 0.53 | 0.53 | 0.00 |
| Task 1 | A1 | IPSGLU_EX_BoundarySeparation | 256 | 0.07 | 3.2E-01 | -0.12 | 4.2E-02 | 1.72 | 0.99 | 0.40 | 0.36 | 0.36 | 0.00 |
| Task 1 | A1 | IPSGABA_overall_BoundarySeparation | 254 | 0.05 | 2.3E-01 | -0.05 | 2.2E-01 | 4.10 | 0.99 | 0.12 | 0.66 | 0.66 | 0.00 |
| Task 1 | A1 | IPSGABA_AL_BoundarySeparation | 252 | -0.06 | 4.0E-01 | 0.01 | 8.9E-01 | 1.44 | 0.98 | 0.00 | 0.38 | 0.38 | 0.00 |
| Task 1 | A1 | IPSGABA_OR_BoundarySeparation | 254 | 0.01 | 8.6E-01 | -0.07 | 1.4E-01 | 1.48 | 0.99 | 0.03 | 0.53 | 0.53 | 0.00 |
| Task 1 | A1 | IPSGABA_EX_BoundarySeparation | 256 | -0.01 | 8.7E-01 | -0.07 | 2.8E-01 | 1.47 | 1.00 | 0.62 | 0.35 | 0.35 | 0.00 |
| Task 1 | A1 | IPSNAA_overall_BoundarySeparation | 255 | 0.02 | 6.4E-01 | -0.10 | 7.9E-03 | 4.00 | 0.99 | 0.06 | 0.68 | 0.68 | 0.00 |
| Task 1 | A1 | IPSNAA_AL_BoundarySeparation | 251 | 0.02 | 7.9E-01 | -0.03 | 5.6E-01 | 1.37 | 0.98 | 0.01 | 0.42 | 0.42 | 0.00 |
| Task 1 | A1 | IPSNAA_OR_BoundarySeparation | 255 | 0.00 | 1.0E+00 | -0.06 | 2.5E-01 | 1.40 | 0.99 | 0.01 | 0.52 | 0.52 | 0.00 |
| Task 1 | A1 | IPSNAA_EX_BoundarySeparation | 257 | 0.01 | 8.5E-01 | -0.10 | 8.9E-02 | 1.41 | 0.99 | 0.39 | 0.36 | 0.36 | 0.00 |
| Task 1 | A1 | MFGGLU_overall_BoundarySeparation | 250 | -0.05 | 3.8E-01 | 0.01 | 9.0E-01 | 4.39 | 0.99 | 0.05 | 0.66 | 0.66 | 0.00 |
| Task 1 | A1 | MFGGLU_AL_BoundarySeparation | 248 | 0.00 | 9.5E-01 | 0.02 | 7.9E-01 | 1.53 | 0.98 | 0.00 | 0.37 | 0.37 | 0.00 |
| Task 1 | A1 | MFGGLU_OR_BoundarySeparation | 250 | -0.10 | 1.3E-01 | 0.00 | 9.8E-01 | 1.53 | 0.99 | 0.03 | 0.56 | 0.56 | 0.01 |
| Task 1 | A1 | MFGGLU_EX_BoundarySeparation | 253 | -0.05 | 4.3E-01 | -0.09 | 1.0E-01 | 1.54 | 0.99 | 0.26 | 0.39 | 0.39 | 0.00 |
| Task 1 | A1 | MFGGABA_overall_BoundarySeparation | 244 | 0.00 | 9.9E-01 | 0.00 | 9.4E-01 | 4.04 | 0.99 | 0.10 | 0.66 | 0.66 | 0.00 |
| Task 1 | A1 | MFGGABA_AL_BoundarySeparation | 242 | 0.01 | 8.5E-01 | -0.04 | 4.8E-01 | 1.30 | 0.98 | 0.00 | 0.37 | 0.37 | 0.00 |
| Task 1 | A1 | MFGGABA_OR_BoundarySeparation | 243 | 0.14 | 1.7E-02 | -0.07 | 1.8E-01 | 1.29 | 0.99 | 0.38 | 0.58 | 0.57 | 0.01 |
| Task 1 | A1 | MFGGABA_EX_BoundarySeparation | 247 | 0.02 | 8.2E-01 | 0.01 | 9.1E-01 | 1.28 | 0.99 | 0.25 | 0.38 | 0.38 | 0.00 |
| Task 1 | A1 | MFGNAA_overall_BoundarySeparation | 249 | -0.02 | 7.5E-01 | -0.05 | 2.1E-01 | 3.98 | 0.99 | 0.02 | 0.66 | 0.66 | 0.00 |
| Task 1 | A1 | MFGNAA_AL_BoundarySeparation | 247 | -0.08 | 2.0E-01 | 0.01 | 7.7E-01 | 1.52 | 0.98 | 0.00 | 0.37 | 0.37 | 0.00 |
| Task 1 | A1 | MFGNAA_OR_BoundarySeparation | 248 | -0.11 | 5.0E-02 | -0.05 | 2.7E-01 | 1.44 | 0.99 | 0.17 | 0.59 | 0.57 | 0.01 |
| Task 1 | A1 | MFGNAA_EX_BoundarySeparation | 252 | 0.02 | 7.5E-01 | -0.03 | 6.4E-01 | 1.46 | 0.99 | 0.20 | 0.39 | 0.39 | 0.00 |
| Task 1 | A1 | IPSGLU_overall_NonDecisionTime | 252 | -0.22 | 3.8E-05 | -0.02 | 7.1E-01 | 3.81 | 0.98 | 0.00 | 0.68 | 0.64 | 0.04 |
| Task 1 | A1 | IPSGLU_AL_NonDecisionTime | 247 | 0.05 | 5.2E-01 | 0.05 | 4.4E-01 | 1.63 | 0.98 | 0.01 | 0.39 | 0.40 | 0.00 |
| Task 1 | A1 | IPSGLU_OR_NonDecisionTime | 253 | 0.02 | 8.3E-01 | 0.04 | 4.8E-01 | 1.63 | 0.98 | 0.00 | 0.43 | 0.43 | 0.00 |
| Task 1 | A1 | IPSGLU_EX_NonDecisionTime | 254 | 0.20 | 5.1E-03 | -0.06 | 3.1E-01 | 1.68 | 0.98 | 0.01 | 0.32 | 0.29 | 0.03 |
| Task 1 | A1 | IPSGABA_overall_NonDecisionTime | 252 | 0.23 | 1.1E-06 | -0.10 | 8.9E-03 | 3.71 | 0.97 | 0.00 | 0.68 | 0.64 | 0.04 |
| Task 1 | A1 | IPSGABA_AL_NonDecisionTime | 248 | -0.02 | 8.1E-01 | 0.01 | 9.2E-01 | 1.36 | 0.99 | 0.01 | 0.39 | 0.39 | 0.00 |
| Task 1 | A1 | IPSGABA_OR_NonDecisionTime | 253 | 0.00 | 9.4E-01 | -0.03 | 5.8E-01 | 1.60 | 0.98 | 0.01 | 0.44 | 0.44 | 0.00 |
| Task 1 | A1 | IPSGABA_EX_NonDecisionTime | 254 | 0.07 | 3.5E-01 | -0.06 | 2.8E-01 | 1.43 | 0.98 | 0.00 | 0.29 | 0.29 | 0.00 |
| Task 1 | A1 | IPSNAA_overall_NonDecisionTime | 252 | 0.25 | 7.9E-09 | -0.17 | 2.5E-06 | 3.57 | 0.97 | 0.00 | 0.71 | 0.66 | 0.06 |
| Task 1 | A1 | IPSNAA_AL_NonDecisionTime | 248 | -0.06 | 3.1E-01 | 0.06 | 1.9E-01 | 1.33 | 0.98 | 0.01 | 0.40 | 0.40 | 0.00 |
| Task 1 | A1 | IPSNAA_OR_NonDecisionTime | 254 | -0.01 | 9.2E-01 | 0.01 | 9.3E-01 | 1.57 | 0.98 | 0.00 | 0.43 | 0.43 | 0.00 |
| Task 1 | A1 | IPSNAA_EX_NonDecisionTime | 254 | 0.08 | 2.1E-01 | -0.04 | 4.8E-01 | 1.37 | 0.98 | 0.01 | 0.30 | 0.30 | 0.00 |
| Task 1 | A1 | MFGGLU_overall_NonDecisionTime | 249 | -0.11 | 5.6E-02 | -0.05 | 3.0E-01 | 3.60 | 0.97 | 0.00 | 0.65 | 0.64 | 0.01 |
| Task 1 | A1 | MFGGLU_AL_NonDecisionTime | 243 | 0.03 | 7.1E-01 | 0.04 | 5.1E-01 | 1.42 | 0.98 | 0.01 | 0.40 | 0.40 | 0.00 |
| Task 1 | A1 | MFGGLU_OR_NonDecisionTime | 250 | -0.03 | 7.2E-01 | 0.00 | 9.5E-01 | 1.58 | 0.98 | 0.01 | 0.43 | 0.43 | 0.00 |
| Task 1 | A1 | MFGGLU_EX_NonDecisionTime | 251 | 0.04 | 5.4E-01 | 0.01 | 9.2E-01 | 1.51 | 0.98 | 0.01 | 0.33 | 0.33 | 0.00 |
| Task 1 | A1 | MFGGABA_overall_NonDecisionTime | 243 | 0.26 | 3.9E-08 | -0.15 | 3.9E-05 | 3.65 | 0.98 | 0.00 | 0.70 | 0.65 | 0.05 |
| Task 1 | A1 | MFGGABA_AL_NonDecisionTime | 237 | -0.06 | 5.1E-01 | 0.06 | 3.3E-01 | 1.28 | 0.99 | 0.05 | 0.40 | 0.40 | 0.00 |
| Task 1 | A1 | MFGGABA_OR_NonDecisionTime | 244 | -0.02 | 7.9E-01 | 0.01 | 8.4E-01 | 1.61 | 0.98 | 0.01 | 0.43 | 0.43 | 0.00 |
| Task 1 | A1 | MFGGABA_EX_NonDecisionTime | 245 | 0.08 | 3.7E-01 | 0.03 | 6.5E-01 | 1.28 | 0.99 | 0.03 | 0.33 | 0.32 | 0.00 |
| Task 1 | A1 | MFGNAA_overall_NonDecisionTime | 246 | 0.32 | 7.5E-19 | -0.19 | 5.7E-10 | 3.45 | 0.99 | 0.03 | 0.75 | 0.66 | 0.10 |
| Task 1 | A1 | MFGNAA_AL_NonDecisionTime | 242 | -0.06 | 3.4E-01 | 0.09 | 7.6E-02 | 1.43 | 0.98 | 0.01 | 0.41 | 0.40 | 0.00 |
| Task 1 | A1 | MFGNAA_OR_NonDecisionTime | 249 | -0.07 | 2.8E-01 | -0.02 | 7.0E-01 | 1.63 | 0.98 | 0.01 | 0.44 | 0.43 | 0.00 |
| Task 1 | A1 | MFGNAA_EX_NonDecisionTime | 249 | 0.06 | 3.7E-01 | 0.01 | 8.4E-01 | 1.44 | 0.99 | 0.04 | 0.34 | 0.34 | 0.00 |
| Task 2 | A1 | IPSGLU_overall_MeanDriftRate | 245 | 0.08 | 6.1E-02 | -0.01 | 8.1E-01 | 4.13 | 0.99 | 0.25 | 0.71 | 0.71 | 0.00 |
| Task 2 | A1 | IPSGLU_SNARC_MeanDriftRate | 245 | -0.05 | 4.5E-01 | -0.07 | 3.7E-01 | 1.65 | 0.99 | 0.03 | 0.05 | 0.05 | 0.00 |
| Task 2 | A1 | IPSGLU_DISTANCE_MeanDriftRate | 242 | 0.13 | 4.7E-02 | 0.10 | 1.1E-01 | 1.73 | 0.98 | 0.01 | 0.25 | 0.24 | 0.01 |
| Task 2 | A1 | IPSGABA_overall_MeanDriftRate | 245 | -0.04 | 3.6E-01 | 0.00 | 9.8E-01 | 3.47 | 0.99 | 0.19 | 0.71 | 0.71 | 0.00 |
| Task 2 | A1 | IPSGABA_SNARC_MeanDriftRate | 244 | 0.05 | 3.9E-01 | 0.00 | 9.5E-01 | 1.48 | 0.99 | 0.04 | 0.05 | 0.06 | 0.00 |
| Task 2 | A1 | IPSGABA_DISTANCE_MeanDriftRate | 243 | 0.00 | 9.7E-01 | 0.08 | 1.2E-01 | 1.57 | 0.98 | 0.00 | 0.23 | 0.23 | 0.00 |
| Task 2 | A1 | IPSNAA_overall_MeanDriftRate | 246 | 0.03 | 3.9E-01 | 0.01 | 8.4E-01 | 3.42 | 0.99 | 0.24 | 0.71 | 0.71 | 0.00 |
| Task 2 | A1 | IPSNAA_SNARC_MeanDriftRate | 246 | 0.01 | 8.5E-01 | -0.05 | 4.8E-01 | 1.46 | 0.99 | 0.01 | 0.05 | 0.05 | 0.00 |
| Task 2 | A1 | IPSNAA_DISTANCE_MeanDriftRate | 244 | 0.00 | 9.9E-01 | 0.08 | 2.4E-01 | 1.58 | 0.98 | 0.00 | 0.23 | 0.24 | 0.00 |
| Task 2 | A1 | MFGGLU_overall_MeanDriftRate | 242 | 0.04 | 3.1E-01 | -0.03 | 4.5E-01 | 4.04 | 0.99 | 0.21 | 0.70 | 0.70 | 0.00 |
| Task 2 | A1 | MFGGLU_SNARC_MeanDriftRate | 241 | 0.04 | 6.1E-01 | 0.00 | 9.7E-01 | 1.53 | 0.99 | 0.06 | 0.06 | 0.06 | 0.00 |
| Task 2 | A1 | MFGGLU_DISTANCE_MeanDriftRate | 240 | 0.10 | 1.3E-01 | 0.11 | 9.9E-02 | 1.75 | 0.99 | 0.04 | 0.26 | 0.25 | 0.00 |
| Task 2 | A1 | MFGGABA_overall_MeanDriftRate | 236 | 0.03 | 4.8E-01 | -0.04 | 3.2E-01 | 3.48 | 0.99 | 0.14 | 0.70 | 0.70 | 0.00 |
| Task 2 | A1 | MFGGABA_SNARC_MeanDriftRate | 235 | -0.07 | 3.1E-01 | -0.02 | 7.4E-01 | 1.45 | 0.99 | 0.07 | 0.06 | 0.06 | 0.00 |
| Task 2 | A1 | MFGGABA_DISTANCE_MeanDriftRate | 235 | 0.01 | 9.2E-01 | 0.00 | 9.7E-01 | 1.63 | 0.98 | 0.00 | 0.24 | 0.24 | 0.00 |
| Task 2 | A1 | MFGNAA_overall_MeanDriftRate | 241 | 0.03 | 3.6E-01 | 0.02 | 6.4E-01 | 3.39 | 0.99 | 0.31 | 0.70 | 0.70 | 0.00 |
| Task 2 | A1 | MFGNAA_SNARC_MeanDriftRate | 241 | 0.01 | 8.4E-01 | -0.05 | 4.6E-01 | 1.48 | 0.99 | 0.03 | 0.05 | 0.05 | 0.00 |
| Task 2 | A1 | MFGNAA_DISTANCE_MeanDriftRate | 240 | 0.00 | 9.9E-01 | 0.08 | 2.0E-01 | 1.76 | 0.98 | 0.00 | 0.25 | 0.25 | 0.00 |
| Task 2 | A1 | IPSGLU_overall_BoundarySeparation | 241 | 0.01 | 8.3E-01 | -0.13 | 1.6E-03 | 5.53 | 0.96 | 0.00 | 0.61 | 0.62 | 0.00 |
| Task 2 | A1 | IPSGLU_SNARC_BoundarySeparation | 243 | 0.05 | 4.7E-01 | -0.01 | 8.8E-01 | 1.64 | 0.99 | 0.19 | 0.40 | 0.40 | 0.00 |
| Task 2 | A1 | IPSGLU_DISTANCE_BoundarySeparation | 240 | -0.15 | 2.3E-02 | 0.17 | 3.0E-03 | 1.83 | 0.99 | 0.08 | 0.39 | 0.37 | 0.02 |
| Task 2 | A1 | IPSGABA_overall_BoundarySeparation | 241 | -0.03 | 6.4E-01 | 0.01 | 8.3E-01 | 5.12 | 0.97 | 0.00 | 0.61 | 0.61 | 0.00 |
| Task 2 | A1 | IPSGABA_SNARC_BoundarySeparation | 243 | -0.01 | 8.5E-01 | -0.02 | 6.7E-01 | 1.43 | 0.99 | 0.38 | 0.40 | 0.40 | 0.00 |
| Task 2 | A1 | IPSGABA_DISTANCE_BoundarySeparation | 242 | -0.02 | 8.8E-01 | 0.02 | 8.2E-01 | 1.68 | 0.99 | 0.07 | 0.29 | 0.29 | 0.00 |
| Task 2 | A1 | IPSNAA_overall_BoundarySeparation | 243 | 0.12 | 4.5E-02 | -0.14 | 1.7E-03 | 5.19 | 0.97 | 0.00 | 0.63 | 0.62 | 0.01 |
| Task 2 | A1 | IPSNAA_SNARC_BoundarySeparation | 244 | 0.06 | 3.6E-01 | 0.00 | 9.9E-01 | 1.34 | 0.99 | 0.14 | 0.40 | 0.40 | 0.00 |
| Task 2 | A1 | IPSNAA_DISTANCE_BoundarySeparation | 243 | -0.16 | 1.7E-02 | 0.15 | 5.0E-03 | 1.63 | 0.99 | 0.31 | 0.33 | 0.30 | 0.02 |
| Task 2 | A1 | MFGGLU_overall_BoundarySeparation | 237 | 0.04 | 4.8E-01 | -0.10 | 2.3E-02 | 5.34 | 0.97 | 0.00 | 0.62 | 0.62 | 0.00 |
| Task 2 | A1 | MFGGLU_SNARC_BoundarySeparation | 240 | 0.04 | 6.3E-01 | -0.02 | 8.2E-01 | 1.55 | 0.99 | 0.28 | 0.39 | 0.39 | 0.00 |
| Task 2 | A1 | MFGGLU_DISTANCE_BoundarySeparation | 241 | -0.21 | 9.1E-04 | 0.14 | 1.5E-02 | 1.96 | 0.99 | 0.04 | 0.33 | 0.30 | 0.03 |
| Task 2 | A1 | MFGGABA_overall_BoundarySeparation | 231 | 0.03 | 6.5E-01 | 0.01 | 8.0E-01 | 4.95 | 0.97 | 0.00 | 0.61 | 0.61 | 0.00 |
| Task 2 | A1 | MFGGABA_SNARC_BoundarySeparation | 234 | 0.05 | 5.3E-01 | -0.02 | 7.6E-01 | 1.36 | 0.99 | 0.20 | 0.39 | 0.39 | 0.00 |
| Task 2 | A1 | MFGGABA_DISTANCE_BoundarySeparation | 234 | -0.08 | 3.2E-01 | 0.04 | 4.6E-01 | 1.58 | 0.99 | 0.05 | 0.31 | 0.31 | 0.00 |
| Task 2 | A1 | MFGNAA_overall_BoundarySeparation | 237 | 0.07 | 2.1E-01 | -0.11 | 1.1E-02 | 4.87 | 0.97 | 0.00 | 0.62 | 0.61 | 0.00 |
| Task 2 | A1 | MFGNAA_SNARC_BoundarySeparation | 239 | 0.07 | 2.9E-01 | 0.00 | 9.8E-01 | 1.45 | 0.99 | 0.16 | 0.40 | 0.39 | 0.00 |
| Task 2 | A1 | MFGNAA_DISTANCE_BoundarySeparation | 240 | -0.24 | 3.9E-05 | 0.15 | 1.0E-02 | 1.67 | 0.99 | 0.51 | 0.35 | 0.29 | 0.05 |
| Task 2 | A1 | IPSGLU_overall_NonDecisionTime | 240 | -0.17 | 6.3E-05 | 0.02 | 6.2E-01 | 4.45 | 0.98 | 0.00 | 0.71 | 0.69 | 0.02 |
| Task 2 | A1 | IPSGLU_SNARC_NonDecisionTime | 240 | -0.05 | 4.6E-01 | 0.01 | 9.4E-01 | 1.66 | 0.98 | 0.00 | 0.37 | 0.37 | 0.00 |
| Task 2 | A1 | IPSGLU_DISTANCE_NonDecisionTime | 239 | 0.07 | 3.8E-01 | 0.04 | 5.1E-01 | 1.81 | 0.95 | 0.00 | 0.44 | 0.44 | 0.00 |
| Task 2 | A1 | IPSGABA_overall_NonDecisionTime | 240 | 0.18 | 1.9E-04 | -0.10 | 1.4E-02 | 4.08 | 0.98 | 0.01 | 0.74 | 0.72 | 0.03 |
| Task 2 | A1 | IPSGABA_SNARC_NonDecisionTime | 240 | 0.06 | 4.8E-01 | 0.00 | 9.6E-01 | 1.45 | 0.98 | 0.00 | 0.37 | 0.37 | 0.00 |
| Task 2 | A1 | IPSGABA_DISTANCE_NonDecisionTime | 239 | 0.01 | 9.3E-01 | 0.00 | 9.7E-01 | 1.61 | 0.95 | 0.00 | 0.42 | 0.42 | 0.00 |
| Task 2 | A1 | IPSNAA_overall_NonDecisionTime | 240 | 0.13 | 1.2E-02 | -0.07 | 1.0E-01 | 3.96 | 0.98 | 0.01 | 0.73 | 0.71 | 0.01 |
| Task 2 | A1 | IPSNAA_SNARC_NonDecisionTime | 241 | 0.02 | 8.0E-01 | 0.01 | 8.4E-01 | 1.38 | 0.98 | 0.00 | 0.37 | 0.37 | 0.00 |
| Task 2 | A1 | IPSNAA_DISTANCE_NonDecisionTime | 241 | -0.05 | 4.1E-01 | 0.10 | 2.3E-02 | 1.57 | 0.94 | 0.00 | 0.46 | 0.46 | 0.00 |
| Task 2 | A1 | MFGGLU_overall_NonDecisionTime | 240 | -0.08 | 1.9E-01 | -0.01 | 8.9E-01 | 4.44 | 0.97 | 0.00 | 0.70 | 0.70 | 0.00 |
| Task 2 | A1 | MFGGLU_SNARC_NonDecisionTime | 237 | -0.13 | 6.8E-02 | -0.01 | 8.6E-01 | 1.57 | 0.98 | 0.00 | 0.39 | 0.38 | 0.01 |
| Task 2 | A1 | MFGGLU_DISTANCE_NonDecisionTime | 238 | 0.01 | 8.9E-01 | 0.05 | 3.8E-01 | 1.73 | 0.95 | 0.00 | 0.46 | 0.47 | 0.00 |
| Task 2 | A1 | MFGGABA_overall_NonDecisionTime | 231 | 0.14 | 3.2E-03 | -0.11 | 7.2E-04 | 4.07 | 0.99 | 0.02 | 0.73 | 0.72 | 0.01 |
| Task 2 | A1 | MFGGABA_SNARC_NonDecisionTime | 231 | -0.06 | 4.9E-01 | 0.02 | 8.2E-01 | 1.33 | 0.98 | 0.01 | 0.38 | 0.37 | 0.00 |
| Task 2 | A1 | MFGGABA_DISTANCE_NonDecisionTime | 232 | -0.10 | 1.5E-01 | 0.09 | 1.0E-01 | 1.50 | 0.96 | 0.00 | 0.47 | 0.47 | 0.01 |
| Task 2 | A1 | MFGNAA_overall_NonDecisionTime | 237 | 0.16 | 5.7E-04 | -0.06 | 9.6E-02 | 4.03 | 0.98 | 0.01 | 0.74 | 0.72 | 0.02 |
| Task 2 | A1 | MFGNAA_SNARC_NonDecisionTime | 236 | -0.08 | 3.1E-01 | 0.02 | 7.9E-01 | 1.43 | 0.98 | 0.00 | 0.38 | 0.38 | 0.00 |
| Task 2 | A1 | MFGNAA_DISTANCE_NonDecisionTime | 237 | -0.08 | 1.8E-01 | 0.06 | 2.4E-01 | 1.63 | 0.95 | 0.00 | 0.47 | 0.47 | 0.00 |
| Task 3 | A1 | IPSGLU_overall_MeanDriftRate | 227 | 0.01 | 8.8E-01 | -0.08 | 7.9E-02 | 2.47 | 0.99 | 0.38 | 0.63 | 0.63 | 0.00 |
| Task 3 | A1 | IPSGLU_DISTANCE_MeanDriftRate | 225 | -0.09 | 2.1E-01 | -0.07 | 4.0E-01 | 1.87 | 0.99 | 0.29 | 0.08 | 0.07 | 0.00 |
| Task 3 | A1 | IPSGABA_overall_MeanDriftRate | 228 | 0.02 | 7.8E-01 | -0.02 | 6.6E-01 | 2.15 | 0.99 | 0.16 | 0.63 | 0.63 | 0.00 |
| Task 3 | A1 | IPSGABA_DISTANCE_MeanDriftRate | 226 | 0.13 | 4.0E-02 | 0.01 | 8.7E-01 | 1.90 | 0.99 | 0.29 | 0.08 | 0.07 | 0.01 |
| Task 3 | A1 | IPSNAA_overall_MeanDriftRate | 228 | 0.02 | 6.4E-01 | -0.05 | 2.1E-01 | 2.11 | 0.99 | 0.24 | 0.63 | 0.63 | 0.00 |
| Task 3 | A1 | IPSNAA_DISTANCE_MeanDriftRate | 227 | 0.11 | 7.2E-02 | -0.05 | 4.7E-01 | 1.86 | 0.99 | 0.19 | 0.08 | 0.07 | 0.01 |
| Task 3 | A1 | MFGGLU_overall_MeanDriftRate | 223 | 0.10 | 4.8E-02 | -0.04 | 4.3E-01 | 2.46 | 0.99 | 0.21 | 0.62 | 0.61 | 0.01 |
| Task 3 | A1 | MFGGLU_DISTANCE_MeanDriftRate | 221 | -0.10 | 2.1E-01 | -0.11 | 1.5E-01 | 1.81 | 0.99 | 0.18 | 0.09 | 0.09 | 0.00 |
| Task 3 | A1 | MFGGABA_overall_MeanDriftRate | 217 | 0.11 | 2.4E-02 | -0.02 | 6.3E-01 | 2.09 | 0.99 | 0.37 | 0.62 | 0.61 | 0.01 |
| Task 3 | A1 | MFGGABA_DISTANCE_MeanDriftRate | 215 | 0.04 | 5.3E-01 | 0.07 | 3.4E-01 | 1.80 | 0.99 | 0.12 | 0.09 | 0.09 | 0.00 |
| Task 3 | A1 | MFGNAA_overall_MeanDriftRate | 222 | 0.08 | 1.0E-01 | 0.02 | 6.6E-01 | 2.08 | 0.99 | 0.18 | 0.62 | 0.61 | 0.00 |
| Task 3 | A1 | MFGNAA_DISTANCE_MeanDriftRate | 220 | 0.07 | 2.8E-01 | 0.02 | 7.5E-01 | 1.80 | 0.99 | 0.11 | 0.08 | 0.08 | 0.00 |
| Task 3 | A1 | IPSGLU_overall_BoundarySeparation | 225 | -0.05 | 3.6E-01 | -0.08 | 2.0E-01 | 2.86 | 0.99 | 0.06 | 0.43 | 0.43 | 0.00 |
| Task 3 | A1 | IPSGLU_DISTANCE_BoundarySeparation | 225 | 0.06 | 3.9E-01 | 0.00 | 9.5E-01 | 1.67 | 0.99 | 0.04 | 0.50 | 0.50 | 0.00 |
| Task 3 | A1 | IPSGABA_overall_BoundarySeparation | 227 | 0.18 | 1.4E-03 | -0.14 | 1.1E-02 | 2.79 | 0.99 | 0.10 | 0.48 | 0.45 | 0.03 |
| Task 3 | A1 | IPSGABA_DISTANCE_BoundarySeparation | 224 | 0.11 | 8.3E-02 | -0.02 | 6.1E-01 | 1.36 | 0.99 | 0.05 | 0.54 | 0.53 | 0.01 |
| Task 3 | A1 | IPSNAA_overall_BoundarySeparation | 225 | 0.22 | 2.1E-05 | -0.17 | 7.8E-04 | 2.74 | 0.99 | 0.09 | 0.50 | 0.45 | 0.04 |
| Task 3 | A1 | IPSNAA_DISTANCE_BoundarySeparation | 226 | 0.01 | 9.4E-01 | 0.03 | 4.9E-01 | 1.30 | 0.98 | 0.02 | 0.50 | 0.50 | 0.00 |
| Task 3 | A1 | MFGGLU_overall_BoundarySeparation | 222 | 0.07 | 3.4E-01 | -0.06 | 3.2E-01 | 2.77 | 0.99 | 0.13 | 0.42 | 0.42 | 0.00 |
| Task 3 | A1 | MFGGLU_DISTANCE_BoundarySeparation | 221 | -0.07 | 3.4E-01 | 0.10 | 7.8E-02 | 1.51 | 0.98 | 0.01 | 0.49 | 0.49 | 0.00 |
| Task 3 | A1 | MFGGABA_overall_BoundarySeparation | 218 | 0.20 | 4.5E-03 | -0.10 | 5.3E-02 | 2.86 | 0.99 | 0.02 | 0.44 | 0.41 | 0.03 |
| Task 3 | A1 | MFGGABA_DISTANCE_BoundarySeparation | 214 | 0.02 | 7.7E-01 | -0.02 | 7.0E-01 | 1.24 | 0.98 | 0.00 | 0.52 | 0.52 | 0.00 |
| Task 3 | A1 | MFGNAA_overall_BoundarySeparation | 220 | 0.24 | 2.4E-05 | -0.09 | 5.7E-02 | 2.75 | 0.99 | 0.28 | 0.47 | 0.42 | 0.05 |
| Task 3 | A1 | MFGNAA_DISTANCE_BoundarySeparation | 219 | 0.05 | 3.7E-01 | 0.02 | 6.5E-01 | 1.32 | 0.98 | 0.00 | 0.52 | 0.52 | 0.00 |
| Task 3 | A1 | IPSGLU_overall_NonDecisionTime | 222 | -0.27 | 5.5E-04 | 0.13 | 5.3E-02 | 2.86 | 0.97 | 0.00 | 0.41 | 0.35 | 0.06 |
| Task 3 | A1 | IPSGLU_DISTANCE_NonDecisionTime | 222 | 0.01 | 9.3E-01 | 0.05 | 2.4E-01 | 1.63 | 0.93 | 0.00 | 0.57 | 0.57 | 0.00 |
| Task 3 | A1 | IPSGABA_overall_NonDecisionTime | 224 | 0.27 | 1.1E-03 | -0.16 | 1.5E-02 | 2.60 | 0.98 | 0.00 | 0.41 | 0.36 | 0.05 |
| Task 3 | A1 | IPSGABA_DISTANCE_NonDecisionTime | 222 | -0.01 | 9.3E-01 | 0.03 | 5.1E-01 | 1.34 | 0.94 | 0.00 | 0.58 | 0.58 | 0.00 |
| Task 3 | A1 | IPSNAA_overall_NonDecisionTime | 224 | 0.21 | 1.3E-03 | -0.12 | 2.0E-02 | 2.51 | 0.96 | 0.00 | 0.39 | 0.35 | 0.04 |
| Task 3 | A1 | IPSNAA_DISTANCE_NonDecisionTime | 224 | -0.04 | 5.6E-01 | 0.05 | 3.5E-01 | 1.25 | 0.92 | 0.00 | 0.54 | 0.54 | 0.00 |
| Task 3 | A1 | MFGGLU_overall_NonDecisionTime | 217 | -0.06 | 4.2E-01 | -0.02 | 7.3E-01 | 2.56 | 0.99 | 0.08 | 0.42 | 0.42 | 0.00 |
| Task 3 | A1 | MFGGLU_DISTANCE_NonDecisionTime | 219 | -0.01 | 9.3E-01 | 0.08 | 1.4E-01 | 1.56 | 0.93 | 0.00 | 0.55 | 0.56 | 0.00 |
| Task 3 | A1 | MFGGABA_overall_NonDecisionTime | 212 | 0.16 | 1.7E-02 | -0.11 | 4.5E-02 | 2.46 | 0.98 | 0.01 | 0.42 | 0.40 | 0.02 |
| Task 3 | A1 | MFGGABA_DISTANCE_NonDecisionTime | 212 | -0.03 | 7.4E-01 | 0.01 | 8.1E-01 | 1.24 | 0.94 | 0.00 | 0.58 | 0.58 | 0.00 |
| Task 3 | A1 | MFGNAA_overall_NonDecisionTime | 218 | 0.23 | 4.7E-05 | -0.19 | 2.6E-05 | 2.46 | 0.96 | 0.00 | 0.45 | 0.39 | 0.05 |
| Task 3 | A1 | MFGNAA_DISTANCE_NonDecisionTime | 218 | -0.03 | 7.0E-01 | 0.04 | 3.7E-01 | 1.29 | 0.93 | 0.00 | 0.55 | 0.55 | 0.00 |
| Task 1 | A2 | IPSGLU_overall_MeanDriftRate | 176 | -0.06 | 2.6E-01 | -0.02 | 7.6E-01 | 3.17 | 0.99 | 0.33 | 0.64 | 0.64 | 0.00 |
| Task 1 | A2 | IPSGLU_AL_MeanDriftRate | 173 | 0.14 | 1.9E-02 | 0.05 | 5.0E-01 | 1.69 | 0.99 | 0.08 | 0.40 | 0.39 | 0.01 |
| Task 1 | A2 | IPSGLU_OR_MeanDriftRate | 171 | 0.02 | 7.4E-01 | -0.08 | 2.6E-01 | 1.72 | 0.98 | 0.01 | 0.33 | 0.33 | 0.00 |
| Task 1 | A2 | IPSGLU_EX_MeanDriftRate | 174 | -0.10 | 1.3E-01 | 0.00 | 9.7E-01 | 2.08 | 0.98 | 0.00 | 0.23 | 0.23 | 0.00 |
| Task 1 | A2 | IPSGABA_overall_MeanDriftRate | 176 | -0.09 | 9.6E-02 | 0.07 | 1.5E-01 | 2.84 | 0.99 | 0.45 | 0.65 | 0.65 | 0.00 |
| Task 1 | A2 | IPSGABA_AL_MeanDriftRate | 173 | 0.07 | 2.3E-01 | 0.04 | 5.5E-01 | 1.57 | 0.99 | 0.19 | 0.39 | 0.39 | 0.00 |
| Task 1 | A2 | IPSGABA_OR_MeanDriftRate | 171 | -0.08 | 3.7E-01 | 0.03 | 6.7E-01 | 1.61 | 0.98 | 0.01 | 0.33 | 0.33 | 0.00 |
| Task 1 | A2 | IPSGABA_EX_MeanDriftRate | 173 | 0.06 | 4.5E-01 | 0.04 | 6.1E-01 | 1.92 | 0.98 | 0.00 | 0.23 | 0.23 | 0.00 |
| Task 1 | A2 | IPSNAA_overall_MeanDriftRate | 176 | 0.00 | 9.3E-01 | -0.03 | 5.8E-01 | 2.82 | 0.99 | 0.19 | 0.64 | 0.64 | 0.00 |
| Task 1 | A2 | IPSNAA_AL_MeanDriftRate | 173 | 0.02 | 7.9E-01 | 0.04 | 5.1E-01 | 1.58 | 0.99 | 0.18 | 0.39 | 0.39 | 0.00 |
| Task 1 | A2 | IPSNAA_OR_MeanDriftRate | 171 | -0.04 | 5.8E-01 | -0.08 | 2.2E-01 | 1.66 | 0.97 | 0.00 | 0.33 | 0.33 | 0.00 |
| Task 1 | A2 | IPSNAA_EX_MeanDriftRate | 174 | 0.00 | 9.5E-01 | -0.09 | 2.0E-01 | 1.93 | 0.97 | 0.00 | 0.23 | 0.23 | 0.00 |
| Task 1 | A2 | MFGGLU_overall_MeanDriftRate | 169 | 0.04 | 5.2E-01 | -0.09 | 9.5E-02 | 3.21 | 0.99 | 0.27 | 0.66 | 0.66 | 0.00 |
| Task 1 | A2 | MFGGLU_AL_MeanDriftRate | 167 | 0.00 | 9.6E-01 | -0.02 | 7.9E-01 | 1.62 | 0.99 | 0.22 | 0.40 | 0.41 | 0.00 |
| Task 1 | A2 | MFGGLU_OR_MeanDriftRate | 163 | -0.02 | 7.6E-01 | 0.07 | 3.2E-01 | 1.53 | 0.98 | 0.01 | 0.38 | 0.39 | 0.00 |
| Task 1 | A2 | MFGGLU_EX_MeanDriftRate | 166 | 0.07 | 3.4E-01 | -0.10 | 2.1E-01 | 1.98 | 0.97 | 0.00 | 0.28 | 0.28 | 0.00 |
| Task 1 | A2 | MFGGABA_overall_MeanDriftRate | 168 | -0.05 | 3.5E-01 | -0.04 | 4.1E-01 | 2.85 | 0.99 | 0.51 | 0.66 | 0.66 | 0.00 |
| Task 1 | A2 | MFGGABA_AL_MeanDriftRate | 166 | -0.04 | 5.0E-01 | 0.01 | 8.5E-01 | 1.63 | 0.99 | 0.24 | 0.40 | 0.40 | 0.00 |
| Task 1 | A2 | MFGGABA_OR_MeanDriftRate | 162 | 0.03 | 6.6E-01 | -0.14 | 3.4E-02 | 1.52 | 0.98 | 0.03 | 0.39 | 0.39 | 0.00 |
| Task 1 | A2 | MFGGABA_EX_MeanDriftRate | 165 | -0.09 | 2.3E-01 | -0.07 | 3.4E-01 | 1.92 | 0.97 | 0.00 | 0.27 | 0.26 | 0.00 |
| Task 1 | A2 | MFGNAA_overall_MeanDriftRate | 168 | 0.03 | 5.8E-01 | -0.08 | 1.7E-01 | 2.76 | 0.99 | 0.28 | 0.68 | 0.68 | 0.00 |
| Task 1 | A2 | MFGNAA_AL_MeanDriftRate | 168 | -0.07 | 3.0E-01 | 0.01 | 8.6E-01 | 1.60 | 0.99 | 0.22 | 0.39 | 0.39 | 0.00 |
| Task 1 | A2 | MFGNAA_OR_MeanDriftRate | 163 | 0.07 | 2.9E-01 | 0.00 | 9.9E-01 | 1.44 | 0.98 | 0.02 | 0.39 | 0.38 | 0.00 |
| Task 1 | A2 | MFGNAA_EX_MeanDriftRate | 166 | 0.05 | 4.9E-01 | -0.12 | 9.2E-02 | 1.98 | 0.97 | 0.00 | 0.28 | 0.28 | 0.00 |
| Task 1 | A2 | IPSGLU_overall_BoundarySeparation | 176 | -0.14 | 2.5E-02 | 0.03 | 5.5E-01 | 3.68 | 0.99 | 0.48 | 0.67 | 0.66 | 0.01 |
| Task 1 | A2 | IPSGLU_AL_BoundarySeparation | 172 | 0.11 | 9.2E-02 | 0.00 | 9.8E-01 | 1.61 | 0.99 | 0.70 | 0.53 | 0.52 | 0.01 |
| Task 1 | A2 | IPSGLU_OR_BoundarySeparation | 171 | 0.03 | 7.1E-01 | -0.04 | 5.8E-01 | 1.73 | 0.97 | 0.00 | 0.48 | 0.49 | 0.00 |
| Task 1 | A2 | IPSGLU_EX_BoundarySeparation | 170 | -0.23 | 2.6E-05 | 0.12 | 5.7E-02 | 1.63 | 0.99 | 0.47 | 0.57 | 0.52 | 0.04 |
| Task 1 | A2 | IPSGABA_overall_BoundarySeparation | 175 | -0.03 | 6.5E-01 | 0.03 | 5.5E-01 | 3.39 | 0.99 | 0.33 | 0.67 | 0.67 | 0.00 |
| Task 1 | A2 | IPSGABA_AL_BoundarySeparation | 172 | -0.08 | 2.4E-01 | 0.02 | 7.2E-01 | 1.32 | 0.99 | 0.42 | 0.52 | 0.52 | 0.00 |
| Task 1 | A2 | IPSGABA_OR_BoundarySeparation | 171 | -0.11 | 1.5E-01 | 0.06 | 3.2E-01 | 1.37 | 0.98 | 0.01 | 0.49 | 0.49 | 0.01 |
| Task 1 | A2 | IPSGABA_EX_BoundarySeparation | 172 | -0.03 | 7.4E-01 | 0.05 | 4.9E-01 | 1.31 | 0.99 | 0.31 | 0.51 | 0.51 | 0.00 |
| Task 1 | A2 | IPSNAA_overall_BoundarySeparation | 176 | 0.07 | 1.5E-01 | -0.09 | 2.2E-02 | 3.35 | 0.99 | 0.12 | 0.67 | 0.67 | 0.00 |
| Task 1 | A2 | IPSNAA_AL_BoundarySeparation | 172 | -0.07 | 2.7E-01 | 0.09 | 9.9E-02 | 1.29 | 0.99 | 0.70 | 0.53 | 0.53 | 0.00 |
| Task 1 | A2 | IPSNAA_OR_BoundarySeparation | 171 | -0.08 | 2.4E-01 | 0.02 | 6.7E-01 | 1.28 | 0.98 | 0.01 | 0.49 | 0.49 | 0.00 |
| Task 1 | A2 | IPSNAA_EX_BoundarySeparation | 171 | 0.01 | 9.2E-01 | -0.09 | 5.9E-02 | 1.27 | 0.99 | 0.54 | 0.53 | 0.54 | 0.00 |
| Task 1 | A2 | MFGGLU_overall_BoundarySeparation | 168 | -0.01 | 9.0E-01 | -0.12 | 1.2E-02 | 3.53 | 0.99 | 0.24 | 0.70 | 0.70 | 0.00 |
| Task 1 | A2 | MFGGLU_AL_BoundarySeparation | 166 | 0.07 | 2.5E-01 | -0.11 | 9.8E-02 | 1.46 | 0.99 | 0.42 | 0.56 | 0.56 | 0.00 |
| Task 1 | A2 | MFGGLU_OR_BoundarySeparation | 163 | 0.02 | 8.0E-01 | -0.11 | 8.7E-02 | 1.48 | 0.97 | 0.00 | 0.44 | 0.45 | 0.00 |
| Task 1 | A2 | MFGGLU_EX_BoundarySeparation | 166 | -0.01 | 9.0E-01 | -0.10 | 1.4E-01 | 1.48 | 0.99 | 0.46 | 0.51 | 0.51 | 0.00 |
| Task 1 | A2 | MFGGABA_overall_BoundarySeparation | 167 | -0.02 | 7.4E-01 | -0.05 | 2.7E-01 | 3.37 | 0.99 | 0.24 | 0.68 | 0.68 | 0.00 |
| Task 1 | A2 | MFGGABA_AL_BoundarySeparation | 165 | -0.04 | 5.6E-01 | 0.04 | 5.4E-01 | 1.30 | 0.99 | 0.27 | 0.55 | 0.55 | 0.00 |
| Task 1 | A2 | MFGGABA_OR_BoundarySeparation | 163 | 0.10 | 1.4E-01 | -0.16 | 1.8E-02 | 1.37 | 0.98 | 0.01 | 0.46 | 0.45 | 0.01 |
| Task 1 | A2 | MFGGABA_EX_BoundarySeparation | 164 | 0.04 | 5.2E-01 | -0.06 | 3.0E-01 | 1.30 | 0.99 | 0.74 | 0.53 | 0.53 | 0.00 |
| Task 1 | A2 | MFGNAA_overall_BoundarySeparation | 169 | 0.08 | 1.1E-01 | -0.15 | 2.3E-03 | 3.35 | 0.99 | 0.13 | 0.70 | 0.69 | 0.00 |
| Task 1 | A2 | MFGNAA_AL_BoundarySeparation | 166 | -0.06 | 3.6E-01 | 0.03 | 6.0E-01 | 1.37 | 0.99 | 0.58 | 0.55 | 0.55 | 0.00 |
| Task 1 | A2 | MFGNAA_OR_BoundarySeparation | 162 | -0.01 | 8.9E-01 | 0.01 | 8.7E-01 | 1.44 | 0.98 | 0.01 | 0.46 | 0.46 | 0.00 |
| Task 1 | A2 | MFGNAA_EX_BoundarySeparation | 165 | 0.13 | 1.8E-02 | -0.19 | 1.1E-03 | 1.37 | 0.99 | 0.29 | 0.56 | 0.55 | 0.01 |
| Task 1 | A2 | IPSGLU_overall_NonDecisionTime | 174 | -0.24 | 1.4E-04 | 0.05 | 4.1E-01 | 3.28 | 0.99 | 0.05 | 0.63 | 0.59 | 0.04 |
| Task 1 | A2 | IPSGLU_AL_NonDecisionTime | 171 | -0.19 | 1.0E-02 | 0.14 | 4.3E-02 | 1.65 | 0.98 | 0.04 | 0.42 | 0.40 | 0.03 |
| Task 1 | A2 | IPSGLU_OR_NonDecisionTime | 171 | -0.14 | 1.3E-01 | 0.07 | 4.1E-01 | 1.65 | 0.95 | 0.00 | 0.47 | 0.46 | 0.01 |
| Task 1 | A2 | IPSGLU_EX_NonDecisionTime | 170 | -0.24 | 2.9E-05 | 0.16 | 9.2E-03 | 1.70 | 0.99 | 0.07 | 0.59 | 0.54 | 0.04 |
| Task 1 | A2 | IPSGABA_overall_NonDecisionTime | 175 | 0.24 | 1.7E-03 | -0.11 | 5.7E-02 | 2.99 | 0.98 | 0.03 | 0.61 | 0.57 | 0.04 |
| Task 1 | A2 | IPSGABA_AL_NonDecisionTime | 172 | 0.13 | 9.3E-02 | -0.10 | 9.0E-02 | 1.65 | 0.98 | 0.02 | 0.41 | 0.40 | 0.01 |
| Task 1 | A2 | IPSGABA_OR_NonDecisionTime | 170 | 0.06 | 5.3E-01 | -0.01 | 8.7E-01 | 1.37 | 0.96 | 0.00 | 0.44 | 0.44 | 0.00 |
| Task 1 | A2 | IPSGABA_EX_NonDecisionTime | 170 | 0.04 | 6.3E-01 | -0.03 | 6.4E-01 | 1.31 | 0.98 | 0.02 | 0.51 | 0.51 | 0.00 |
| Task 1 | A2 | IPSNAA_overall_NonDecisionTime | 172 | 0.11 | 5.9E-02 | -0.18 | 2.3E-04 | 2.81 | 0.99 | 0.08 | 0.62 | 0.62 | 0.01 |
| Task 1 | A2 | IPSNAA_AL_NonDecisionTime | 172 | 0.05 | 5.2E-01 | 0.03 | 6.1E-01 | 1.65 | 0.98 | 0.01 | 0.39 | 0.39 | 0.00 |
| Task 1 | A2 | IPSNAA_OR_NonDecisionTime | 170 | -0.13 | 1.2E-01 | 0.05 | 4.8E-01 | 1.40 | 0.97 | 0.00 | 0.48 | 0.46 | 0.01 |
| Task 1 | A2 | IPSNAA_EX_NonDecisionTime | 171 | -0.07 | 3.4E-01 | 0.01 | 8.7E-01 | 1.32 | 0.98 | 0.02 | 0.51 | 0.51 | 0.00 |
| Task 1 | A2 | MFGGLU_overall_NonDecisionTime | 166 | -0.20 | 6.8E-04 | 0.12 | 3.7E-02 | 3.17 | 0.99 | 0.18 | 0.62 | 0.60 | 0.03 |
| Task 1 | A2 | MFGGLU_AL_NonDecisionTime | 165 | -0.16 | 3.3E-02 | 0.03 | 6.2E-01 | 1.69 | 0.98 | 0.01 | 0.41 | 0.39 | 0.02 |
| Task 1 | A2 | MFGGLU_OR_NonDecisionTime | 163 | -0.11 | 2.4E-01 | -0.01 | 9.2E-01 | 1.43 | 0.95 | 0.00 | 0.40 | 0.40 | 0.00 |
| Task 1 | A2 | MFGGLU_EX_NonDecisionTime | 163 | -0.15 | 4.7E-02 | 0.00 | 9.9E-01 | 1.50 | 0.98 | 0.01 | 0.52 | 0.51 | 0.01 |
| Task 1 | A2 | MFGGABA_overall_NonDecisionTime | 165 | 0.13 | 6.8E-03 | -0.08 | 1.1E-01 | 2.94 | 0.99 | 0.13 | 0.60 | 0.59 | 0.01 |
| Task 1 | A2 | MFGGABA_AL_NonDecisionTime | 165 | 0.07 | 2.6E-01 | -0.06 | 2.7E-01 | 1.68 | 0.98 | 0.01 | 0.42 | 0.42 | 0.00 |
| Task 1 | A2 | MFGGABA_OR_NonDecisionTime | 162 | 0.15 | 2.4E-02 | -0.08 | 2.5E-01 | 1.41 | 0.97 | 0.00 | 0.51 | 0.49 | 0.02 |
| Task 1 | A2 | MFGGABA_EX_NonDecisionTime | 162 | 0.04 | 6.3E-01 | 0.01 | 8.6E-01 | 1.37 | 0.98 | 0.01 | 0.51 | 0.51 | 0.00 |
| Task 1 | A2 | MFGNAA_overall_NonDecisionTime | 167 | 0.14 | 4.4E-03 | -0.14 | 7.6E-03 | 3.08 | 0.98 | 0.04 | 0.62 | 0.60 | 0.02 |
| Task 1 | A2 | MFGNAA_AL_NonDecisionTime | 165 | 0.06 | 3.2E-01 | -0.02 | 7.4E-01 | 1.69 | 0.97 | 0.00 | 0.39 | 0.39 | 0.00 |
| Task 1 | A2 | MFGNAA_OR_NonDecisionTime | 163 | 0.06 | 4.4E-01 | -0.04 | 6.6E-01 | 1.37 | 0.96 | 0.00 | 0.40 | 0.40 | 0.00 |
| Task 1 | A2 | MFGNAA_EX_NonDecisionTime | 163 | 0.19 | 2.4E-03 | -0.21 | 1.5E-03 | 1.41 | 0.99 | 0.20 | 0.58 | 0.55 | 0.03 |
| Task 2 | A2 | IPSGLU_overall_MeanDriftRate | 171 | -0.04 | 5.1E-01 | 0.00 | 9.9E-01 | 3.19 | 0.99 | 0.75 | 0.64 | 0.65 | 0.00 |
| Task 2 | A2 | IPSGLU_SNARC_MeanDriftRate | 175 | -0.11 | 1.6E-01 | 0.06 | 5.1E-01 | 2.02 | 0.99 | 0.53 | 0.07 | 0.06 | 0.00 |
| Task 2 | A2 | IPSGLU_DISTANCE_MeanDriftRate | 172 | 0.15 | 4.3E-02 | 0.00 | 1.0E+00 | 1.81 | 0.99 | 0.39 | 0.26 | 0.25 | 0.01 |
| Task 2 | A2 | IPSGABA_overall_MeanDriftRate | 171 | -0.13 | 1.5E-02 | 0.04 | 3.7E-01 | 3.41 | 0.99 | 0.68 | 0.65 | 0.65 | 0.01 |
| Task 2 | A2 | IPSGABA_SNARC_MeanDriftRate | 175 | 0.08 | 3.0E-01 | -0.14 | 5.5E-02 | 2.10 | 0.99 | 0.28 | 0.07 | 0.07 | 0.00 |
| Task 2 | A2 | IPSGABA_DISTANCE_MeanDriftRate | 173 | -0.16 | 1.7E-02 | 0.04 | 5.7E-01 | 1.68 | 0.99 | 0.27 | 0.27 | 0.25 | 0.02 |
| Task 2 | A2 | IPSNAA_overall_MeanDriftRate | 171 | -0.05 | 3.1E-01 | 0.01 | 8.9E-01 | 3.19 | 0.99 | 0.77 | 0.65 | 0.65 | 0.00 |
| Task 2 | A2 | IPSNAA_SNARC_MeanDriftRate | 175 | 0.06 | 3.5E-01 | 0.00 | 9.8E-01 | 2.01 | 0.99 | 0.12 | 0.06 | 0.06 | 0.00 |
| Task 2 | A2 | IPSNAA_DISTANCE_MeanDriftRate | 172 | 0.03 | 6.6E-01 | 0.23 | 2.3E-04 | 1.68 | 1.00 | 0.94 | 0.30 | 0.30 | 0.00 |
| Task 2 | A2 | MFGGLU_overall_MeanDriftRate | 164 | 0.05 | 3.4E-01 | -0.05 | 2.9E-01 | 3.03 | 0.99 | 0.80 | 0.64 | 0.64 | 0.00 |
| Task 2 | A2 | MFGGLU_SNARC_MeanDriftRate | 168 | 0.00 | 9.7E-01 | 0.05 | 5.3E-01 | 2.06 | 0.99 | 0.18 | 0.05 | 0.06 | -0.01 |
| Task 2 | A2 | MFGGLU_DISTANCE_MeanDriftRate | 166 | 0.06 | 4.9E-01 | -0.03 | 7.2E-01 | 1.80 | 0.99 | 0.18 | 0.24 | 0.25 | 0.00 |
| Task 2 | A2 | MFGGABA_overall_MeanDriftRate | 163 | -0.04 | 4.2E-01 | -0.05 | 3.5E-01 | 3.12 | 1.00 | 0.91 | 0.64 | 0.64 | 0.00 |
| Task 2 | A2 | MFGGABA_SNARC_MeanDriftRate | 167 | -0.01 | 8.5E-01 | -0.10 | 1.9E-01 | 1.99 | 0.99 | 0.34 | 0.07 | 0.07 | -0.01 |
| Task 2 | A2 | MFGGABA_DISTANCE_MeanDriftRate | 166 | -0.02 | 7.8E-01 | 0.04 | 5.9E-01 | 1.79 | 0.99 | 0.13 | 0.26 | 0.26 | 0.00 |
| Task 2 | A2 | MFGNAA_overall_MeanDriftRate | 164 | -0.01 | 8.9E-01 | -0.08 | 1.1E-01 | 3.22 | 0.99 | 0.80 | 0.64 | 0.64 | 0.00 |
| Task 2 | A2 | MFGNAA_SNARC_MeanDriftRate | 168 | 0.11 | 5.8E-02 | -0.07 | 3.7E-01 | 2.07 | 0.99 | 0.25 | 0.07 | 0.06 | 0.01 |
| Task 2 | A2 | MFGNAA_DISTANCE_MeanDriftRate | 166 | -0.18 | 5.9E-03 | -0.02 | 8.3E-01 | 1.80 | 0.99 | 0.32 | 0.30 | 0.27 | 0.03 |
| Task 2 | A2 | IPSGLU_overall_BoundarySeparation | 167 | -0.15 | 7.6E-03 | 0.03 | 6.5E-01 | 3.95 | 0.99 | 0.12 | 0.63 | 0.61 | 0.01 |
| Task 2 | A2 | IPSGLU_SNARC_BoundarySeparation | 174 | -0.05 | 4.5E-01 | -0.02 | 7.7E-01 | 1.67 | 1.00 | 0.88 | 0.56 | 0.56 | 0.00 |
| Task 2 | A2 | IPSGLU_DISTANCE_BoundarySeparation | 172 | 0.19 | 4.4E-03 | -0.05 | 5.0E-01 | 1.95 | 0.99 | 0.72 | 0.33 | 0.30 | 0.03 |
| Task 2 | A2 | IPSGABA_overall_BoundarySeparation | 168 | -0.06 | 4.4E-01 | -0.04 | 5.8E-01 | 3.81 | 0.98 | 0.01 | 0.59 | 0.59 | 0.00 |
| Task 2 | A2 | IPSGABA_SNARC_BoundarySeparation | 174 | 0.12 | 3.6E-02 | 0.06 | 2.9E-01 | 1.29 | 0.99 | 0.68 | 0.57 | 0.56 | 0.01 |
| Task 2 | A2 | IPSGABA_DISTANCE_BoundarySeparation | 173 | 0.09 | 2.2E-01 | -0.06 | 4.7E-01 | 1.69 | 1.00 | 0.85 | 0.31 | 0.31 | 0.00 |
| Task 2 | A2 | IPSNAA_overall_BoundarySeparation | 167 | -0.01 | 9.3E-01 | -0.06 | 1.6E-01 | 3.61 | 0.98 | 0.03 | 0.61 | 0.62 | 0.00 |
| Task 2 | A2 | IPSNAA_SNARC_BoundarySeparation | 174 | -0.04 | 4.9E-01 | 0.00 | 9.3E-01 | 1.14 | 0.99 | 0.77 | 0.56 | 0.56 | 0.00 |
| Task 2 | A2 | IPSNAA_DISTANCE_BoundarySeparation | 173 | -0.01 | 8.9E-01 | 0.10 | 1.2E-01 | 1.51 | 1.00 | 0.76 | 0.31 | 0.32 | 0.00 |
| Task 2 | A2 | MFGGLU_overall_BoundarySeparation | 161 | -0.11 | 9.8E-02 | -0.03 | 5.9E-01 | 3.77 | 0.98 | 0.01 | 0.64 | 0.64 | 0.01 |
| Task 2 | A2 | MFGGLU_SNARC_BoundarySeparation | 167 | -0.01 | 8.9E-01 | -0.04 | 5.0E-01 | 1.50 | 0.99 | 0.69 | 0.57 | 0.57 | 0.00 |
| Task 2 | A2 | MFGGLU_DISTANCE_BoundarySeparation | 166 | 0.17 | 1.5E-02 | -0.04 | 5.3E-01 | 1.83 | 0.99 | 0.45 | 0.35 | 0.33 | 0.02 |
| Task 2 | A2 | MFGGABA_overall_BoundarySeparation | 160 | -0.03 | 5.4E-01 | 0.03 | 6.1E-01 | 3.54 | 0.97 | 0.00 | 0.61 | 0.61 | 0.00 |
| Task 2 | A2 | MFGGABA_SNARC_BoundarySeparation | 166 | 0.07 | 1.5E-01 | 0.05 | 4.2E-01 | 1.23 | 1.00 | 0.85 | 0.55 | 0.55 | 0.00 |
| Task 2 | A2 | MFGGABA_DISTANCE_BoundarySeparation | 165 | 0.04 | 5.6E-01 | -0.03 | 6.8E-01 | 1.63 | 0.99 | 0.73 | 0.35 | 0.35 | 0.00 |
| Task 2 | A2 | MFGNAA_overall_BoundarySeparation | 162 | -0.02 | 7.1E-01 | -0.14 | 5.1E-03 | 3.48 | 0.97 | 0.00 | 0.60 | 0.60 | 0.00 |
| Task 2 | A2 | MFGNAA_SNARC_BoundarySeparation | 167 | 0.03 | 5.2E-01 | -0.01 | 8.5E-01 | 1.34 | 0.99 | 0.79 | 0.57 | 0.57 | 0.00 |
| Task 2 | A2 | MFGNAA_DISTANCE_BoundarySeparation | 166 | 0.08 | 2.4E-01 | 0.18 | 4.9E-03 | 1.69 | 0.99 | 0.39 | 0.37 | 0.36 | 0.00 |
| Task 2 | A2 | IPSGLU_overall_NonDecisionTime | 170 | -0.16 | 1.7E-02 | 0.06 | 2.9E-01 | 3.32 | 0.99 | 0.10 | 0.68 | 0.67 | 0.02 |
| Task 2 | A2 | IPSGLU_SNARC_NonDecisionTime | 174 | -0.02 | 7.0E-01 | 0.01 | 8.5E-01 | 1.68 | 0.99 | 0.23 | 0.53 | 0.53 | 0.00 |
| Task 2 | A2 | IPSGLU_DISTANCE_NonDecisionTime | 171 | 0.25 | 9.4E-04 | -0.07 | 2.8E-01 | 1.84 | 0.98 | 0.01 | 0.49 | 0.44 | 0.05 |
| Task 2 | A2 | IPSGABA_overall_NonDecisionTime | 168 | 0.29 | 5.8E-07 | -0.07 | 1.9E-01 | 2.92 | 0.98 | 0.03 | 0.73 | 0.67 | 0.06 |
| Task 2 | A2 | IPSGABA_SNARC_NonDecisionTime | 174 | -0.03 | 6.3E-01 | 0.06 | 2.8E-01 | 1.31 | 0.99 | 0.18 | 0.53 | 0.54 | 0.00 |
| Task 2 | A2 | IPSGABA_DISTANCE_NonDecisionTime | 170 | -0.01 | 9.5E-01 | 0.00 | 9.9E-01 | 1.53 | 0.97 | 0.00 | 0.44 | 0.45 | 0.00 |
| Task 2 | A2 | IPSNAA_overall_NonDecisionTime | 168 | 0.12 | 7.4E-03 | -0.14 | 1.5E-04 | 2.83 | 0.97 | 0.00 | 0.70 | 0.69 | 0.01 |
| Task 2 | A2 | IPSNAA_SNARC_NonDecisionTime | 174 | -0.04 | 5.5E-01 | 0.03 | 6.3E-01 | 1.17 | 0.99 | 0.21 | 0.53 | 0.53 | 0.00 |
| Task 2 | A2 | IPSNAA_DISTANCE_NonDecisionTime | 171 | 0.00 | 9.6E-01 | 0.06 | 2.7E-01 | 1.41 | 0.96 | 0.00 | 0.44 | 0.44 | 0.00 |
| Task 2 | A2 | MFGGLU_overall_NonDecisionTime | 162 | -0.12 | 7.6E-02 | 0.01 | 9.2E-01 | 2.97 | 0.98 | 0.01 | 0.66 | 0.65 | 0.01 |
| Task 2 | A2 | MFGGLU_SNARC_NonDecisionTime | 167 | -0.06 | 3.5E-01 | 0.02 | 6.8E-01 | 1.52 | 0.99 | 0.10 | 0.55 | 0.55 | 0.00 |
| Task 2 | A2 | MFGGLU_DISTANCE_NonDecisionTime | 163 | 0.07 | 4.0E-01 | -0.01 | 9.3E-01 | 1.69 | 0.97 | 0.00 | 0.48 | 0.48 | 0.00 |
| Task 2 | A2 | MFGGABA_overall_NonDecisionTime | 161 | 0.17 | 5.0E-04 | -0.11 | 3.9E-03 | 2.97 | 0.97 | 0.00 | 0.71 | 0.68 | 0.02 |
| Task 2 | A2 | MFGGABA_SNARC_NonDecisionTime | 166 | -0.03 | 6.4E-01 | 0.04 | 4.9E-01 | 1.24 | 0.99 | 0.12 | 0.53 | 0.53 | 0.00 |
| Task 2 | A2 | MFGGABA_DISTANCE_NonDecisionTime | 163 | -0.04 | 5.1E-01 | 0.02 | 7.0E-01 | 1.50 | 0.97 | 0.00 | 0.48 | 0.48 | 0.00 |
| Task 2 | A2 | MFGNAA_overall_NonDecisionTime | 162 | 0.18 | 1.6E-04 | -0.11 | 1.9E-02 | 2.87 | 0.97 | 0.00 | 0.70 | 0.67 | 0.03 |
| Task 2 | A2 | MFGNAA_SNARC_NonDecisionTime | 167 | -0.01 | 8.4E-01 | 0.02 | 7.6E-01 | 1.35 | 0.99 | 0.08 | 0.54 | 0.55 | 0.00 |
| Task 2 | A2 | MFGNAA_DISTANCE_NonDecisionTime | 164 | -0.06 | 3.1E-01 | 0.17 | 3.4E-03 | 1.60 | 0.97 | 0.00 | 0.52 | 0.52 | 0.00 |
| Task 3 | A2 | IPSGLU_overall_MeanDriftRate | 168 | 0.02 | 7.3E-01 | 0.00 | 9.6E-01 | 2.80 | 0.99 | 0.43 | 0.64 | 0.64 | 0.00 |
| Task 3 | A2 | IPSGLU_DISTANCE_MeanDriftRate | 170 | -0.09 | 2.6E-01 | 0.09 | 2.9E-01 | 2.71 | 0.99 | 0.29 | 0.08 | 0.08 | 0.00 |
| Task 3 | A2 | IPSGABA_overall_MeanDriftRate | 168 | -0.12 | 3.3E-02 | 0.00 | 9.7E-01 | 2.32 | 0.99 | 0.49 | 0.65 | 0.64 | 0.01 |
| Task 3 | A2 | IPSGABA_DISTANCE_MeanDriftRate | 169 | 0.00 | 9.6E-01 | -0.03 | 7.6E-01 | 2.77 | 0.99 | 0.26 | 0.07 | 0.07 | -0.01 |
| Task 3 | A2 | IPSNAA_overall_MeanDriftRate | 168 | -0.06 | 3.1E-01 | 0.00 | 9.6E-01 | 2.29 | 0.99 | 0.43 | 0.64 | 0.64 | 0.00 |
| Task 3 | A2 | IPSNAA_DISTANCE_MeanDriftRate | 169 | -0.03 | 7.3E-01 | -0.06 | 4.3E-01 | 2.71 | 0.99 | 0.20 | 0.07 | 0.07 | 0.00 |
| Task 3 | A2 | MFGGLU_overall_MeanDriftRate | 164 | 0.06 | 2.9E-01 | -0.08 | 1.3E-01 | 2.54 | 0.99 | 0.83 | 0.65 | 0.65 | 0.00 |
| Task 3 | A2 | MFGGLU_DISTANCE_MeanDriftRate | 165 | -0.05 | 5.8E-01 | 0.00 | 9.7E-01 | 2.82 | 0.99 | 0.29 | 0.07 | 0.07 | 0.00 |
| Task 3 | A2 | MFGGABA_overall_MeanDriftRate | 162 | -0.03 | 4.8E-01 | 0.01 | 8.8E-01 | 2.46 | 0.99 | 0.37 | 0.65 | 0.65 | 0.00 |
| Task 3 | A2 | MFGGABA_DISTANCE_MeanDriftRate | 163 | -0.08 | 2.9E-01 | 0.07 | 3.9E-01 | 2.70 | 0.99 | 0.34 | 0.09 | 0.09 | 0.00 |
| Task 3 | A2 | MFGNAA_overall_MeanDriftRate | 164 | -0.04 | 3.8E-01 | -0.04 | 4.8E-01 | 2.40 | 0.99 | 0.67 | 0.65 | 0.65 | 0.00 |
| Task 3 | A2 | MFGNAA_DISTANCE_MeanDriftRate | 165 | 0.03 | 6.9E-01 | -0.01 | 9.1E-01 | 2.83 | 0.99 | 0.16 | 0.07 | 0.07 | 0.00 |
| Task 3 | A2 | IPSGLU_overall_BoundarySeparation | 167 | -0.15 | 5.3E-02 | 0.06 | 3.3E-01 | 3.27 | 0.99 | 0.23 | 0.57 | 0.55 | 0.02 |
| Task 3 | A2 | IPSGLU_DISTANCE_BoundarySeparation | 169 | -0.01 | 9.0E-01 | 0.05 | 3.4E-01 | 1.80 | 0.98 | 0.04 | 0.69 | 0.70 | 0.00 |
| Task 3 | A2 | IPSGABA_overall_BoundarySeparation | 167 | 0.23 | 3.0E-04 | -0.09 | 8.6E-02 | 2.75 | 0.99 | 0.12 | 0.59 | 0.55 | 0.03 |
| Task 3 | A2 | IPSGABA_DISTANCE_BoundarySeparation | 169 | 0.07 | 2.7E-01 | -0.01 | 8.1E-01 | 1.40 | 0.99 | 0.11 | 0.70 | 0.69 | 0.00 |
| Task 3 | A2 | IPSNAA_overall_BoundarySeparation | 167 | 0.03 | 6.3E-01 | -0.15 | 4.8E-03 | 2.64 | 0.99 | 0.48 | 0.57 | 0.57 | 0.00 |
| Task 3 | A2 | IPSNAA_DISTANCE_BoundarySeparation | 168 | 0.07 | 1.5E-01 | -0.05 | 2.5E-01 | 1.24 | 0.99 | 0.26 | 0.70 | 0.69 | 0.00 |
| Task 3 | A2 | MFGGLU_overall_BoundarySeparation | 163 | -0.08 | 2.4E-01 | 0.00 | 9.9E-01 | 2.80 | 0.99 | 0.15 | 0.57 | 0.56 | 0.00 |
| Task 3 | A2 | MFGGLU_DISTANCE_BoundarySeparation | 163 | 0.01 | 9.2E-01 | -0.06 | 1.8E-01 | 1.60 | 0.99 | 0.15 | 0.72 | 0.72 | 0.00 |
| Task 3 | A2 | MFGGABA_overall_BoundarySeparation | 161 | 0.06 | 2.9E-01 | -0.02 | 7.5E-01 | 2.88 | 0.99 | 0.14 | 0.55 | 0.55 | 0.00 |
| Task 3 | A2 | MFGGABA_DISTANCE_BoundarySeparation | 163 | 0.02 | 7.0E-01 | 0.00 | 9.4E-01 | 1.36 | 0.98 | 0.03 | 0.69 | 0.69 | 0.00 |
| Task 3 | A2 | MFGNAA_overall_BoundarySeparation | 163 | 0.05 | 3.6E-01 | -0.10 | 8.2E-02 | 2.83 | 0.99 | 0.14 | 0.57 | 0.57 | 0.00 |
| Task 3 | A2 | MFGNAA_DISTANCE_BoundarySeparation | 164 | 0.03 | 4.8E-01 | -0.02 | 6.4E-01 | 1.41 | 0.99 | 0.19 | 0.72 | 0.72 | 0.00 |
| Task 3 | A2 | IPSGLU_overall_NonDecisionTime | 164 | 0.11 | 2.1E-01 | -0.10 | 1.8E-01 | 2.64 | 0.95 | 0.00 | 0.36 | 0.35 | 0.01 |
| Task 3 | A2 | IPSGLU_DISTANCE_NonDecisionTime | 167 | -0.01 | 9.3E-01 | 0.05 | 3.1E-01 | 1.76 | 0.97 | 0.00 | 0.69 | 0.69 | 0.00 |
| Task 3 | A2 | IPSGABA_overall_NonDecisionTime | 166 | 0.39 | 6.6E-06 | -0.22 | 7.7E-05 | 2.80 | 0.94 | 0.00 | 0.47 | 0.38 | 0.09 |
| Task 3 | A2 | IPSGABA_DISTANCE_NonDecisionTime | 167 | 0.09 | 2.5E-01 | 0.04 | 4.1E-01 | 1.36 | 0.97 | 0.00 | 0.69 | 0.69 | 0.00 |
| Task 3 | A2 | IPSNAA_overall_NonDecisionTime | 165 | 0.18 | 9.5E-03 | -0.20 | 1.4E-04 | 2.73 | 0.93 | 0.00 | 0.45 | 0.42 | 0.02 |
| Task 3 | A2 | IPSNAA_DISTANCE_NonDecisionTime | 167 | 0.12 | 6.5E-02 | -0.04 | 3.8E-01 | 1.21 | 0.96 | 0.00 | 0.70 | 0.69 | 0.01 |
| Task 3 | A2 | MFGGLU_overall_NonDecisionTime | 161 | -0.18 | 2.7E-02 | 0.04 | 5.2E-01 | 2.79 | 0.93 | 0.00 | 0.37 | 0.35 | 0.02 |
| Task 3 | A2 | MFGGLU_DISTANCE_NonDecisionTime | 163 | 0.01 | 9.2E-01 | -0.05 | 2.8E-01 | 1.55 | 0.97 | 0.00 | 0.69 | 0.69 | 0.00 |
| Task 3 | A2 | MFGGABA_overall_NonDecisionTime | 160 | 0.06 | 4.9E-01 | -0.02 | 7.6E-01 | 2.78 | 0.93 | 0.00 | 0.39 | 0.39 | 0.00 |
| Task 3 | A2 | MFGGABA_DISTANCE_NonDecisionTime | 161 | 0.04 | 4.1E-01 | -0.02 | 6.7E-01 | 1.33 | 0.97 | 0.00 | 0.69 | 0.69 | 0.00 |
| Task 3 | A2 | MFGNAA_overall_NonDecisionTime | 162 | 0.02 | 8.7E-01 | -0.08 | 3.3E-01 | 2.78 | 0.92 | 0.00 | 0.36 | 0.36 | 0.00 |
| Task 3 | A2 | MFGNAA_DISTANCE_NonDecisionTime | 163 | 0.11 | 4.6E-02 | -0.09 | 4.8E-02 | 1.40 | 0.97 | 0.00 | 0.71 | 0.70 | 0.01 |
| Task 1 | Prediction | IPSGLU_overall_MeanDriftRate | 161 | -0.08 | 1.4E-01 | -0.10 | 1.5E-01 | 4.79 | 0.99 | 0.44 | 0.67 | 0.67 | 0.00 |
| Task 1 | Prediction | IPSGLU_AL_MeanDriftRate | 160 | 0.09 | 2.6E-01 | 0.08 | 3.8E-01 | 1.65 | 0.99 | 0.08 | 0.35 | 0.35 | 0.00 |
| Task 1 | Prediction | IPSGLU_OR_MeanDriftRate | 156 | -0.02 | 7.1E-01 | -0.03 | 6.8E-01 | 1.78 | 0.97 | 0.00 | 0.41 | 0.41 | 0.00 |
| Task 1 | Prediction | IPSGLU_EX_MeanDriftRate | 159 | -0.12 | 1.2E-01 | -0.14 | 1.9E-01 | 2.10 | 0.98 | 0.00 | 0.23 | 0.23 | 0.01 |
| Task 1 | Prediction | IPSGABA_overall_MeanDriftRate | 161 | -0.13 | 2.3E-02 | 0.02 | 7.3E-01 | 4.79 | 1.00 | 0.87 | 0.67 | 0.66 | 0.01 |
| Task 1 | Prediction | IPSGABA_AL_MeanDriftRate | 160 | 0.06 | 4.1E-01 | 0.01 | 9.2E-01 | 1.53 | 0.99 | 0.26 | 0.37 | 0.37 | 0.00 |
| Task 1 | Prediction | IPSGABA_OR_MeanDriftRate | 156 | -0.01 | 9.1E-01 | -0.09 | 2.7E-01 | 1.84 | 0.97 | 0.00 | 0.43 | 0.43 | 0.00 |
| Task 1 | Prediction | IPSGABA_EX_MeanDriftRate | 159 | -0.03 | 7.5E-01 | 0.02 | 7.7E-01 | 2.17 | 0.97 | 0.00 | 0.21 | 0.21 | 0.00 |
| Task 1 | Prediction | IPSNAA_overall_MeanDriftRate | 162 | -0.03 | 6.2E-01 | -0.08 | 1.2E-01 | 5.33 | 0.99 | 0.48 | 0.67 | 0.67 | 0.00 |
| Task 1 | Prediction | IPSNAA_AL_MeanDriftRate | 161 | 0.02 | 8.2E-01 | 0.06 | 4.8E-01 | 1.58 | 0.99 | 0.11 | 0.34 | 0.35 | 0.00 |
| Task 1 | Prediction | IPSNAA_OR_MeanDriftRate | 157 | 0.03 | 7.0E-01 | -0.07 | 3.0E-01 | 1.79 | 0.97 | 0.00 | 0.41 | 0.42 | 0.00 |
| Task 1 | Prediction | IPSNAA_EX_MeanDriftRate | 160 | -0.06 | 4.2E-01 | -0.16 | 5.0E-02 | 2.11 | 0.97 | 0.00 | 0.23 | 0.23 | 0.00 |
| Task 1 | Prediction | MFGGLU_overall_MeanDriftRate | 159 | 0.01 | 8.3E-01 | -0.01 | 9.2E-01 | 5.00 | 0.99 | 0.25 | 0.69 | 0.69 | 0.00 |
| Task 1 | Prediction | MFGGLU_AL_MeanDriftRate | 158 | -0.15 | 4.7E-02 | -0.12 | 6.7E-02 | 1.55 | 0.99 | 0.25 | 0.44 | 0.43 | 0.01 |
| Task 1 | Prediction | MFGGLU_OR_MeanDriftRate | 155 | 0.03 | 6.9E-01 | 0.08 | 2.2E-01 | 1.81 | 0.97 | 0.01 | 0.45 | 0.45 | 0.00 |
| Task 1 | Prediction | MFGGLU_EX_MeanDriftRate | 158 | -0.09 | 2.6E-01 | -0.08 | 3.1E-01 | 2.05 | 0.97 | 0.00 | 0.24 | 0.24 | 0.00 |
| Task 1 | Prediction | MFGGABA_overall_MeanDriftRate | 154 | 0.13 | 6.3E-03 | 0.02 | 6.1E-01 | 5.15 | 0.98 | 0.02 | 0.70 | 0.69 | 0.01 |
| Task 1 | Prediction | MFGGABA_AL_MeanDriftRate | 154 | 0.04 | 5.0E-01 | 0.00 | 9.8E-01 | 1.47 | 0.99 | 0.21 | 0.38 | 0.38 | 0.00 |
| Task 1 | Prediction | MFGGABA_OR_MeanDriftRate | 151 | 0.19 | 3.0E-02 | -0.09 | 2.3E-01 | 1.77 | 0.97 | 0.00 | 0.41 | 0.38 | 0.03 |
| Task 1 | Prediction | MFGGABA_EX_MeanDriftRate | 153 | 0.11 | 1.6E-01 | 0.03 | 7.3E-01 | 2.06 | 0.97 | 0.00 | 0.22 | 0.22 | 0.00 |
| Task 1 | Prediction | MFGNAA_overall_MeanDriftRate | 159 | -0.03 | 6.6E-01 | -0.06 | 3.1E-01 | 6.73 | 0.99 | 0.15 | 0.67 | 0.67 | 0.00 |
| Task 1 | Prediction | MFGNAA_AL_MeanDriftRate | 158 | -0.04 | 5.8E-01 | -0.15 | 4.3E-02 | 1.61 | 0.99 | 0.66 | 0.39 | 0.40 | 0.00 |
| Task 1 | Prediction | MFGNAA_OR_MeanDriftRate | 156 | -0.06 | 5.2E-01 | 0.10 | 2.5E-01 | 1.78 | 0.96 | 0.00 | 0.38 | 0.38 | 0.00 |
| Task 1 | Prediction | MFGNAA_EX_MeanDriftRate | 157 | -0.02 | 7.7E-01 | -0.13 | 1.4E-01 | 2.17 | 0.97 | 0.00 | 0.23 | 0.24 | 0.00 |
| Task 1 | Prediction | IPSGLU_overall_BoundarySeparation | 161 | -0.11 | 3.1E-02 | -0.05 | 4.3E-01 | 4.56 | 0.99 | 0.23 | 0.71 | 0.70 | 0.01 |
| Task 1 | Prediction | IPSGLU_AL_BoundarySeparation | 158 | 0.08 | 3.1E-01 | 0.11 | 1.6E-01 | 1.61 | 0.99 | 0.27 | 0.53 | 0.52 | 0.00 |
| Task 1 | Prediction | IPSGLU_OR_BoundarySeparation | 156 | 0.01 | 8.3E-01 | -0.08 | 2.1E-01 | 1.77 | 0.98 | 0.02 | 0.54 | 0.54 | 0.00 |
| Task 1 | Prediction | IPSGLU_EX_BoundarySeparation | 157 | -0.05 | 5.3E-01 | -0.05 | 5.0E-01 | 1.62 | 0.99 | 0.48 | 0.55 | 0.55 | 0.00 |
| Task 1 | Prediction | IPSGABA_overall_BoundarySeparation | 161 | -0.10 | 6.1E-02 | 0.01 | 8.6E-01 | 4.38 | 0.99 | 0.18 | 0.69 | 0.69 | 0.00 |
| Task 1 | Prediction | IPSGABA_AL_BoundarySeparation | 159 | 0.00 | 9.7E-01 | -0.06 | 4.3E-01 | 1.39 | 0.99 | 0.19 | 0.52 | 0.52 | 0.00 |
| Task 1 | Prediction | IPSGABA_OR_BoundarySeparation | 157 | 0.12 | 4.3E-02 | -0.14 | 2.8E-02 | 1.56 | 0.98 | 0.01 | 0.51 | 0.50 | 0.01 |
| Task 1 | Prediction | IPSGABA_EX_BoundarySeparation | 158 | 0.03 | 6.3E-01 | -0.04 | 5.9E-01 | 1.47 | 0.99 | 0.57 | 0.55 | 0.55 | 0.00 |
| Task 1 | Prediction | IPSNAA_overall_BoundarySeparation | 162 | 0.01 | 8.5E-01 | -0.09 | 1.2E-01 | 5.00 | 0.99 | 0.18 | 0.70 | 0.70 | 0.00 |
| Task 1 | Prediction | IPSNAA_AL_BoundarySeparation | 159 | -0.05 | 4.6E-01 | 0.16 | 2.7E-02 | 1.41 | 0.99 | 0.69 | 0.54 | 0.54 | 0.00 |
| Task 1 | Prediction | IPSNAA_OR_BoundarySeparation | 157 | -0.02 | 8.1E-01 | -0.03 | 6.6E-01 | 1.56 | 0.98 | 0.03 | 0.54 | 0.54 | 0.00 |
| Task 1 | Prediction | IPSNAA_EX_BoundarySeparation | 158 | 0.19 | 5.4E-03 | -0.14 | 3.8E-02 | 1.47 | 0.99 | 0.38 | 0.58 | 0.56 | 0.03 |
| Task 1 | Prediction | MFGGLU_overall_BoundarySeparation | 160 | -0.07 | 2.7E-01 | -0.05 | 4.0E-01 | 4.70 | 0.99 | 0.07 | 0.71 | 0.71 | 0.00 |
| Task 1 | Prediction | MFGGLU_AL_BoundarySeparation | 157 | 0.11 | 1.5E-01 | -0.01 | 9.1E-01 | 1.41 | 0.99 | 0.36 | 0.57 | 0.56 | 0.00 |
| Task 1 | Prediction | MFGGLU_OR_BoundarySeparation | 155 | -0.01 | 8.6E-01 | 0.06 | 2.9E-01 | 1.65 | 0.98 | 0.02 | 0.56 | 0.57 | 0.00 |
| Task 1 | Prediction | MFGGLU_EX_BoundarySeparation | 157 | 0.07 | 4.0E-01 | -0.13 | 4.9E-02 | 1.44 | 0.99 | 0.26 | 0.54 | 0.54 | 0.00 |
| Task 1 | Prediction | MFGGABA_overall_BoundarySeparation | 155 | -0.08 | 8.6E-02 | 0.07 | 1.1E-01 | 4.70 | 0.99 | 0.30 | 0.71 | 0.71 | 0.00 |
| Task 1 | Prediction | MFGGABA_AL_BoundarySeparation | 152 | -0.02 | 8.0E-01 | 0.05 | 3.8E-01 | 1.23 | 0.99 | 0.40 | 0.53 | 0.54 | 0.00 |
| Task 1 | Prediction | MFGGABA_OR_BoundarySeparation | 151 | 0.15 | 1.8E-02 | -0.12 | 3.8E-02 | 1.38 | 0.98 | 0.02 | 0.57 | 0.55 | 0.01 |
| Task 1 | Prediction | MFGGABA_EX_BoundarySeparation | 150 | 0.12 | 5.0E-02 | -0.07 | 1.8E-01 | 1.33 | 0.99 | 0.38 | 0.58 | 0.57 | 0.01 |
| Task 1 | Prediction | MFGNAA_overall_BoundarySeparation | 158 | -0.02 | 7.8E-01 | -0.02 | 7.1E-01 | 6.32 | 0.99 | 0.30 | 0.72 | 0.72 | 0.00 |
| Task 1 | Prediction | MFGNAA_AL_BoundarySeparation | 156 | -0.01 | 8.4E-01 | -0.01 | 9.3E-01 | 1.53 | 0.99 | 0.41 | 0.54 | 0.54 | 0.00 |
| Task 1 | Prediction | MFGNAA_OR_BoundarySeparation | 156 | -0.02 | 7.8E-01 | 0.13 | 5.7E-02 | 1.56 | 0.98 | 0.02 | 0.54 | 0.54 | 0.00 |
| Task 1 | Prediction | MFGNAA_EX_BoundarySeparation | 156 | 0.19 | 1.6E-03 | -0.14 | 2.5E-02 | 1.52 | 0.99 | 0.53 | 0.57 | 0.55 | 0.03 |
| Task 1 | Prediction | IPSGLU_overall_NonDecisionTime | 159 | -0.16 | 2.5E-02 | 0.06 | 4.9E-01 | 4.03 | 0.98 | 0.01 | 0.57 | 0.56 | 0.02 |
| Task 1 | Prediction | IPSGLU_AL_NonDecisionTime | 155 | -0.28 | 2.2E-04 | 0.20 | 5.9E-03 | 1.76 | 0.99 | 0.46 | 0.44 | 0.38 | 0.06 |
| Task 1 | Prediction | IPSGLU_OR_NonDecisionTime | 155 | -0.01 | 9.1E-01 | -0.13 | 7.2E-02 | 1.51 | 0.97 | 0.00 | 0.39 | 0.39 | 0.00 |
| Task 1 | Prediction | IPSGLU_EX_NonDecisionTime | 155 | -0.12 | 4.2E-02 | 0.19 | 8.4E-03 | 1.74 | 0.98 | 0.03 | 0.54 | 0.53 | 0.01 |
| Task 1 | Prediction | IPSGABA_overall_NonDecisionTime | 159 | 0.22 | 5.3E-04 | -0.06 | 3.0E-01 | 4.03 | 0.98 | 0.04 | 0.56 | 0.52 | 0.04 |
| Task 1 | Prediction | IPSGABA_AL_NonDecisionTime | 155 | 0.10 | 2.0E-01 | 0.01 | 9.4E-01 | 1.69 | 1.00 | 0.84 | 0.38 | 0.38 | 0.00 |
| Task 1 | Prediction | IPSGABA_OR_NonDecisionTime | 155 | 0.13 | 7.6E-02 | -0.03 | 6.9E-01 | 1.52 | 0.98 | 0.04 | 0.42 | 0.41 | 0.01 |
| Task 1 | Prediction | IPSGABA_EX_NonDecisionTime | 155 | 0.00 | 9.8E-01 | 0.04 | 5.3E-01 | 1.37 | 0.99 | 0.37 | 0.54 | 0.54 | 0.00 |
| Task 1 | Prediction | IPSNAA_overall_NonDecisionTime | 160 | 0.17 | 8.0E-03 | -0.16 | 4.9E-03 | 3.91 | 0.98 | 0.01 | 0.57 | 0.54 | 0.02 |
| Task 1 | Prediction | IPSNAA_AL_NonDecisionTime | 156 | -0.01 | 8.9E-01 | 0.14 | 7.4E-02 | 1.70 | 0.99 | 0.16 | 0.39 | 0.39 | 0.00 |
| Task 1 | Prediction | IPSNAA_OR_NonDecisionTime | 156 | 0.19 | 1.5E-02 | -0.19 | 9.0E-03 | 1.41 | 0.98 | 0.02 | 0.48 | 0.46 | 0.03 |
| Task 1 | Prediction | IPSNAA_EX_NonDecisionTime | 156 | 0.19 | 1.1E-02 | -0.05 | 5.3E-01 | 1.41 | 0.99 | 0.07 | 0.54 | 0.52 | 0.03 |
| Task 1 | Prediction | MFGGLU_overall_NonDecisionTime | 159 | -0.08 | 2.5E-01 | 0.03 | 6.4E-01 | 4.04 | 0.98 | 0.01 | 0.56 | 0.56 | 0.00 |
| Task 1 | Prediction | MFGGLU_AL_NonDecisionTime | 153 | -0.09 | 3.0E-01 | 0.10 | 2.0E-01 | 1.91 | 1.00 | 0.95 | 0.37 | 0.37 | 0.00 |
| Task 1 | Prediction | MFGGLU_OR_NonDecisionTime | 154 | -0.14 | 6.2E-02 | -0.03 | 7.5E-01 | 1.48 | 0.98 | 0.01 | 0.49 | 0.48 | 0.01 |
| Task 1 | Prediction | MFGGLU_EX_NonDecisionTime | 154 | -0.04 | 6.1E-01 | 0.04 | 6.1E-01 | 1.56 | 0.98 | 0.06 | 0.52 | 0.52 | 0.00 |
| Task 1 | Prediction | MFGGABA_overall_NonDecisionTime | 154 | 0.14 | 2.2E-02 | -0.12 | 1.4E-02 | 4.14 | 0.98 | 0.05 | 0.58 | 0.57 | 0.01 |
| Task 1 | Prediction | MFGGABA_AL_NonDecisionTime | 149 | -0.02 | 8.3E-01 | 0.06 | 4.4E-01 | 1.81 | 0.99 | 0.13 | 0.34 | 0.34 | 0.00 |
| Task 1 | Prediction | MFGGABA_OR_NonDecisionTime | 149 | 0.09 | 2.6E-01 | -0.07 | 2.4E-01 | 1.54 | 0.97 | 0.00 | 0.44 | 0.43 | 0.00 |
| Task 1 | Prediction | MFGGABA_EX_NonDecisionTime | 148 | -0.01 | 8.7E-01 | -0.01 | 9.4E-01 | 1.37 | 0.99 | 0.50 | 0.55 | 0.56 | 0.00 |
| Task 1 | Prediction | MFGNAA_overall_NonDecisionTime | 158 | 0.20 | 4.1E-03 | -0.07 | 3.1E-01 | 4.02 | 0.98 | 0.01 | 0.60 | 0.57 | 0.03 |
| Task 1 | Prediction | MFGNAA_AL_NonDecisionTime | 152 | 0.04 | 6.6E-01 | 0.07 | 3.7E-01 | 1.78 | 1.00 | 0.97 | 0.36 | 0.36 | 0.00 |
| Task 1 | Prediction | MFGNAA_OR_NonDecisionTime | 154 | 0.19 | 3.0E-02 | -0.12 | 1.4E-01 | 1.53 | 0.99 | 0.15 | 0.52 | 0.49 | 0.03 |
| Task 1 | Prediction | MFGNAA_EX_NonDecisionTime | 154 | 0.20 | 5.3E-03 | -0.07 | 3.3E-01 | 1.49 | 0.98 | 0.03 | 0.53 | 0.50 | 0.03 |
| Task 2 | Prediction | IPSGLU_overall_MeanDriftRate | 149 | 0.00 | 1.0E+00 | 0.04 | 5.5E-01 | 5.55 | 1.00 | 0.92 | 0.65 | 0.65 | 0.00 |
| Task 2 | Prediction | IPSGLU_SNARC_MeanDriftRate | 152 | 0.01 | 9.4E-01 | 0.10 | 3.1E-01 | 2.16 | 0.99 | 0.32 | 0.10 | 0.11 | -0.01 |
| Task 2 | Prediction | IPSGLU_DISTANCE_MeanDriftRate | 153 | 0.16 | 4.1E-02 | -0.01 | 8.8E-01 | 1.84 | 0.99 | 0.13 | 0.27 | 0.26 | 0.01 |
| Task 2 | Prediction | IPSGABA_overall_MeanDriftRate | 149 | -0.14 | 1.5E-02 | 0.02 | 7.6E-01 | 5.84 | 1.00 | 0.90 | 0.66 | 0.65 | 0.01 |
| Task 2 | Prediction | IPSGABA_SNARC_MeanDriftRate | 152 | 0.18 | 1.7E-02 | 0.02 | 8.3E-01 | 2.17 | 0.99 | 0.17 | 0.12 | 0.10 | 0.02 |
| Task 2 | Prediction | IPSGABA_DISTANCE_MeanDriftRate | 153 | -0.09 | 2.6E-01 | -0.10 | 3.2E-01 | 1.71 | 0.99 | 0.15 | 0.23 | 0.23 | 0.00 |
| Task 2 | Prediction | IPSNAA_overall_MeanDriftRate | 150 | 0.00 | 9.9E-01 | -0.07 | 2.3E-01 | 5.61 | 1.00 | 0.90 | 0.65 | 0.65 | 0.00 |
| Task 2 | Prediction | IPSNAA_SNARC_MeanDriftRate | 153 | 0.12 | 7.4E-02 | 0.08 | 3.3E-01 | 2.18 | 0.99 | 0.59 | 0.11 | 0.10 | 0.01 |
| Task 2 | Prediction | IPSNAA_DISTANCE_MeanDriftRate | 153 | -0.18 | 2.3E-02 | 0.08 | 3.6E-01 | 1.76 | 0.99 | 0.18 | 0.28 | 0.26 | 0.02 |
| Task 2 | Prediction | MFGGLU_overall_MeanDriftRate | 148 | 0.03 | 6.5E-01 | 0.00 | 9.5E-01 | 5.68 | 0.99 | 0.70 | 0.64 | 0.64 | 0.00 |
| Task 2 | Prediction | MFGGLU_SNARC_MeanDriftRate | 151 | -0.08 | 3.5E-01 | -0.11 | 2.3E-01 | 2.15 | 0.99 | 0.83 | 0.13 | 0.13 | 0.00 |
| Task 2 | Prediction | MFGGLU_DISTANCE_MeanDriftRate | 153 | 0.12 | 2.3E-01 | -0.09 | 3.4E-01 | 1.87 | 0.99 | 0.09 | 0.25 | 0.25 | 0.00 |
| Task 2 | Prediction | MFGGABA_overall_MeanDriftRate | 144 | 0.11 | 4.7E-02 | -0.04 | 4.8E-01 | 6.17 | 0.99 | 0.63 | 0.66 | 0.65 | 0.01 |
| Task 2 | Prediction | MFGGABA_SNARC_MeanDriftRate | 145 | 0.09 | 3.4E-01 | -0.14 | 1.3E-01 | 2.12 | 0.99 | 0.41 | 0.13 | 0.13 | 0.00 |
| Task 2 | Prediction | MFGGABA_DISTANCE_MeanDriftRate | 147 | 0.05 | 5.1E-01 | 0.03 | 7.0E-01 | 1.84 | 0.99 | 0.09 | 0.26 | 0.27 | 0.00 |
| Task 2 | Prediction | MFGNAA_overall_MeanDriftRate | 147 | -0.07 | 1.7E-01 | -0.07 | 2.1E-01 | 5.82 | 0.99 | 0.36 | 0.64 | 0.64 | 0.00 |
| Task 2 | Prediction | MFGNAA_SNARC_MeanDriftRate | 150 | 0.17 | 3.2E-02 | 0.02 | 8.6E-01 | 2.16 | 0.99 | 0.65 | 0.12 | 0.11 | 0.02 |
| Task 2 | Prediction | MFGNAA_DISTANCE_MeanDriftRate | 152 | -0.18 | 1.6E-02 | -0.08 | 3.9E-01 | 1.96 | 0.99 | 0.18 | 0.27 | 0.25 | 0.02 |
| Task 2 | Prediction | IPSGLU_overall_BoundarySeparation | 147 | -0.15 | 1.2E-02 | 0.11 | 1.1E-01 | 6.76 | 0.98 | 0.02 | 0.60 | 0.58 | 0.01 |
| Task 2 | Prediction | IPSGLU_SNARC_BoundarySeparation | 151 | -0.01 | 8.4E-01 | -0.07 | 3.2E-01 | 1.53 | 0.99 | 0.73 | 0.59 | 0.59 | 0.00 |
| Task 2 | Prediction | IPSGLU_DISTANCE_BoundarySeparation | 152 | 0.16 | 5.7E-02 | -0.13 | 1.1E-01 | 2.23 | 0.99 | 0.61 | 0.29 | 0.27 | 0.02 |
| Task 2 | Prediction | IPSGABA_overall_BoundarySeparation | 147 | -0.11 | 6.8E-02 | 0.00 | 9.5E-01 | 6.05 | 0.98 | 0.06 | 0.58 | 0.58 | 0.00 |
| Task 2 | Prediction | IPSGABA_SNARC_BoundarySeparation | 151 | 0.04 | 5.8E-01 | -0.06 | 4.0E-01 | 1.41 | 0.99 | 0.65 | 0.58 | 0.58 | 0.00 |
| Task 2 | Prediction | IPSGABA_DISTANCE_BoundarySeparation | 152 | 0.04 | 6.2E-01 | 0.10 | 2.3E-01 | 2.08 | 0.99 | 0.70 | 0.27 | 0.27 | 0.00 |
| Task 2 | Prediction | IPSNAA_overall_BoundarySeparation | 148 | -0.02 | 7.6E-01 | 0.00 | 1.0E+00 | 6.10 | 0.99 | 0.09 | 0.57 | 0.58 | 0.00 |
| Task 2 | Prediction | IPSNAA_SNARC_BoundarySeparation | 152 | 0.00 | 9.7E-01 | -0.11 | 1.1E-01 | 1.40 | 0.99 | 0.51 | 0.59 | 0.59 | 0.00 |
| Task 2 | Prediction | IPSNAA_DISTANCE_BoundarySeparation | 153 | -0.02 | 7.9E-01 | 0.03 | 7.2E-01 | 2.02 | 0.99 | 0.58 | 0.26 | 0.26 | 0.00 |
| Task 2 | Prediction | MFGGLU_overall_BoundarySeparation | 146 | -0.13 | 4.8E-02 | 0.07 | 3.2E-01 | 6.15 | 0.98 | 0.01 | 0.60 | 0.59 | 0.01 |
| Task 2 | Prediction | MFGGLU_SNARC_BoundarySeparation | 150 | -0.06 | 3.3E-01 | -0.07 | 1.9E-01 | 1.40 | 0.99 | 0.82 | 0.58 | 0.58 | 0.00 |
| Task 2 | Prediction | MFGGLU_DISTANCE_BoundarySeparation | 151 | 0.08 | 3.5E-01 | -0.10 | 1.7E-01 | 2.29 | 0.99 | 0.28 | 0.27 | 0.27 | 0.00 |
| Task 2 | Prediction | MFGGABA_overall_BoundarySeparation | 143 | -0.14 | 3.6E-02 | 0.06 | 3.1E-01 | 5.80 | 0.98 | 0.01 | 0.62 | 0.61 | 0.01 |
| Task 2 | Prediction | MFGGABA_SNARC_BoundarySeparation | 145 | 0.00 | 9.5E-01 | 0.06 | 3.8E-01 | 1.30 | 0.99 | 0.54 | 0.58 | 0.58 | 0.00 |
| Task 2 | Prediction | MFGGABA_DISTANCE_BoundarySeparation | 146 | -0.06 | 4.2E-01 | -0.02 | 8.2E-01 | 2.03 | 0.99 | 0.45 | 0.27 | 0.27 | 0.00 |
| Task 2 | Prediction | MFGNAA_overall_BoundarySeparation | 146 | 0.00 | 9.5E-01 | 0.04 | 5.5E-01 | 5.87 | 0.98 | 0.01 | 0.61 | 0.61 | 0.00 |
| Task 2 | Prediction | MFGNAA_SNARC_BoundarySeparation | 149 | 0.01 | 8.9E-01 | -0.11 | 8.6E-02 | 1.59 | 0.99 | 0.82 | 0.58 | 0.58 | 0.00 |
| Task 2 | Prediction | MFGNAA_DISTANCE_BoundarySeparation | 150 | 0.00 | 9.7E-01 | 0.00 | 9.9E-01 | 2.20 | 0.99 | 0.56 | 0.26 | 0.26 | 0.00 |
| Task 2 | Prediction | IPSGLU_overall_NonDecisionTime | 146 | -0.19 | 7.0E-03 | 0.17 | 7.5E-03 | 5.64 | 0.97 | 0.01 | 0.68 | 0.65 | 0.02 |
| Task 2 | Prediction | IPSGLU_SNARC_NonDecisionTime | 154 | 0.04 | 5.4E-01 | -0.05 | 4.4E-01 | 1.53 | 0.98 | 0.07 | 0.56 | 0.56 | 0.00 |
| Task 2 | Prediction | IPSGLU_DISTANCE_NonDecisionTime | 152 | 0.24 | 1.3E-02 | -0.16 | 6.6E-02 | 2.03 | 0.98 | 0.02 | 0.46 | 0.42 | 0.04 |
| Task 2 | Prediction | IPSGABA_overall_NonDecisionTime | 148 | 0.24 | 4.2E-04 | -0.03 | 6.4E-01 | 5.41 | 0.97 | 0.00 | 0.68 | 0.64 | 0.04 |
| Task 2 | Prediction | IPSGABA_SNARC_NonDecisionTime | 154 | 0.01 | 9.0E-01 | 0.00 | 9.6E-01 | 1.38 | 0.99 | 0.09 | 0.55 | 0.55 | 0.00 |
| Task 2 | Prediction | IPSGABA_DISTANCE_NonDecisionTime | 151 | -0.07 | 3.3E-01 | 0.06 | 3.3E-01 | 1.88 | 0.96 | 0.00 | 0.43 | 0.43 | 0.00 |
| Task 2 | Prediction | IPSNAA_overall_NonDecisionTime | 146 | 0.10 | 1.2E-01 | -0.01 | 8.4E-01 | 5.11 | 0.96 | 0.00 | 0.64 | 0.64 | 0.01 |
| Task 2 | Prediction | IPSNAA_SNARC_NonDecisionTime | 155 | -0.03 | 6.2E-01 | -0.06 | 3.6E-01 | 1.41 | 0.99 | 0.08 | 0.55 | 0.56 | 0.00 |
| Task 2 | Prediction | IPSNAA_DISTANCE_NonDecisionTime | 153 | -0.01 | 8.6E-01 | 0.02 | 8.3E-01 | 1.86 | 0.96 | 0.00 | 0.40 | 0.40 | 0.00 |
| Task 2 | Prediction | MFGGLU_overall_NonDecisionTime | 144 | -0.22 | 8.6E-04 | 0.07 | 1.5E-01 | 4.79 | 0.97 | 0.00 | 0.68 | 0.65 | 0.03 |
| Task 2 | Prediction | MFGGLU_SNARC_NonDecisionTime | 153 | -0.01 | 9.1E-01 | -0.06 | 2.6E-01 | 1.42 | 0.99 | 0.18 | 0.54 | 0.55 | 0.00 |
| Task 2 | Prediction | MFGGLU_DISTANCE_NonDecisionTime | 150 | 0.17 | 3.4E-02 | -0.12 | 5.2E-02 | 1.98 | 0.96 | 0.00 | 0.44 | 0.43 | 0.01 |
| Task 2 | Prediction | MFGGABA_overall_NonDecisionTime | 142 | 0.15 | 1.5E-02 | -0.09 | 8.0E-02 | 4.99 | 0.97 | 0.00 | 0.65 | 0.63 | 0.02 |
| Task 2 | Prediction | MFGGABA_SNARC_NonDecisionTime | 148 | -0.06 | 4.4E-01 | 0.04 | 5.6E-01 | 1.24 | 0.99 | 0.25 | 0.54 | 0.54 | 0.00 |
| Task 2 | Prediction | MFGGABA_DISTANCE_NonDecisionTime | 145 | 0.03 | 7.7E-01 | 0.01 | 8.6E-01 | 1.82 | 0.96 | 0.00 | 0.44 | 0.44 | 0.00 |
| Task 2 | Prediction | MFGNAA_overall_NonDecisionTime | 144 | 0.14 | 3.3E-02 | -0.03 | 5.9E-01 | 5.04 | 0.96 | 0.00 | 0.66 | 0.65 | 0.01 |
| Task 2 | Prediction | MFGNAA_SNARC_NonDecisionTime | 152 | -0.03 | 6.5E-01 | -0.09 | 9.6E-02 | 1.55 | 0.99 | 0.23 | 0.54 | 0.55 | 0.00 |
| Task 2 | Prediction | MFGNAA_DISTANCE_NonDecisionTime | 149 | -0.04 | 6.8E-01 | -0.07 | 2.7E-01 | 2.03 | 0.96 | 0.00 | 0.41 | 0.42 | 0.00 |
| Task 3 | Prediction | IPSGLU_overall_MeanDriftRate | 139 | 0.01 | 8.7E-01 | -0.01 | 8.9E-01 | 3.28 | 0.99 | 0.45 | 0.64 | 0.64 | 0.00 |
| Task 3 | Prediction | IPSGLU_DISTANCE_MeanDriftRate | 138 | -0.06 | 4.6E-01 | -0.17 | 8.4E-02 | 2.71 | 0.99 | 0.21 | 0.10 | 0.10 | 0.00 |
| Task 3 | Prediction | IPSGABA_overall_MeanDriftRate | 140 | -0.01 | 8.8E-01 | 0.04 | 5.2E-01 | 2.89 | 0.99 | 0.55 | 0.64 | 0.64 | 0.00 |
| Task 3 | Prediction | IPSGABA_DISTANCE_MeanDriftRate | 139 | -0.05 | 5.8E-01 | -0.04 | 6.7E-01 | 2.81 | 0.99 | 0.63 | 0.08 | 0.08 | 0.00 |
| Task 3 | Prediction | IPSNAA_overall_MeanDriftRate | 140 | -0.07 | 2.4E-01 | -0.06 | 2.4E-01 | 2.82 | 0.99 | 0.69 | 0.65 | 0.64 | 0.00 |
| Task 3 | Prediction | IPSNAA_DISTANCE_MeanDriftRate | 139 | -0.07 | 3.9E-01 | -0.14 | 1.2E-01 | 2.79 | 0.99 | 0.26 | 0.10 | 0.10 | 0.00 |
| Task 3 | Prediction | MFGGLU_overall_MeanDriftRate | 136 | -0.13 | 4.1E-02 | -0.11 | 9.5E-02 | 3.04 | 0.99 | 0.74 | 0.66 | 0.65 | 0.01 |
| Task 3 | Prediction | MFGGLU_DISTANCE_MeanDriftRate | 136 | -0.02 | 9.0E-01 | -0.12 | 2.2E-01 | 2.76 | 0.99 | 0.44 | 0.08 | 0.09 | -0.01 |
| Task 3 | Prediction | MFGGABA_overall_MeanDriftRate | 132 | 0.06 | 2.4E-01 | 0.08 | 1.7E-01 | 2.75 | 1.00 | 0.96 | 0.64 | 0.64 | 0.00 |
| Task 3 | Prediction | MFGGABA_DISTANCE_MeanDriftRate | 131 | -0.03 | 7.6E-01 | 0.14 | 1.1E-01 | 2.56 | 0.99 | 0.36 | 0.09 | 0.10 | -0.01 |
| Task 3 | Prediction | MFGNAA_overall_MeanDriftRate | 136 | -0.07 | 2.0E-01 | -0.08 | 1.6E-01 | 2.74 | 0.99 | 0.61 | 0.63 | 0.63 | 0.00 |
| Task 3 | Prediction | MFGNAA_DISTANCE_MeanDriftRate | 135 | 0.04 | 6.8E-01 | 0.01 | 9.5E-01 | 2.70 | 0.99 | 0.49 | 0.06 | 0.07 | -0.01 |
| Task 3 | Prediction | IPSGLU_overall_BoundarySeparation | 139 | -0.13 | 1.2E-01 | 0.12 | 1.2E-01 | 3.84 | 0.99 | 0.30 | 0.55 | 0.54 | 0.01 |
| Task 3 | Prediction | IPSGLU_DISTANCE_BoundarySeparation | 138 | -0.04 | 3.9E-01 | 0.08 | 1.7E-01 | 1.77 | 0.99 | 0.55 | 0.67 | 0.67 | 0.00 |
| Task 3 | Prediction | IPSGABA_overall_BoundarySeparation | 139 | 0.16 | 2.2E-02 | -0.05 | 4.9E-01 | 3.76 | 0.99 | 0.39 | 0.54 | 0.52 | 0.02 |
| Task 3 | Prediction | IPSGABA_DISTANCE_BoundarySeparation | 139 | 0.11 | 6.4E-02 | 0.03 | 6.1E-01 | 1.38 | 0.99 | 0.67 | 0.67 | 0.67 | 0.01 |
| Task 3 | Prediction | IPSNAA_overall_BoundarySeparation | 139 | 0.07 | 3.4E-01 | -0.03 | 7.3E-01 | 3.76 | 0.99 | 0.12 | 0.52 | 0.52 | 0.00 |
| Task 3 | Prediction | IPSNAA_DISTANCE_BoundarySeparation | 138 | 0.12 | 2.9E-02 | -0.07 | 1.7E-01 | 1.38 | 0.99 | 0.40 | 0.66 | 0.65 | 0.01 |
| Task 3 | Prediction | MFGGLU_overall_BoundarySeparation | 136 | -0.14 | 6.7E-02 | 0.03 | 7.0E-01 | 3.69 | 0.99 | 0.22 | 0.52 | 0.51 | 0.01 |
| Task 3 | Prediction | MFGGLU_DISTANCE_BoundarySeparation | 136 | 0.03 | 6.4E-01 | 0.00 | 9.6E-01 | 1.45 | 0.99 | 0.71 | 0.65 | 0.65 | 0.00 |
| Task 3 | Prediction | MFGGABA_overall_BoundarySeparation | 132 | 0.04 | 5.6E-01 | 0.15 | 1.2E-02 | 3.72 | 0.99 | 0.81 | 0.55 | 0.55 | 0.00 |
| Task 3 | Prediction | MFGGABA_DISTANCE_BoundarySeparation | 131 | 0.03 | 7.0E-01 | -0.02 | 8.0E-01 | 1.25 | 1.00 | 0.87 | 0.65 | 0.65 | 0.00 |
| Task 3 | Prediction | MFGNAA_overall_BoundarySeparation | 136 | 0.08 | 2.6E-01 | 0.02 | 7.8E-01 | 3.75 | 0.99 | 0.38 | 0.53 | 0.53 | 0.00 |
| Task 3 | Prediction | MFGNAA_DISTANCE_BoundarySeparation | 135 | 0.05 | 4.3E-01 | 0.01 | 8.5E-01 | 1.42 | 0.99 | 0.76 | 0.65 | 0.65 | 0.00 |
| Task 3 | Prediction | IPSGLU_overall_NonDecisionTime | 137 | -0.16 | 1.3E-02 | 0.03 | 6.8E-01 | 3.53 | 0.96 | 0.00 | 0.50 | 0.48 | 0.02 |
| Task 3 | Prediction | IPSGLU_DISTANCE_NonDecisionTime | 138 | -0.01 | 8.8E-01 | 0.11 | 6.2E-02 | 1.79 | 0.96 | 0.00 | 0.70 | 0.70 | 0.00 |
| Task 3 | Prediction | IPSGABA_overall_NonDecisionTime | 138 | 0.34 | 3.4E-08 | -0.12 | 2.5E-02 | 3.55 | 0.98 | 0.02 | 0.60 | 0.49 | 0.11 |
| Task 3 | Prediction | IPSGABA_DISTANCE_NonDecisionTime | 139 | 0.14 | 1.8E-02 | 0.01 | 9.1E-01 | 1.36 | 0.97 | 0.00 | 0.71 | 0.69 | 0.02 |
| Task 3 | Prediction | IPSNAA_overall_NonDecisionTime | 139 | 0.21 | 9.9E-03 | -0.14 | 5.2E-02 | 3.59 | 0.96 | 0.00 | 0.50 | 0.46 | 0.03 |
| Task 3 | Prediction | IPSNAA_DISTANCE_NonDecisionTime | 139 | 0.11 | 6.9E-02 | -0.01 | 9.1E-01 | 1.35 | 0.97 | 0.00 | 0.70 | 0.69 | 0.01 |
| Task 3 | Prediction | MFGGLU_overall_NonDecisionTime | 135 | -0.07 | 5.2E-01 | -0.02 | 8.4E-01 | 3.48 | 0.96 | 0.00 | 0.44 | 0.45 | 0.00 |
| Task 3 | Prediction | MFGGLU_DISTANCE_NonDecisionTime | 137 | 0.13 | 8.0E-02 | -0.01 | 8.7E-01 | 1.44 | 0.95 | 0.00 | 0.67 | 0.66 | 0.01 |
| Task 3 | Prediction | MFGGABA_overall_NonDecisionTime | 129 | 0.06 | 5.5E-01 | -0.08 | 2.7E-01 | 3.41 | 0.97 | 0.01 | 0.48 | 0.48 | 0.00 |
| Task 3 | Prediction | MFGGABA_DISTANCE_NonDecisionTime | 131 | 0.08 | 1.8E-01 | -0.05 | 2.8E-01 | 1.24 | 0.97 | 0.01 | 0.69 | 0.68 | 0.00 |
| Task 3 | Prediction | MFGNAA_overall_NonDecisionTime | 134 | 0.21 | 1.3E-02 | -0.11 | 5.2E-02 | 3.55 | 0.97 | 0.00 | 0.54 | 0.50 | 0.03 |
| Task 3 | Prediction | MFGNAA_DISTANCE_NonDecisionTime | 135 | 0.11 | 2.17 | 0.06 | -0.01 | 1.39 | 0.97 | 0.01 | 0.69 | 0.68 | 0.01 |
