# Supplementary material for: Dissecting the chain of information processing and its interplay with neurochemicals and fluid intelligence across development
Source: eLife. 2023 Sep 29;12:e84086. doi: 10.7554/eLife.84086 (PMC10541179; doi:10.7554/eLife.84086)
Supplement: Supplementary file 6. — To assess whether behaviour during the second assessment (i.e., A2) was predicted by neuroimaging measures during the first assessment (i.e., A1) while controlling for behaviour during the first assessment (i.e., A1), we employed SF6-eq1 as can be seen below which is a variant of (Equation 5) that additionally includes behaviour during the first assessment as can be seen below highlighted in bold. The first column denotes the task (i.e., Task 1, Task 2 or Task 3), the second column (i.e., “Prediction”) concerns predicting behaviour during the second assessment based on neurochemicals during the first assessment. The third column has three names separated by underscores, the first name corresponds to the region and the neurochemical used where IPS=intraparietal sulcus and MFG=middle frontal gyrus, and GLU=glutamate, GABA=gamma-Aminobutyric acid and NAA=N-acetylaspartate, the third name corresponds to the diffusion parameter that was used as the dependent variable, and the second name corresponds to the way each diffusion parameter was calculated where overall=the diffusion parameter was calculated across all trials, AL=alerting network, OR=orienting network, EX=executive network, DISTANCE=the effect of distance, SNARC=the effect of SNARC. For the rest of the columns (df=degrees of freedom, β=standardized coefficient, PBO=bootstrapped P-value), where β (column 5) and PBO (column 6) correspond to the neurochemical*age interaction and β (column 7) and PBO (column 8) correspond to the main effect of the neurochemical. The column “int_R2” is the adjusted R-squared of the model, and the column “non_int_R2” is the adjusted R-squared of the same model, but when omitting the interaction predictor and the column “delta_R2” is the difference between “int_R2” and “non_int_R2”. [file elife-84086-supp6.docx]

**Supplementary File 6.** Statistical results of SF6-eq1 (see below). To assess whether behaviour during the second assessment (i.e., A2) was predicted by neuroimaging measures during the first assessment (i.e., A1) while controlling for behaviour during the first assessment (i.e., A1), we employed SF6-eq1 as can be seen below which is a variant of eq3 that additionally includes behaviour during the first assessment as can be seen below highlighted in bold. The first column denotes the task (i.e., **Task 1**, **Task 2** or **Task 3**), the second column (i.e., “Prediction”) concerns predicting behaviour during the second assessment based on neurochemicals during the first assessment. The third column has three names separated by underscores, the first name corresponds to the region and the neurochemical used where IPS=intraparietal sulcus and MFG=middle frontal gyrus, and GLU=glutamate, GABA=gamma-Aminobutyric acid and NAA=N-acetylaspartate, the third name corresponds to the diffusion parameter that was used as the dependent variable, and the second name corresponds to the way each diffusion parameter was calculated where overall=the diffusion parameter was calculated across all trials, AL=alerting network, OR=orienting network, EX=executive network, DISTANCE=the effect of distance, SNARC= the effect of SNARC. For the rest of the columns (df=degrees of freedom, β=standardized coefficient, P_BO_=bootstrapped P-value), where β (column 5) and P_BO_ (column 6) correspond to the neurochemical*age interaction and β (column 7) and P_BO_ (column 8) correspond to the main effect of the neurochemical. The column “int_R^2^” is the adjusted R-squared of the model, and the column “non_int_R^2^” is the adjusted R-squared of the same model, but when omitting the interaction predictor and the column “delta_R^2^” is the difference between “int_R^2^” and “non_int_R^2^”.

*behavioural score (A2) ~ neurotransmitter (A1)+age (A1)+neurotransmitter (A1)*age (A1)+control behavioural score 1 (A1)+control behavioural score 2 (A1)+control behavioural score 1 (A2)+control behavioural score 2 (A2) +* ***behavioural score (A1)*** *+ age (A2)*

(SF6-eq1)

|  |  |  | **df** | **β** | **P_BO_** | **β** | **P_BO_** | **int_R^2^** | **non_int_R^2^** | **delta_R^2^** |
| --- | --- | --- | --- | --- | --- | --- | --- | --- | --- | --- |
| Task 1 | Prediction | IPSGLU_overall_MeanDriftRate | 160 | -0.08 | 7.8E-02 | -0.03 | 6.6E-01 | 0.72 | 0.71 | 0.00 |
| Task 1 | Prediction | IPSGLU_AL_MeanDriftRate | 158 | 0.11 | 1.5E-01 | 0.06 | 4.8E-01 | 0.37 | 0.36 | 0.01 |
| Task 1 | Prediction | IPSGLU_OR_MeanDriftRate | 155 | -0.02 | 7.0E-01 | -0.03 | 7.2E-01 | 0.41 | 0.41 | 0.00 |
| Task 1 | Prediction | IPSGLU_EX_MeanDriftRate | 158 | -0.13 | 9.0E-02 | -0.14 | 1.9E-01 | 0.24 | 0.23 | 0.01 |
| Task 1 | Prediction | IPSGABA_overall_MeanDriftRate | 160 | -0.14 | 4.0E-03 | 0.03 | 5.1E-01 | 0.72 | 0.70 | 0.01 |
| Task 1 | Prediction | IPSGABA_AL_MeanDriftRate | 158 | 0.06 | 3.8E-01 | 0.02 | 8.4E-01 | 0.38 | 0.39 | 0.00 |
| Task 1 | Prediction | IPSGABA_OR_MeanDriftRate | 155 | -0.01 | 9.1E-01 | -0.09 | 2.8E-01 | 0.42 | 0.43 | 0.00 |
| Task 1 | Prediction | IPSGABA_EX_MeanDriftRate | 158 | -0.03 | 7.2E-01 | 0.02 | 8.0E-01 | 0.22 | 0.22 | 0.00 |
| Task 1 | Prediction | IPSNAA_overall_MeanDriftRate | 161 | -0.02 | 6.4E-01 | -0.05 | 3.3E-01 | 0.71 | 0.71 | 0.00 |
| Task 1 | Prediction | IPSNAA_AL_MeanDriftRate | 159 | 0.04 | 6.4E-01 | 0.04 | 6.2E-01 | 0.36 | 0.36 | 0.00 |
| Task 1 | Prediction | IPSNAA_OR_MeanDriftRate | 157 | -0.01 | 8.7E-01 | -0.03 | 6.9E-01 | 0.35 | 0.36 | 0.00 |
| Task 1 | Prediction | IPSNAA_EX_MeanDriftRate | 159 | -0.06 | 4.2E-01 | -0.16 | 5.3E-02 | 0.24 | 0.24 | 0.00 |
| Task 1 | Prediction | MFGGLU_overall_MeanDriftRate | 159 | -0.03 | 5.6E-01 | 0.01 | 9.2E-01 | 0.72 | 0.72 | 0.00 |
| Task 1 | Prediction | MFGGLU_AL_MeanDriftRate | 155 | -0.10 | 1.6E-01 | -0.12 | 8.0E-02 | 0.48 | 0.48 | 0.00 |
| Task 1 | Prediction | MFGGLU_OR_MeanDriftRate | 154 | 0.04 | 6.2E-01 | 0.09 | 2.0E-01 | 0.45 | 0.45 | 0.00 |
| Task 1 | Prediction | MFGGLU_EX_MeanDriftRate | 157 | -0.08 | 3.2E-01 | -0.09 | 2.8E-01 | 0.25 | 0.25 | 0.00 |
| Task 1 | Prediction | MFGGABA_overall_MeanDriftRate | 154 | 0.09 | 6.7E-02 | 0.03 | 5.2E-01 | 0.72 | 0.71 | 0.00 |
| Task 1 | Prediction | MFGGABA_AL_MeanDriftRate | 152 | 0.03 | 6.6E-01 | 0.00 | 9.8E-01 | 0.40 | 0.40 | 0.00 |
| Task 1 | Prediction | MFGGABA_OR_MeanDriftRate | 150 | 0.19 | 3.4E-02 | -0.08 | 2.8E-01 | 0.41 | 0.38 | 0.02 |
| Task 1 | Prediction | MFGGABA_EX_MeanDriftRate | 151 | 0.15 | 3.3E-02 | 0.06 | 4.1E-01 | 0.26 | 0.25 | 0.01 |
| Task 1 | Prediction | MFGNAA_overall_MeanDriftRate | 158 | -0.04 | 4.8E-01 | -0.05 | 4.1E-01 | 0.71 | 0.71 | 0.00 |
| Task 1 | Prediction | MFGNAA_AL_MeanDriftRate | 156 | -0.02 | 8.3E-01 | -0.16 | 3.6E-02 | 0.41 | 0.42 | 0.00 |
| Task 1 | Prediction | MFGNAA_OR_MeanDriftRate | 155 | -0.05 | 6.0E-01 | 0.11 | 2.0E-01 | 0.38 | 0.38 | 0.00 |
| Task 1 | Prediction | MFGNAA_EX_MeanDriftRate | 156 | -0.02 | 7.7E-01 | -0.12 | 1.6E-01 | 0.24 | 0.25 | 0.00 |
| Task 1 | Prediction | IPSGLU_overall_BoundarySeparation | 160 | -0.12 | 2.1E-02 | -0.04 | 4.7E-01 | 0.72 | 0.71 | 0.01 |
| Task 1 | Prediction | IPSGLU_AL_BoundarySeparation | 157 | 0.08 | 3.3E-01 | 0.11 | 1.6E-01 | 0.53 | 0.52 | 0.00 |
| Task 1 | Prediction | IPSGLU_OR_BoundarySeparation | 155 | 0.01 | 9.0E-01 | -0.08 | 2.3E-01 | 0.54 | 0.55 | 0.00 |
| Task 1 | Prediction | IPSGLU_EX_BoundarySeparation | 156 | -0.05 | 5.4E-01 | -0.05 | 5.3E-01 | 0.55 | 0.55 | 0.00 |
| Task 1 | Prediction | IPSGABA_overall_BoundarySeparation | 160 | -0.10 | 4.2E-02 | 0.01 | 8.3E-01 | 0.70 | 0.70 | 0.01 |
| Task 1 | Prediction | IPSGABA_AL_BoundarySeparation | 158 | 0.00 | 9.8E-01 | -0.06 | 4.4E-01 | 0.52 | 0.52 | 0.00 |
| Task 1 | Prediction | IPSGABA_OR_BoundarySeparation | 156 | 0.11 | 6.1E-02 | -0.13 | 3.3E-02 | 0.51 | 0.51 | 0.01 |
| Task 1 | Prediction | IPSGABA_EX_BoundarySeparation | 157 | 0.03 | 6.4E-01 | -0.04 | 6.0E-01 | 0.54 | 0.55 | 0.00 |
| Task 1 | Prediction | IPSNAA_overall_BoundarySeparation | 161 | 0.00 | 9.7E-01 | -0.08 | 1.8E-01 | 0.71 | 0.71 | 0.00 |
| Task 1 | Prediction | IPSNAA_AL_BoundarySeparation | 158 | -0.05 | 4.5E-01 | 0.16 | 2.5E-02 | 0.54 | 0.54 | 0.00 |
| Task 1 | Prediction | IPSNAA_OR_BoundarySeparation | 156 | -0.03 | 6.9E-01 | -0.02 | 8.0E-01 | 0.54 | 0.54 | 0.00 |
| Task 1 | Prediction | IPSNAA_EX_BoundarySeparation | 157 | 0.19 | 5.6E-03 | -0.14 | 4.1E-02 | 0.58 | 0.55 | 0.03 |
| Task 1 | Prediction | MFGGLU_overall_BoundarySeparation | 159 | -0.07 | 2.8E-01 | -0.05 | 4.3E-01 | 0.71 | 0.71 | 0.00 |
| Task 1 | Prediction | MFGGLU_AL_BoundarySeparation | 156 | 0.11 | 1.5E-01 | -0.01 | 9.1E-01 | 0.56 | 0.56 | 0.00 |
| Task 1 | Prediction | MFGGLU_OR_BoundarySeparation | 154 | 0.00 | 9.6E-01 | 0.07 | 2.6E-01 | 0.57 | 0.57 | 0.00 |
| Task 1 | Prediction | MFGGLU_EX_BoundarySeparation | 156 | 0.07 | 3.8E-01 | -0.13 | 5.2E-02 | 0.53 | 0.53 | 0.00 |
| Task 1 | Prediction | MFGGABA_overall_BoundarySeparation | 154 | -0.09 | 5.6E-02 | 0.08 | 1.0E-01 | 0.72 | 0.72 | 0.01 |
| Task 1 | Prediction | MFGGABA_AL_BoundarySeparation | 151 | -0.02 | 7.5E-01 | 0.05 | 3.2E-01 | 0.53 | 0.54 | 0.00 |
| Task 1 | Prediction | MFGGABA_OR_BoundarySeparation | 150 | 0.14 | 2.2E-02 | -0.12 | 4.5E-02 | 0.56 | 0.55 | 0.01 |
| Task 1 | Prediction | MFGGABA_EX_BoundarySeparation | 149 | 0.12 | 4.6E-02 | -0.08 | 1.6E-01 | 0.58 | 0.57 | 0.01 |
| Task 1 | Prediction | MFGNAA_overall_BoundarySeparation | 158 | -0.02 | 7.1E-01 | -0.01 | 8.2E-01 | 0.71 | 0.71 | 0.00 |
| Task 1 | Prediction | MFGNAA_AL_BoundarySeparation | 155 | -0.01 | 8.6E-01 | -0.01 | 9.4E-01 | 0.53 | 0.54 | 0.00 |
| Task 1 | Prediction | MFGNAA_OR_BoundarySeparation | 155 | -0.02 | 8.1E-01 | 0.14 | 4.8E-02 | 0.54 | 0.55 | 0.00 |
| Task 1 | Prediction | MFGNAA_EX_BoundarySeparation | 155 | 0.19 | 2.0E-03 | -0.14 | 2.6E-02 | 0.57 | 0.54 | 0.03 |
| Task 1 | Prediction | IPSGLU_overall_NonDecisionTime | 158 | -0.04 | 5.6E-01 | 0.03 | 6.6E-01 | 0.70 | 0.70 | 0.00 |
| Task 1 | Prediction | IPSGLU_AL_NonDecisionTime | 154 | -0.28 | 2.4E-04 | 0.20 | 6.1E-03 | 0.43 | 0.37 | 0.06 |
| Task 1 | Prediction | IPSGLU_OR_NonDecisionTime | 153 | -0.01 | 9.3E-01 | -0.15 | 2.9E-02 | 0.44 | 0.44 | 0.00 |
| Task 1 | Prediction | IPSGLU_EX_NonDecisionTime | 154 | -0.16 | 8.5E-03 | 0.21 | 2.4E-03 | 0.55 | 0.53 | 0.02 |
| Task 1 | Prediction | IPSGABA_overall_NonDecisionTime | 157 | 0.09 | 1.0E-01 | -0.04 | 3.5E-01 | 0.71 | 0.70 | 0.00 |
| Task 1 | Prediction | IPSGABA_AL_NonDecisionTime | 154 | 0.09 | 2.5E-01 | 0.01 | 9.2E-01 | 0.38 | 0.38 | 0.00 |
| Task 1 | Prediction | IPSGABA_OR_NonDecisionTime | 154 | 0.12 | 8.2E-02 | -0.03 | 6.4E-01 | 0.44 | 0.43 | 0.01 |
| Task 1 | Prediction | IPSGABA_EX_NonDecisionTime | 155 | 0.03 | 7.0E-01 | 0.01 | 8.7E-01 | 0.57 | 0.57 | 0.00 |
| Task 1 | Prediction | IPSNAA_overall_NonDecisionTime | 158 | 0.02 | 6.7E-01 | -0.08 | 6.6E-02 | 0.71 | 0.71 | 0.00 |
| Task 1 | Prediction | IPSNAA_AL_NonDecisionTime | 155 | -0.01 | 8.7E-01 | 0.14 | 7.1E-02 | 0.38 | 0.39 | 0.00 |
| Task 1 | Prediction | IPSNAA_OR_NonDecisionTime | 154 | 0.15 | 6.8E-02 | -0.16 | 3.0E-02 | 0.46 | 0.44 | 0.02 |
| Task 1 | Prediction | IPSNAA_EX_NonDecisionTime | 156 | 0.18 | 1.2E-02 | -0.06 | 4.2E-01 | 0.58 | 0.55 | 0.02 |
| Task 1 | Prediction | MFGGLU_overall_NonDecisionTime | 157 | -0.06 | 3.0E-01 | 0.06 | 2.0E-01 | 0.73 | 0.73 | 0.00 |
| Task 1 | Prediction | MFGGLU_AL_NonDecisionTime | 152 | -0.09 | 3.1E-01 | 0.10 | 2.0E-01 | 0.37 | 0.36 | 0.00 |
| Task 1 | Prediction | MFGGLU_OR_NonDecisionTime | 152 | -0.14 | 6.9E-02 | -0.02 | 8.4E-01 | 0.47 | 0.46 | 0.01 |
| Task 1 | Prediction | MFGGLU_EX_NonDecisionTime | 153 | -0.05 | 5.5E-01 | 0.04 | 5.4E-01 | 0.52 | 0.52 | 0.00 |
| Task 1 | Prediction | MFGGABA_overall_NonDecisionTime | 152 | 0.03 | 5.1E-01 | -0.04 | 3.3E-01 | 0.73 | 0.73 | 0.00 |
| Task 1 | Prediction | MFGGABA_AL_NonDecisionTime | 148 | -0.03 | 7.9E-01 | 0.06 | 4.2E-01 | 0.34 | 0.34 | 0.00 |
| Task 1 | Prediction | MFGGABA_OR_NonDecisionTime | 147 | 0.08 | 2.6E-01 | -0.06 | 2.8E-01 | 0.48 | 0.48 | 0.00 |
| Task 1 | Prediction | MFGGABA_EX_NonDecisionTime | 148 | 0.01 | 9.2E-01 | -0.03 | 7.0E-01 | 0.58 | 0.58 | 0.00 |
| Task 1 | Prediction | MFGNAA_overall_NonDecisionTime | 156 | -0.04 | 5.4E-01 | 0.08 | 1.2E-01 | 0.73 | 0.73 | 0.00 |
| Task 1 | Prediction | MFGNAA_AL_NonDecisionTime | 151 | 0.03 | 6.7E-01 | 0.08 | 3.5E-01 | 0.36 | 0.36 | 0.00 |
| Task 1 | Prediction | MFGNAA_OR_NonDecisionTime | 153 | 0.19 | 3.0E-02 | -0.11 | 1.6E-01 | 0.52 | 0.49 | 0.03 |
| Task 1 | Prediction | MFGNAA_EX_NonDecisionTime | 153 | 0.17 | 1.8E-02 | -0.07 | 3.0E-01 | 0.58 | 0.56 | 0.02 |
| Task 2 | Prediction | IPSGLU_overall_MeanDriftRate | 147 | -0.05 | 3.6E-01 | 0.03 | 6.8E-01 | 0.73 | 0.73 | 0.00 |
| Task 2 | Prediction | IPSGLU_SNARC_MeanDriftRate | 151 | -0.01 | 8.7E-01 | 0.11 | 2.7E-01 | 0.12 | 0.12 | -0.01 |
| Task 2 | Prediction | IPSGLU_DISTANCE_MeanDriftRate | 151 | 0.13 | 8.4E-02 | -0.04 | 6.8E-01 | 0.29 | 0.28 | 0.01 |
| Task 2 | Prediction | IPSGABA_overall_MeanDriftRate | 147 | -0.17 | 1.2E-03 | -0.02 | 7.6E-01 | 0.75 | 0.73 | 0.02 |
| Task 2 | Prediction | IPSGABA_SNARC_MeanDriftRate | 151 | 0.14 | 6.9E-02 | 0.01 | 8.8E-01 | 0.12 | 0.11 | 0.01 |
| Task 2 | Prediction | IPSGABA_DISTANCE_MeanDriftRate | 151 | -0.08 | 3.0E-01 | -0.14 | 1.7E-01 | 0.29 | 0.29 | 0.00 |
| Task 2 | Prediction | IPSNAA_overall_MeanDriftRate | 148 | -0.03 | 4.7E-01 | -0.07 | 2.1E-01 | 0.73 | 0.73 | 0.00 |
| Task 2 | Prediction | IPSNAA_SNARC_MeanDriftRate | 152 | 0.08 | 2.8E-01 | 0.07 | 4.4E-01 | 0.11 | 0.12 | 0.00 |
| Task 2 | Prediction | IPSNAA_DISTANCE_MeanDriftRate | 153 | -0.15 | 5.6E-02 | 0.07 | 4.6E-01 | 0.27 | 0.25 | 0.02 |
| Task 2 | Prediction | MFGGLU_overall_MeanDriftRate | 146 | 0.01 | 7.9E-01 | 0.00 | 9.5E-01 | 0.71 | 0.71 | 0.00 |
| Task 2 | Prediction | MFGGLU_SNARC_MeanDriftRate | 149 | -0.10 | 2.4E-01 | -0.09 | 3.2E-01 | 0.15 | 0.15 | 0.00 |
| Task 2 | Prediction | MFGGLU_DISTANCE_MeanDriftRate | 150 | 0.11 | 2.6E-01 | -0.10 | 2.8E-01 | 0.30 | 0.30 | 0.00 |
| Task 2 | Prediction | MFGGABA_overall_MeanDriftRate | 142 | 0.09 | 4.8E-02 | -0.02 | 7.4E-01 | 0.72 | 0.72 | 0.00 |
| Task 2 | Prediction | MFGGABA_SNARC_MeanDriftRate | 143 | 0.06 | 4.9E-01 | -0.12 | 1.7E-01 | 0.15 | 0.16 | 0.00 |
| Task 2 | Prediction | MFGGABA_DISTANCE_MeanDriftRate | 146 | 0.03 | 6.7E-01 | 0.02 | 7.3E-01 | 0.25 | 0.26 | 0.00 |
| Task 2 | Prediction | MFGNAA_overall_MeanDriftRate | 145 | -0.08 | 7.6E-02 | -0.06 | 2.8E-01 | 0.72 | 0.71 | 0.00 |
| Task 2 | Prediction | MFGNAA_SNARC_MeanDriftRate | 147 | 0.12 | 1.1E-01 | 0.02 | 7.9E-01 | 0.15 | 0.14 | 0.00 |
| Task 2 | Prediction | MFGNAA_DISTANCE_MeanDriftRate | 150 | -0.18 | 1.7E-02 | -0.10 | 2.6E-01 | 0.29 | 0.27 | 0.02 |
| Task 2 | Prediction | IPSGLU_overall_BoundarySeparation | 144 | -0.15 | 1.4E-02 | 0.14 | 2.5E-02 | 0.67 | 0.66 | 0.01 |
| Task 2 | Prediction | IPSGLU_SNARC_BoundarySeparation | 150 | -0.02 | 8.1E-01 | -0.07 | 3.3E-01 | 0.59 | 0.59 | 0.00 |
| Task 2 | Prediction | IPSGLU_DISTANCE_BoundarySeparation | 151 | 0.18 | 4.0E-02 | -0.14 | 8.4E-02 | 0.29 | 0.27 | 0.02 |
| Task 2 | Prediction | IPSGABA_overall_BoundarySeparation | 145 | -0.09 | 1.5E-01 | 0.00 | 9.7E-01 | 0.62 | 0.62 | 0.00 |
| Task 2 | Prediction | IPSGABA_SNARC_BoundarySeparation | 150 | 0.04 | 5.5E-01 | -0.05 | 4.0E-01 | 0.58 | 0.58 | 0.00 |
| Task 2 | Prediction | IPSGABA_DISTANCE_BoundarySeparation | 151 | 0.04 | 6.1E-01 | 0.11 | 2.2E-01 | 0.26 | 0.27 | 0.00 |
| Task 2 | Prediction | IPSNAA_overall_BoundarySeparation | 146 | -0.08 | 2.1E-01 | 0.07 | 2.5E-01 | 0.63 | 0.63 | 0.00 |
| Task 2 | Prediction | IPSNAA_SNARC_BoundarySeparation | 151 | 0.00 | 9.6E-01 | -0.11 | 1.0E-01 | 0.59 | 0.59 | 0.00 |
| Task 2 | Prediction | IPSNAA_DISTANCE_BoundarySeparation | 152 | -0.01 | 9.0E-01 | 0.03 | 7.7E-01 | 0.25 | 0.26 | 0.00 |
| Task 2 | Prediction | MFGGLU_overall_BoundarySeparation | 143 | -0.12 | 5.7E-02 | 0.08 | 1.6E-01 | 0.69 | 0.68 | 0.01 |
| Task 2 | Prediction | MFGGLU_SNARC_BoundarySeparation | 149 | -0.06 | 3.4E-01 | -0.07 | 2.1E-01 | 0.58 | 0.58 | 0.00 |
| Task 2 | Prediction | MFGGLU_DISTANCE_BoundarySeparation | 150 | 0.08 | 3.2E-01 | -0.11 | 1.6E-01 | 0.27 | 0.27 | 0.00 |
| Task 2 | Prediction | MFGGABA_overall_BoundarySeparation | 140 | -0.10 | 1.0E-01 | 0.04 | 4.3E-01 | 0.69 | 0.69 | 0.01 |
| Task 2 | Prediction | MFGGABA_SNARC_BoundarySeparation | 144 | 0.00 | 9.8E-01 | 0.07 | 3.3E-01 | 0.58 | 0.58 | 0.00 |
| Task 2 | Prediction | MFGGABA_DISTANCE_BoundarySeparation | 145 | -0.07 | 4.0E-01 | -0.02 | 8.4E-01 | 0.27 | 0.27 | 0.00 |
| Task 2 | Prediction | MFGNAA_overall_BoundarySeparation | 142 | -0.06 | 2.8E-01 | 0.11 | 3.4E-02 | 0.69 | 0.69 | 0.00 |
| Task 2 | Prediction | MFGNAA_SNARC_BoundarySeparation | 148 | 0.00 | 9.6E-01 | -0.11 | 9.2E-02 | 0.58 | 0.59 | 0.00 |
| Task 2 | Prediction | MFGNAA_DISTANCE_BoundarySeparation | 149 | 0.01 | 9.2E-01 | -0.01 | 9.2E-01 | 0.26 | 0.26 | 0.00 |
| Task 2 | Prediction | IPSGLU_overall_NonDecisionTime | 144 | -0.10 | 1.6E-01 | 0.13 | 3.7E-02 | 0.77 | 0.76 | 0.01 |
| Task 2 | Prediction | IPSGLU_SNARC_NonDecisionTime | 150 | 0.06 | 3.5E-01 | -0.06 | 2.9E-01 | 0.57 | 0.57 | 0.00 |
| Task 2 | Prediction | IPSGLU_DISTANCE_NonDecisionTime | 149 | 0.28 | 5.5E-03 | -0.18 | 3.8E-02 | 0.43 | 0.38 | 0.05 |
| Task 2 | Prediction | IPSGABA_overall_NonDecisionTime | 142 | 0.15 | 1.1E-02 | 0.03 | 5.7E-01 | 0.79 | 0.78 | 0.01 |
| Task 2 | Prediction | IPSGABA_SNARC_NonDecisionTime | 150 | -0.01 | 9.4E-01 | 0.01 | 8.3E-01 | 0.56 | 0.56 | 0.00 |
| Task 2 | Prediction | IPSGABA_DISTANCE_NonDecisionTime | 148 | -0.01 | 9.1E-01 | 0.03 | 7.4E-01 | 0.40 | 0.41 | 0.00 |
| Task 2 | Prediction | IPSNAA_overall_NonDecisionTime | 144 | 0.07 | 2.1E-01 | 0.00 | 9.4E-01 | 0.79 | 0.79 | 0.00 |
| Task 2 | Prediction | IPSNAA_SNARC_NonDecisionTime | 151 | 0.00 | 9.8E-01 | -0.08 | 2.3E-01 | 0.56 | 0.57 | 0.00 |
| Task 2 | Prediction | IPSNAA_DISTANCE_NonDecisionTime | 150 | -0.02 | 8.0E-01 | 0.03 | 6.6E-01 | 0.40 | 0.41 | 0.00 |
| Task 2 | Prediction | MFGGLU_overall_NonDecisionTime | 143 | -0.02 | 7.7E-01 | 0.00 | 9.2E-01 | 0.77 | 0.77 | 0.00 |
| Task 2 | Prediction | MFGGLU_SNARC_NonDecisionTime | 149 | 0.01 | 8.8E-01 | -0.07 | 1.8E-01 | 0.55 | 0.56 | 0.00 |
| Task 2 | Prediction | MFGGLU_DISTANCE_NonDecisionTime | 148 | 0.17 | 3.8E-02 | -0.12 | 5.6E-02 | 0.44 | 0.43 | 0.01 |
| Task 2 | Prediction | MFGGABA_overall_NonDecisionTime | 139 | -0.01 | 8.5E-01 | 0.02 | 6.3E-01 | 0.79 | 0.79 | 0.00 |
| Task 2 | Prediction | MFGGABA_SNARC_NonDecisionTime | 144 | -0.08 | 3.1E-01 | 0.05 | 4.6E-01 | 0.55 | 0.55 | 0.00 |
| Task 2 | Prediction | MFGGABA_DISTANCE_NonDecisionTime | 143 | -0.02 | 8.1E-01 | 0.05 | 4.7E-01 | 0.41 | 0.41 | 0.00 |
| Task 2 | Prediction | MFGNAA_overall_NonDecisionTime | 142 | 0.08 | 1.7E-01 | 0.00 | 9.1E-01 | 0.79 | 0.79 | 0.00 |
| Task 2 | Prediction | MFGNAA_SNARC_NonDecisionTime | 148 | 0.04 | 6.2E-01 | -0.12 | 2.9E-02 | 0.56 | 0.56 | 0.00 |
| Task 2 | Prediction | MFGNAA_DISTANCE_NonDecisionTime | 147 | 0.01 | 9.1E-01 | -0.12 | 1.0E-01 | 0.41 | 0.42 | 0.00 |
| Task 3 | Prediction | IPSGLU_overall_MeanDriftRate | 138 | 0.01 | 8.3E-01 | 0.03 | 7.1E-01 | 0.69 | 0.69 | 0.00 |
| Task 3 | Prediction | IPSGLU_DISTANCE_MeanDriftRate | 137 | -0.04 | 6.4E-01 | -0.17 | 9.8E-02 | 0.11 | 0.12 | 0.00 |
| Task 3 | Prediction | IPSGABA_overall_MeanDriftRate | 138 | -0.05 | 3.7E-01 | 0.02 | 7.4E-01 | 0.71 | 0.71 | 0.00 |
| Task 3 | Prediction | IPSGABA_DISTANCE_MeanDriftRate | 138 | -0.06 | 4.3E-01 | -0.04 | 6.3E-01 | 0.10 | 0.10 | 0.00 |
| Task 3 | Prediction | IPSNAA_overall_MeanDriftRate | 138 | -0.08 | 1.2E-01 | -0.05 | 3.4E-01 | 0.72 | 0.71 | 0.00 |
| Task 3 | Prediction | IPSNAA_DISTANCE_MeanDriftRate | 138 | -0.08 | 3.4E-01 | -0.15 | 1.1E-01 | 0.12 | 0.12 | 0.00 |
| Task 3 | Prediction | MFGGLU_overall_MeanDriftRate | 135 | -0.15 | 1.2E-02 | -0.07 | 2.9E-01 | 0.71 | 0.70 | 0.01 |
| Task 3 | Prediction | MFGGLU_DISTANCE_MeanDriftRate | 135 | 0.01 | 9.6E-01 | -0.10 | 3.0E-01 | 0.10 | 0.10 | -0.01 |
| Task 3 | Prediction | MFGGABA_overall_MeanDriftRate | 131 | 0.02 | 7.6E-01 | 0.08 | 1.2E-01 | 0.69 | 0.69 | 0.00 |
| Task 3 | Prediction | MFGGABA_DISTANCE_MeanDriftRate | 130 | -0.03 | 7.4E-01 | 0.12 | 1.6E-01 | 0.11 | 0.11 | -0.01 |
| Task 3 | Prediction | MFGNAA_overall_MeanDriftRate | 135 | -0.11 | 3.1E-02 | -0.08 | 1.3E-01 | 0.69 | 0.68 | 0.01 |
| Task 3 | Prediction | MFGNAA_DISTANCE_MeanDriftRate | 134 | 0.04 | 6.5E-01 | 0.00 | 1.0E+00 | 0.08 | 0.08 | -0.01 |
| Task 3 | Prediction | IPSGLU_overall_BoundarySeparation | 138 | -0.10 | 1.8E-01 | 0.11 | 1.2E-01 | 0.60 | 0.59 | 0.01 |
| Task 3 | Prediction | IPSGLU_DISTANCE_BoundarySeparation | 137 | -0.05 | 2.8E-01 | 0.09 | 1.2E-01 | 0.68 | 0.68 | 0.00 |
| Task 3 | Prediction | IPSGABA_overall_BoundarySeparation | 138 | 0.10 | 1.6E-01 | -0.03 | 7.1E-01 | 0.57 | 0.57 | 0.00 |
| Task 3 | Prediction | IPSGABA_DISTANCE_BoundarySeparation | 138 | 0.10 | 9.7E-02 | 0.04 | 5.2E-01 | 0.68 | 0.67 | 0.01 |
| Task 3 | Prediction | IPSNAA_overall_BoundarySeparation | 139 | 0.01 | 8.8E-01 | 0.01 | 9.3E-01 | 0.58 | 0.59 | 0.00 |
| Task 3 | Prediction | IPSNAA_DISTANCE_BoundarySeparation | 137 | 0.12 | 2.9E-02 | -0.07 | 1.8E-01 | 0.67 | 0.66 | 0.01 |
| Task 3 | Prediction | MFGGLU_overall_BoundarySeparation | 136 | -0.15 | 2.5E-02 | 0.02 | 7.2E-01 | 0.60 | 0.59 | 0.01 |
| Task 3 | Prediction | MFGGLU_DISTANCE_BoundarySeparation | 134 | 0.06 | 3.4E-01 | -0.06 | 3.4E-01 | 0.65 | 0.65 | 0.00 |
| Task 3 | Prediction | MFGGABA_overall_BoundarySeparation | 131 | -0.03 | 6.9E-01 | 0.20 | 5.0E-04 | 0.62 | 0.62 | 0.00 |
| Task 3 | Prediction | MFGGABA_DISTANCE_BoundarySeparation | 130 | 0.02 | 7.4E-01 | -0.01 | 8.4E-01 | 0.65 | 0.65 | 0.00 |
| Task 3 | Prediction | MFGNAA_overall_BoundarySeparation | 135 | 0.03 | 6.3E-01 | 0.06 | 3.6E-01 | 0.58 | 0.58 | 0.00 |
| Task 3 | Prediction | MFGNAA_DISTANCE_BoundarySeparation | 133 | 0.08 | 1.7E-01 | -0.03 | 6.5E-01 | 0.65 | 0.65 | 0.00 |
| Task 3 | Prediction | IPSGLU_overall_NonDecisionTime | 135 | -0.10 | 5.3E-02 | 0.04 | 5.6E-01 | 0.62 | 0.61 | 0.00 |
| Task 3 | Prediction | IPSGLU_DISTANCE_NonDecisionTime | 137 | -0.05 | 4.1E-01 | 0.13 | 3.3E-02 | 0.69 | 0.69 | 0.00 |
| Task 3 | Prediction | IPSGABA_overall_NonDecisionTime | 136 | 0.23 | 2.6E-04 | -0.06 | 2.3E-01 | 0.66 | 0.62 | 0.04 |
| Task 3 | Prediction | IPSGABA_DISTANCE_NonDecisionTime | 137 | 0.14 | 1.9E-02 | 0.02 | 7.4E-01 | 0.71 | 0.69 | 0.02 |
| Task 3 | Prediction | IPSNAA_overall_NonDecisionTime | 137 | 0.11 | 1.3E-01 | -0.07 | 2.2E-01 | 0.64 | 0.64 | 0.01 |
| Task 3 | Prediction | IPSNAA_DISTANCE_NonDecisionTime | 137 | 0.12 | 3.4E-02 | 0.00 | 9.5E-01 | 0.70 | 0.69 | 0.01 |
| Task 3 | Prediction | MFGGLU_overall_NonDecisionTime | 134 | -0.07 | 4.2E-01 | -0.01 | 9.2E-01 | 0.65 | 0.65 | 0.00 |
| Task 3 | Prediction | MFGGLU_DISTANCE_NonDecisionTime | 135 | 0.13 | 8.6E-02 | -0.03 | 6.5E-01 | 0.67 | 0.66 | 0.01 |
| Task 3 | Prediction | MFGGABA_overall_NonDecisionTime | 128 | 0.04 | 6.6E-01 | -0.02 | 7.5E-01 | 0.63 | 0.63 | 0.00 |
| Task 3 | Prediction | MFGGABA_DISTANCE_NonDecisionTime | 129 | 0.09 | 1.7E-01 | -0.05 | 3.1E-01 | 0.68 | 0.68 | 0.00 |
| Task 3 | Prediction | MFGNAA_overall_NonDecisionTime | 132 | 0.12 | 1.6E-01 | -0.02 | 8.0E-01 | 0.64 | 0.63 | 0.01 |
| Task 3 | Prediction | MFGNAA_DISTANCE_NonDecisionTime | 133 | 0.12 | 6.8E-02 | -0.01 | 7.9E-01 | 0.68 | 0.67 | 0.01 |
