# Supplementary material for: Dissecting the chain of information processing and its interplay with neurochemicals and fluid intelligence across development
Source: eLife. 2023 Sep 29;12:e84086. doi: 10.7554/eLife.84086 (PMC10541179; doi:10.7554/eLife.84086)
Supplement: Supplementary file 7. — At the start of each entry, the dependent variable (Y), the independent variable (X), the mediator (M), and the moderator (W) are defined and highlighted in bold. 1. IPS GABA moderated mediation model with visuomotor connectivity as the mediator. 2. IPS glutamate moderated mediation model with visuomotor connectivity as the mediator. 3. IPS GABA moderated mediation model with visuomotor processing as the mediator. 4. IPS glutamate moderated mediation model with visuomotor processing as the mediator. [file elife-84086-supp7.docx]

**Supplementary File 7. Moderated mediation results. At the start of each entry, the dependent variable (Y), the independent variable (X), the mediator (M), and the moderator (W) are defined and highlighted in bold.**

**Supplementary File 7.1: IPS GABA moderated mediation model with visuomotor connectivity as the mediator**

***************** PROCESS Procedure for SPSS Version 4.1 *****************

Written by Andrew F. Hayes, Ph.D. www.afhayes.com

Documentation available in Hayes (2022). www.guilford.com/p/hayes3

**************************************************************************

Model : 59

**Y : Visuomotor processing**

**X : IPS GABA**

**M : Visuomotor connectivity**

**W : Age**

Sample

Size: 212

**************************************************************************

OUTCOME VARIABLE:

M

Model Summary

R R-sq MSE F df1 df2 p

.1485 .0220 .9921 1.5629 3.0000 208.0000 .1995

Model

coeff se t p LLCI ULCI

constant -.0375 .0713 -.5262 .5993 -.1553 .0803

X -.0616 .0717 -.8595 .3910 -.1800 .0568

age .1206 .0770 1.5661 .1188 -.0066 .2478

Int_1 .1306 .0697 1.8734 .0624 .0154 .2457

Product terms key:

Int_1 : X x age

Test(s) of highest order unconditional interaction(s):

R2-chng F df1 df2 p

X*W .0165 3.5097 1.0000 208.0000 .0624

----------

Focal predict: X (X)

Mod var: age (W)

Conditional effects of the focal predictor at values of the moderator(s):

age Effect se t p LLCI ULCI

-1.0000 -.1922 .1019 -1.8857 .0607 -.3605 -.0238

.0000 -.0616 .0717 -.8595 .3910 -.1800 .0568

1.0000 .0690 .0980 .7037 .4824 -.0929 .2309

**************************************************************************

OUTCOME VARIABLE:

DV

Model Summary

R R-sq MSE F df1 df2 p

.8159 .6656 .3425 82.0141 5.0000 206.0000 .0000

Model

coeff se t p LLCI ULCI

constant -.0616 .0422 -1.4585 .1462 -.1314 .0082

X -.1085 .0423 -2.5647 .0110 -.1785 -.0386

M .0185 .0409 .4508 .6526 -.0492 .0861

age -.6055 .0455 -13.3026 .0000 -.6807 -.5303

Int_1 .2383 .0428 5.5632 .0000 .1675 .3091

Int_2 -.1339 .0404 -3.3173 .0011 -.2006 -.0672

Product terms key:

Int_1 : X x age

Int_2 : M x age

Test(s) of highest order unconditional interaction(s):

R2-chng F df1 df2 p

X*W .0502 30.9492 1.0000 206.0000 .0000

M*W .0179 11.0044 1.0000 206.0000 .0011

----------

Focal predict: X (X)

Mod var: age (W)

Conditional effects of the focal predictor at values of the moderator(s):

age Effect se t p LLCI ULCI

-1.0000 -.3468 .0622 -5.5797 .0000 -.4495 -.2441

.0000 -.1085 .0423 -2.5647 .0110 -.1785 -.0386

1.0000 .1298 .0582 2.2294 .0269 .0336 .2259

----------

Focal predict: M (M)

Mod var: age (W)

Conditional effects of the focal predictor at values of the moderator(s):

age Effect se t p LLCI ULCI

-1.0000 .1524 .0603 2.5259 .0123 .0527 .2520

.0000 .0185 .0409 .4508 .6526 -.0492 .0861

1.0000 -.1154 .0545 -2.1170 .0355 -.2055 -.0253

****************** DIRECT AND INDIRECT EFFECTS OF X ON Y *****************

Conditional direct effect(s) of X on Y:

age Effect se t p LLCI ULCI

-1.0000 -.3468 .0622 -5.5797 .0000 -.4495 -.2441

.0000 -.1085 .0423 -2.5647 .0110 -.1785 -.0386

1.0000 .1298 .0582 2.2294 .0269 .0336 .2259

Conditional indirect effects of X on Y:

INDIRECT EFFECT:

X -> M -> DV

age Effect BootSE BootLLCI BootULCI

-1.0000 -.0293 .0199 -.0632 -.0007

.0000 -.0011 .0040 -.0087 .0043

1.0000 -.0080 .0127 -.0320 .0089

---

*********************** ANALYSIS NOTES AND ERRORS ************************

Level of confidence for all confidence intervals in output:

90.0000

Number of bootstrap samples for percentile bootstrap confidence intervals:

5000

W values in conditional tables are the mean and +/- SD from the mean.

NOTE: The following variables were mean centered prior to analysis:

age X M

------ END MATRIX -----

**Supplementary File 7.2: IPS glutamate moderated mediation model with visuomotor connectivity as the mediator**

***************** PROCESS Procedure for SPSS Version 4.1 *****************

Written by Andrew F. Hayes, Ph.D. www.afhayes.com

Documentation available in Hayes (2022). www.guilford.com/p/hayes3

**************************************************************************

Model : 59

**Y : Visuomotor processing**

**X : IPS glutamate**

**M : Visuomotor connectivity**

**W : Age**

Sample

Size: 210

**************************************************************************

OUTCOME VARIABLE:

M

Model Summary

R R-sq MSE F df1 df2 p

.1298 .0168 .9975 1.1758 3.0000 206.0000 .3200

Model

coeff se t p LLCI ULCI

constant -.0384 .0778 -.4934 .6223 -.1670 .0902

X -.0963 .0788 -1.2215 .2233 -.2265 .0339

age .0612 .0881 .6946 .4881 -.0843 .2067

Int_1 -.0802 .0754 -1.0629 .2891 -.2048 .0445

Product terms key:

Int_1 : X x age

Test(s) of highest order unconditional interaction(s):

R2-chng F df1 df2 p

X*W .0054 1.1298 1.0000 206.0000 .2891

**************************************************************************

OUTCOME VARIABLE:

DV

Model Summary

R R-sq MSE F df1 df2 p

.7779 .6052 .4045 62.5398 5.0000 204.0000 .0000

Model

coeff se t p LLCI ULCI

constant -.0557 .0501 -1.1115 .2677 -.1386 .0271

X .0234 .0504 .4652 .6423 -.0598 .1067

M .0321 .0445 .7218 .4712 -.0414 .1057

age -.6448 .0562 -11.4818 .0000 -.7376 -.5520

Int_1 -.1375 .0491 -2.7986 .0056 -.2187 -.0563

Int_2 -.1548 .0432 -3.5868 .0004 -.2261 -.0835

Product terms key:

Int_1 : X x age

Int_2 : M x age

Test(s) of highest order unconditional interaction(s):

R2-chng F df1 df2 p

X*W .0152 7.8319 1.0000 204.0000 .0056

M*W .0249 12.8654 1.0000 204.0000 .0004

----------

Focal predict: X (X)

Mod var: age (W)

Conditional effects of the focal predictor at values of the moderator(s):

age Effect se t p LLCI ULCI

-1.0000 .1610 .0699 2.3017 .0224 .0454 .2766

.0000 .0234 .0504 .4652 .6423 -.0598 .1067

1.0000 -.1141 .0708 -1.6106 .1088 -.2311 .0030

----------

Focal predict: M (M)

Mod var: age (W)

Conditional effects of the focal predictor at values of the moderator(s):

age Effect se t p LLCI ULCI

-1.0000 .1869 .0644 2.9005 .0041 .0804 .2934

.0000 .0321 .0445 .7218 .4712 -.0414 .1057

1.0000 -.1227 .0594 -2.0632 .0404 -.2209 -.0244

****************** DIRECT AND INDIRECT EFFECTS OF X ON Y *****************

Conditional direct effect(s) of X on Y:

age Effect se t p LLCI ULCI

-1.0000 .1610 .0699 2.3017 .0224 .0454 .2766

.0000 .0234 .0504 .4652 .6423 -.0598 .1067

1.0000 -.1141 .0708 -1.6106 .1088 -.2311 .0030

Conditional indirect effects of X on Y:

INDIRECT EFFECT:

X -> M -> DV

age Effect BootSE BootLLCI BootULCI

-1.0000 -.0030 .0221 -.0428 .0296

.0000 -.0031 .0062 -.0147 .0051

1.0000 .0216 .0197 -.0034 .0590

---

*********************** ANALYSIS NOTES AND ERRORS ************************

Level of confidence for all confidence intervals in output:

90.0000

Number of bootstrap samples for percentile bootstrap confidence intervals:

5000

W values in conditional tables are the mean and +/- SD from the mean.

NOTE: The following variables were mean centered prior to analysis:

age X M

------ END MATRIX -----

**Supplementary File 7.3: IPS GABA moderated mediation model with visuomotor processing as the mediator**

***************** PROCESS Procedure for SPSS Version 4.1 *****************

Written by Andrew F. Hayes, Ph.D. www.afhayes.com

Documentation available in Hayes (2022). www.guilford.com/p/hayes3

**************************************************************************

Model : 59

**Y : Intelligence (matrix reasoning)**

**X : IPS GABA**

**M : Visuomotor processing**

**W : Age**

Sample

Size: 224

**************************************************************************

OUTCOME VARIABLE:

M

Model Summary

R R-sq MSE F df1 df2 p

.8102 .6564 .3483 140.0732 3.0000 220.0000 .0000

Model

coeff se t p LLCI ULCI

constant -.0842 .0421 -2.0002 .0467 -.1538 -.0147

X -.1235 .0424 -2.9156 .0039 -.1935 -.0535

age -.6298 .0462 -13.6239 .0000 -.7062 -.5535

Int_1 .2437 .0427 5.7092 .0000 .1732 .3142

Product terms key:

Int_1 : X x age

Test(s) of highest order unconditional interaction(s):

R2-chng F df1 df2 p

X*W .0509 32.5951 1.0000 220.0000 .0000

----------

Focal predict: X (X)

Mod var: age (W)

Conditional effects of the focal predictor at values of the moderator(s):

age Effect se t p LLCI ULCI

-1.0000 -.3672 .0631 -5.8152 .0000 -.4715 -.2629

.0000 -.1235 .0424 -2.9156 .0039 -.1935 -.0535

1.0000 .1202 .0570 2.1091 .0361 .0261 .2143

**************************************************************************

OUTCOME VARIABLE:

DV

Model Summary

R R-sq MSE F df1 df2 p

.7578 .5742 .4355 58.8073 5.0000 218.0000 .0000

Model

coeff se t p LLCI ULCI

constant .1268 .0601 2.1088 .0361 .0275 .2262

X -.0122 .0486 -.2509 .8022 -.0925 .0681

M .1506 .1104 1.3633 .1742 -.0319 .3330

age .7022 .0724 9.6976 .0000 .5826 .8218

Int_1 .0233 .0542 .4301 .6675 -.0662 .1128

Int_2 .1754 .0560 3.1320 .0020 .0829 .2679

Product terms key:

Int_1 : X x age

Int_2 : M x age

Test(s) of highest order unconditional interaction(s):

R2-chng F df1 df2 p

X*W .0004 .1850 1.0000 218.0000 .6675

M*W .0192 9.8097 1.0000 218.0000 .0020

----------

Focal predict: M (M)

Mod var: age (W)

Conditional effects of the focal predictor at values of the moderator(s):

age Effect se t p LLCI ULCI

-1.0000 -.0248 .0793 -.3126 .7549 -.1559 .1063

.0000 .1506 .1104 1.3633 .1742 -.0319 .3330

1.0000 .3259 .1561 2.0878 .0380 .0681 .5838

****************** DIRECT AND INDIRECT EFFECTS OF X ON Y *****************

Conditional direct effect(s) of X on Y:

age Effect se t p LLCI ULCI

-1.0000 -.0355 .0794 -.4472 .6552 -.1666 .0956

.0000 -.0122 .0486 -.2509 .8022 -.0925 .0681

1.0000 .0111 .0655 .1696 .8655 -.0971 .1194

Conditional indirect effects of X on Y:

INDIRECT EFFECT:

X -> M -> DV

age Effect BootSE BootLLCI BootULCI

-1.0000 .0091 .0312 -.0419 .0621

.0000 -.0186 .0168 -.0494 .0044

1.0000 .0392 .0263 .0017 .0861

---

*********************** ANALYSIS NOTES AND ERRORS ************************

Level of confidence for all confidence intervals in output:

90.0000

Number of bootstrap samples for percentile bootstrap confidence intervals:

5000

W values in conditional tables are the mean and +/- SD from the mean.

NOTE: The following variables were mean centered prior to analysis:

age X M

------ END MATRIX -----

**Supplementary File 7.4: IPS glutamate moderated mediation model with visuomotor processing as the mediator**

***************** PROCESS Procedure for SPSS Version 4.1 *****************

Written by Andrew F. Hayes, Ph.D. www.afhayes.com

Documentation available in Hayes (2022). www.guilford.com/p/hayes3

**************************************************************************

Model : 59

**Y : Intelligence (matrix reasoning)**

**X : IPS glutamate**

**M : Visuomotor processing**

**W : Age**

Sample

Size: 223

**************************************************************************

OUTCOME VARIABLE:

M

Model Summary

R R-sq MSE F df1 df2 p

.7995 .6391 .3658 129.2917 3.0000 219.0000 .0000

Model

coeff se t p LLCI ULCI

constant -.1166 .0467 -2.4990 .0132 -.1937 -.0395

X .0361 .0474 .7627 .4465 -.0421 .1144

age -.6483 .0520 -12.4747 .0000 -.7342 -.5625

Int_1 -.2274 .0452 -5.0321 .0000 -.3021 -.1528

Product terms key:

Int_1 : X x age

Test(s) of highest order unconditional interaction(s):

R2-chng F df1 df2 p

X*W .0417 25.3216 1.0000 219.0000 .0000

----------

Focal predict: X (X)

Mod var: age (W)

Conditional effects of the focal predictor at values of the moderator(s):

age Effect se t p LLCI ULCI

-1.0000 .2636 .0661 3.9892 .0001 .1544 .3727

.0000 .0361 .0474 .7627 .4465 -.0421 .1144

1.0000 -.1913 .0649 -2.9492 .0035 -.2984 -.0842

**************************************************************************

OUTCOME VARIABLE:

DV

Model Summary

R R-sq MSE F df1 df2 p

.7594 .5767 .4331 59.1211 5.0000 217.0000 .0000

Model

coeff se t p LLCI ULCI

constant .1489 .0622 2.3937 .0175 .0461 .2516

X -.0128 .0516 -.2487 .8038 -.0981 .0724

M .1525 .1102 1.3846 .1676 -.0294 .3345

age .6783 .0762 8.9038 .0000 .5524 .8041

Int_1 .0669 .0538 1.2439 .2149 -.0220 .1558

Int_2 .1490 .0547 2.7210 .0070 .0585 .2394

Product terms key:

Int_1 : X x age

Int_2 : M x age

Test(s) of highest order unconditional interaction(s):

R2-chng F df1 df2 p

X*W .0030 1.5474 1.0000 217.0000 .2149

M*W .0144 7.4040 1.0000 217.0000 .0070

----------

Focal predict: M (M)

Mod var: age (W)

Conditional effects of the focal predictor at values of the moderator(s):

age Effect se t p LLCI ULCI

-1.0000 .0036 .0784 .0453 .9639 -.1260 .1331

.0000 .1525 .1102 1.3846 .1676 -.0294 .3345

1.0000 .3015 .1553 1.9415 .0535 .0450 .5580

****************** DIRECT AND INDIRECT EFFECTS OF X ON Y *****************

Conditional direct effect(s) of X on Y:

age Effect se t p LLCI ULCI

-1.0000 -.0798 .0760 -1.0494 .2952 -.2054 .0458

.0000 -.0128 .0516 -.2487 .8038 -.0981 .0724

1.0000 .0541 .0731 .7402 .4600 -.0666 .1748

Conditional indirect effects of X on Y:

INDIRECT EFFECT:

X -> M -> DV

age Effect BootSE BootLLCI BootULCI

-1.0000 .0009 .0229 -.0346 .0403

.0000 .0055 .0090 -.0059 .0227

1.0000 -.0577 .0356 -.1210 -.0061

---

*********************** ANALYSIS NOTES AND ERRORS ************************

Level of confidence for all confidence intervals in output:

90.0000

Number of bootstrap samples for percentile bootstrap confidence intervals:

5000

W values in conditional tables are the mean and +/- SD from the mean.

NOTE: The following variables were mean centered prior to analysis:

age X M

------ END MATRIX -----
