# Supplementary material for: Dissecting the chain of information processing and its interplay with neurochemicals and fluid intelligence across development
Source: eLife. 2023 Sep 29;12:e84086. doi: 10.7554/eLife.84086 (PMC10541179; doi:10.7554/eLife.84086)
Supplement: Supplementary file 8. [file elife-84086-supp8.docx]

**Supplementary File 8: Age moderated the relationship between the diffusion parameter and fluid intelligence (CI_L= 95% lower bound confidence interval, CI_U= 95% upper bound confidence interval). We did not control for the other two diffusion parameters in these analyses.**

|  | **Effect** | **t** | **p** | **CI_L** | **CI_U** |
| --- | --- | --- | --- | --- | --- |
| **Mean Drift Rate** | | | | | |
| **Younger** | .4051 | 4.2816 | .0000 | .2187 | .5915 |
| **Mean** | .2515 | 2.8828 | .0043 | .0796 | .4234 |
| **Older** | .0979 | .9610 | .3375 | -.1028 | .2985 |
| **Decision Boundary** | | | | | |
| **Younger** | -.2198 | -3.0673 | .0024 | -.3609 | -.0786 |
| **Mean** | -.1247 | -1.5728 | .1171 | -.2810 | .0315 |
| **Older** | -.0297 | -.2789 | .7806 | -.2395 | .1801 |
| **Non-Decision Time** | | | | | |
| **Younger** | -.0604 | -.8268 | .4092 | -.2045 | .0836 |
| **Mean** | .1324 | 1.2604 | .2088 | -.0745 | .3393 |
| **Older** | .3252 | 2.1848 | .0299 | .0320 | .6184 |
