# Supplementary material for: Dissecting the chain of information processing and its interplay with neurochemicals and fluid intelligence across development
Source: eLife. 2023 Sep 29;12:e84086. doi: 10.7554/eLife.84086 (PMC10541179; doi:10.7554/eLife.84086)
Supplement: Supplementary file 9. [file elife-84086-supp9.docx]

**Supplementary File 9: Mean accuracy, mean reaction time (RT) and associated standard deviations (SD) in each of the three tasks (indicated in the first column).**

| **Task** | **Variable** | **mean** | **SD** |
| --- | --- | --- | --- |
| **Task 1** | **accuracy** | 0.98 | 0.03 |
| **Task 1** | **RT** | 549.37 | 192.11 |
| **Task 2** | **accuracy** | 0.94 | 0.05 |
| **Task 2** | **RT** | 651.22 | 339.99 |
| **Task 3** | **accuracy** | 0.94 | 0.07 |
| **Task 3** | **RT** | 1481.96 | 849.82 |
