# Supplementary material for: Dissecting the chain of information processing and its interplay with neurochemicals and fluid intelligence across development
Source: eLife. 2023 Sep 29;12:e84086. doi: 10.7554/eLife.84086 (PMC10541179; doi:10.7554/eLife.84086)
Supplement: Supplementary file 10. — As expected, positive correlations of the three diffusion parameters were obtained across the three different tasks assessing attention (Task 1, ANT), digit comparison (Task 2, DC) and mental rotation (Task 3, MRT). [file elife-84086-supp10.docx]

**Supplementary File 10. Correlations between the diffusion parameters (v=mean drift rate, a=boundary separation, Ter=non-decision time) within and between tasks.** As expected, positive correlations of the three diffusion parameters were obtained across the three different tasks assessing attention (**Task 1**, ANT), digit comparison (**Task 2**, DC) and mental rotation (**Task 3**, MRT).

|  | **ANT_v** | **DC_v** | **MRT_v** | **ANT_a** | **DC_a** | **MRT_a** | **ANT_Ter** | **DC_Ter** | **MRT_Ter** |
| --- | --- | --- | --- | --- | --- | --- | --- | --- | --- |
| **ANT_v** | 1 | .715** | .600** | -.718** | -.570** | -.440** | -.583** | -.610** | -.341** |
| **DC_v** |  | 1 | .663** | -.661** | -.621** | -.394** | -.614** | -.675** | -.324** |
| **MRT_v** |  |  | 1 | -.609** | -.525** | -.574** | -.517** | -.571** | -.357** |
| **ANT_a** |  |  |  | 1 | .688** | .609** | .626** | .655** | .285** |
| **DC_a** |  |  |  |  | 1 | .598** | .703** | .711** | .330** |
| **MRT_a** |  |  |  |  |  | 1 | .516** | .552** | .164** |
| **ANT_Ter** |  |  |  |  |  |  | 1 | .738** | .423** |
| **DC_Ter** |  |  |  |  |  |  |  | 1 | .443** |
| **MRT_Ter** |  |  |  |  |  |  |  |  | 1 |
