# Supplementary material for: Dissecting the chain of information processing and its interplay with neurochemicals and fluid intelligence across development
Source: eLife. 2023 Sep 29;12:e84086. doi: 10.7554/eLife.84086 (PMC10541179; doi:10.7554/eLife.84086)
Supplement: Supplementary file 11. [file elife-84086-supp11.docx]

**Supplementary File 11**. Statistical results using the same statistical model as in **Supplementary File 3** (multiple linear regressions with bootstrapping predicting overall visuomotor processing during the first and the second assessment) but using the raw neurochemical concentration values (A1: first assessment, A2: second assessment, β=the regression coefficient of the variable listed in the “Effect” column, df=degrees of freedom, T=t-statistic, P_B_=Bootstrapped P-value, CI_L=lower bound of the confidence intervals obtained from bootstrapping, CI_U=upper bound of the confidence intervals obtained from bootstrapping) for **Task 1** (Attention network task, top third), **Task 2** (Digit comparison task, middle third), and **Task 3** (Mental rotation task, bottom third).

| **Assessment** | **Task** | **Effect** | **df** | **β** | **T** | **CI_L** | **CI_U** | **P_B_** |
| --- | --- | --- | --- | --- | --- | --- | --- | --- |
| A1 | Task 1 | IPS Glutamate*age | 252 | -0.29 | -6.96 | -0.40 | -0.18 | 0.00000 |
| A1 | Task 1 | IPS GABA*age | 251 | 0.23 | 5.87 | 0.14 | 0.32 | 0.00000 |
| A2 | Task 1 | IPS Glutamate*age | 174 | -0.35 | -6.60 | -0.46 | -0.24 | 0.00000 |
| A2 | Task 1 | IPS GABA*age | 174 | 0.19 | 3.70 | 0.07 | 0.35 | 0.00662 |
| A1 | Task 2 | IPS Glutamate*age | 240 | -0.24 | -5.82 | -0.35 | -0.14 | 0.00001 |
| A1 | Task 2 | IPS GABA*age | 240 | 0.18 | 5.15 | 0.08 | 0.26 | 0.00013 |
| A2 | Task 2 | IPS Glutamate*age | 170 | -0.24 | -4.86 | -0.36 | -0.12 | 0.00006 |
| A2 | Task 2 | IPS GABA*age | 169 | 0.26 | 5.48 | 0.15 | 0.37 | 0.00000 |
| A1 | Task 3 | IPS Glutamate*age | 222 | -0.25 | -4.74 | -0.36 | -0.13 | 0.00004 |
| A1 | Task 3 | IPS GABA*age | 224 | 0.23 | 4.06 | 0.06 | 0.39 | 0.00606 |
| A2 | Task 3 | IPS Glutamate*age | 165 | -0.03 | -0.37 | -0.20 | 0.15 | 0.77909 |
| A2 | Task 3 | IPS GABA*age | 166 | 0.32 | 4.55 | 0.15 | 0.49 | 0.00020 |
