# Supplementary material for: Dissecting the chain of information processing and its interplay with neurochemicals and fluid intelligence across development
Source: eLife. 2023 Sep 29;12:e84086. doi: 10.7554/eLife.84086 (PMC10541179; doi:10.7554/eLife.84086)
Supplement: Supplementary file 12. [file elife-84086-supp12.docx]

**Supplementary File 12**. Statistical results using the same statistical model as in **Supplementary File 3** (multiple linear regressions with bootstrapping predicting overall visuomotor processing during the first and the second assessment) but using the relative to total creatine neurochemical concentration values (A1: first assessment, A2: second assessment, β=the regression coefficient of the variable listed in the “Effect” column, df=degrees of freedom, T=t-statistic, P_B_=Bootstrapped P-value, CI_L=lower bound of the confidence intervals obtained from bootstrapping, CI_U=upper bound of the confidence intervals obtained from bootstrapping) for **Task 1** (Attention network task, top third), **Task 2** (Digit comparison task, middle third), and **Task 3** (Mental rotation task, bottom third).

| **Assessment** | **Task** | **Effect** | **df** | **β** | **T** | **CI_L** | **CI_U** | **P_B_** |
| --- | --- | --- | --- | --- | --- | --- | --- | --- |
| A1 | Task 1 | IPS Glutamate*age | 253 | -0.31 | -8.40 | -0.40 | -0.22 | 0.00000 |
| A1 | Task 1 | IPS GABA*age | 251 | 0.17 | 4.14 | 0.07 | 0.28 | 0.00181 |
| A2 | Task 1 | IPS Glutamate*age | 172 | -0.30 | -5.72 | -0.40 | -0.20 | 0.00000 |
| A2 | Task 1 | IPS GABA*age | 175 | 0.20 | 3.69 | 0.07 | 0.36 | 0.00704 |
| A1 | Task 2 | IPS Glutamate*age | 243 | -0.22 | -5.78 | -0.33 | -0.14 | 0.00000 |
| A1 | Task 2 | IPS GABA*age | 239 | 0.14 | 4.02 | 0.03 | 0.23 | 0.00381 |
| A2 | Task 2 | IPS Glutamate*age | 168 | -0.24 | -4.90 | -0.36 | -0.13 | 0.00004 |
| A2 | Task 2 | IPS GABA*age | 169 | 0.25 | 5.05 | 0.12 | 0.37 | 0.00009 |
| A1 | Task 3 | IPS Glutamate*age | 223 | -0.22 | -4.53 | -0.32 | -0.10 | 0.00025 |
| A1 | Task 3 | IPS GABA*age | 223 | 0.18 | 3.18 | 0.01 | 0.34 | 0.02579 |
| A2 | Task 3 | IPS Glutamate*age | 164 | -0.13 | -1.85 | -0.29 | 0.04 | 0.13628 |
| A2 | Task 3 | IPS GABA*age | 166 | 0.28 | 3.80 | 0.11 | 0.44 | 0.00116 |
