# Supplementary material for: Dissecting the chain of information processing and its interplay with neurochemicals and fluid intelligence across development
Source: eLife. 2023 Sep 29;12:e84086. doi: 10.7554/eLife.84086 (PMC10541179; doi:10.7554/eLife.84086)
Supplement: Supplementary file 13. [file elife-84086-supp13.docx]

**Supplementary File 13**. Statistical results using the same statistical model as in **Supplementary File 3** (multiple linear regressions with bootstrapping predicting overall visuomotor processing during the first and the second assessment) but using the T2 corrected (Equation 2) neurochemical concentration values (A1: first assessment, A2: second assessment, β=the regression coefficient of the variable listed in the “Effect” column, df=degrees of freedom, T=t-statistic, P_B_=Bootstrapped P-value, CI_L=lower bound of the confidence intervals obtained from bootstrapping, CI_U=upper bound of the confidence intervals obtained from bootstrapping) for **Task 1** (Attention network task, top third), **Task 2** (Digit comparison task, middle third), and **Task 3** (Mental rotation task, bottom third).

| **Assessment** | **Task** | **Effect** | **df** | **β** | **T** | **CI_L** | **CI_U** | **P_B_** |
| --- | --- | --- | --- | --- | --- | --- | --- | --- |
| A1 | Task 1 | IPS Glutamate*age | 252 | -0.25 | -6.45 | -0.34 | -0.15 | 0.00000 |
| A1 | Task 1 | IPS GABA*age | 252 | 0.19 | 4.81 | 0.10 | 0.28 | 0.00003 |
| A2 | Task 1 | IPS Glutamate*age | 174 | -0.28 | -5.26 | -0.39 | -0.16 | 0.00000 |
| A2 | Task 1 | IPS GABA*age | 175 | 0.21 | 3.80 | 0.08 | 0.38 | 0.00531 |
| A1 | Task 2 | IPS Glutamate*age | 240 | -0.19 | -5.10 | -0.29 | -0.11 | 0.00001 |
| A1 | Task 2 | IPS GABA*age | 240 | 0.16 | 4.41 | 0.05 | 0.25 | 0.00193 |
| A2 | Task 2 | IPS Glutamate*age | 169 | -0.19 | -4.03 | -0.31 | -0.07 | 0.00159 |
| A2 | Task 2 | IPS GABA*age | 168 | 0.27 | 5.43 | 0.15 | 0.38 | 0.00001 |
| A1 | Task 3 | IPS Glutamate*age | 222 | -0.26 | -5.16 | -0.40 | -0.12 | 0.00035 |
| A1 | Task 3 | IPS GABA*age | 224 | 0.25 | 4.27 | 0.08 | 0.42 | 0.00359 |
| A2 | Task 3 | IPS Glutamate*age | 164 | 0.09 | 1.29 | -0.07 | 0.27 | 0.31662 |
| A2 | Task 3 | IPS GABA*age | 166 | 0.39 | 5.43 | 0.22 | 0.57 | 0.00001 |
