# Supplementary material for: Dissecting the chain of information processing and its interplay with neurochemicals and fluid intelligence across development
Source: eLife. 2023 Sep 29;12:e84086. doi: 10.7554/eLife.84086 (PMC10541179; doi:10.7554/eLife.84086)
Supplement: Supplementary file 14. — We conducted exploratory factor analyses by adding as input the non-decision time parameters of the three tasks (i.e., three variables as input) after controlling for age, and only one factor was extracted (i.e., eigenvalue>1). As can be seen in the table below, this factor (extraction method: Principal Component Analysis, rotation method: none) was consistently positively related to the non-decision time (Ter) of each of the three tasks. [file elife-84086-supp14.docx]

**Supplementary File 14. Complementary Factor Analysis results.** We conducted exploratory factor analyses by adding as input the non-decision time parameters of the three tasks (i.e., three variables as input) after controlling for age, and only one factor was extracted (i.e., eigenvalue>1). As can be seen in the table below, this factor (extraction method: Principal Component Analysis, rotation method: none) was consistently positively related to the non-decision time (Ter) of each of the three tasks.

| **Component Matrix** | |
| --- | --- |
| Task_1_Ter | 0.824 |
| Task_2_Ter | 0.872 |
| Task_3_Ter | 0.497 |

Subsequently, we found a significant interaction between age and neurochemical measures in tracking this non-decision time factor derived from factor analysis: IPS glutamate*age (β=-.28, t(218)=-4.37, P_BO_<.001, CI=[-.42, -.15]) and IPS GABA*age: (β=.37, t(220)=6.11, P_BO_<.001, CI=[.19, .51]) replicating the results in the main text.
