# Supplementary material for: Dissecting the chain of information processing and its interplay with neurochemicals and fluid intelligence across development
Source: eLife. 2023 Sep 29;12:e84086. doi: 10.7554/eLife.84086 (PMC10541179; doi:10.7554/eLife.84086)
Supplement: Supplementary file 15. — These additional analyses support that both predictors (i.e., IPS glutamate*age and IPS GABA*age) were independently significant in tracking task performance (non-decision time in Task 1, Task 2 and when Task 1-3 were combined) even when both were added in the same multiple regression model. [file elife-84086-supp15.docx]

**Supplementary File 15**. Statistical results of multiple linear regressions with bootstrapping during the first assessment (β=the regression coefficient of the variable listed in the “Effect” column, P_B_=Bootstrapped P-value, CI_L=lower bound of the confidence intervals obtained from bootstrapping, CI_U=upper bound of the confidence intervals obtained from bootstrapping). These additional analyses support that both predictors (i.e., IPS glutamate*age and IPS GABA*age) were independently significant in tracking task performance (non-decision time in Task 1, Task 2 and when Task 1-3 were combined) even when both were added in the same multiple regression model.

| Outcome: non-decision time of Task 1 | | | | |
| --- | --- | --- | --- | --- |
| Effect | β | P_B_ | CI_L | CI_U |
| Glutamate * age | -0.2 | <.001 | -0.29 | -0.11 |
| GABA * age | 0.25 | <.001 | 0.17 | 0.34 |
| age | -0.58 | <.001 | -0.7 | -0.45 |
| Glutamate | 0 | 0.995 | -0.1 | 0.09 |
| GABA | -0.09 | 0.032 | -0.18 | -0.01 |
| Outcome: non-decision time of Task 2 | | | | |
| Effect | β | P_B_ | CI_L | CI_U |
| Glutamate * age | -0.2 | <.001 | -0.29 | -0.12 |
| GABA * age | 0.21 | <.001 | 0.12 | 0.28 |
| age | -0.61 | <.001 | -0.7 | -0.52 |
| Glutamate | 0.02 | 0.6 | -0.06 | 0.09 |
| GABA | -0.09 | 0.025 | -0.17 | -0.01 |
| Outcome: non-decision time of Task 3 | | | | |
| Effect | β | P_B_ | CI_L | CI_U |
| Glutamate * age | -0.14 | 0.062 | -0.29 | 0.02 |
| GABA * age | 0.18 | 0.033 | 0.01 | 0.36 |
| age | -0.3 | 0.004 | -0.49 | -0.1 |
| Glutamate | 0.13 | 0.05 | 0 | 0.26 |
| GABA | -0.13 | 0.063 | -0.28 | 0 |
| Outcome: averaged z-scored non-decision time of Tasks 1-3 | | | | |
| Effect | β | P_B_ | CI_L | CI_U |
| Glutamate * age | -0.23 | <.001 | -0.33 | -0.13 |
| GABA * age | 0.24 | <.001 | 0.13 | 0.34 |
| Age | -0.49 | <.001 | -0.63 | -0.35 |
| Glutamate | 0.07 | 0.147 | -0.03 | 0.16 |
| GABA | -0.11 | 0.019 | -0.2 | -0.02 |
| Outcome: intelligence (matrix reasoning) | | | | |
| Effect | β | P_B_ | CI_L | CI_U |
| Glutamate * age | 0.17 | 0.001 | 0.06 | 0.25 |
| GABA * age | -0.06 | 0.174 | -0.14 | 0.03 |
| Age | 0.65 | <.001 | 0.54 | 0.78 |
| Glutamate | -0.05 | 0.268 | -0.15 | 0.05 |
| GABA | 0.02 | 0.633 | -0.07 | 0.1 |
