# Supplementary material for: Dissecting the chain of information processing and its interplay with neurochemicals and fluid intelligence across development
Source: eLife. 2023 Sep 29;12:e84086. doi: 10.7554/eLife.84086 (PMC10541179; doi:10.7554/eLife.84086)
Supplement: Supplementary file 16. [file elife-84086-supp16.docx]

**Supplementary File 16.** To address the possibility that non-linear effects of age could impact the main analyses reported in the main text, we conducted additional analyses where we compared the model in SF16-eq1 to the model in SF16-eq2 using an R-change ANOVA test, which was significant.

Non-decision time~ IPS glutamate + IPS GABA + age + age^2 + age^3

SF16-eq1

Non-decision time~ IPS glutamate + IPS GABA + age + age^2 + age^3 + IPS glutamate*age + IPS GABA*age

SF16-eq2

As can be seen in the table below, the addition of the interaction terms (IPS glutamate*age and IPS GABA*age) significantly improved the R Square change.

| Model | R Square | Adjusted R Square | SE | R Square Change | F Change | df1 | df2 | Sig. F Change |
| --- | --- | --- | --- | --- | --- | --- | --- | --- |
| *SF16-eq1* | 0.719 | 0.713 | 0.42593 | 0.719 | 112.26 | 5 | 219 | <.001 |
| *SF16-eq2* | 0.734 | 0.726 | 0.41639 | 0.015 | 6.078 | 2 | 217 | 0.003 |

Moreover, as can be seen in the table below (SF16-eq2), both the IPS glutamate*age and IPS GABA*age predictors were still significant even after controlling for the linear and non-linear age terms.

|  | **β** | **T** | **P** |
| --- | --- | --- | --- |
| age | -0.337 | -4.558 | <.001 |
| IPS glutamate | 0.06 | 1.36 | 0.175 |
| IPS GABA | -0.075 | -1.818 | 0.07 |
| age^2 | 0.151 | 2.358 | 0.019 |
| age^3 | -0.052 | -1.569 | 0.118 |
| IPS glutamate*age | -0.133 | -3.007 | 0.003 |
| IPS GABA*age | 0.118 | 2.608 | 0.01 |
