# Supplementary material for: Dissecting the chain of information processing and its interplay with neurochemicals and fluid intelligence across development
Source: eLife. 2023 Sep 29;12:e84086. doi: 10.7554/eLife.84086 (PMC10541179; doi:10.7554/eLife.84086)
Supplement: Source data 1. [file elife-84086-data1.zip › DataDescription.docx]

There are 23 .csv files containing the data underlying Figures 2-7. In the tables below, there is information regarding the variables of each column of each .csv file (e.g., the column “age” refers to the age of participants in months, metab=name of the neurochemical, c1=control variable 1, c2=control variable 2, dv=dependent variable, conn=connectivity, X=independent variable, W=moderator, M=mediator, Y=dependent variable). The column “Task” can take three values, and they refer to 1=Attention Network Task, 2=Digit Comparison Task, or 3=Mental Rotation Task. If a table does not contain a “Task” column, it is because the underlying variables do not correspond to any of the three tasks exclusively. For example, in Figure 5C, Figure 7 and Supplementary file 7, the Visuomotor Processing score was the (z-scored) aggregate of Tasks 1-3, and in Figure 5A-B and Figure 6, none of the variables are specific to any of the three tasks. The column “Assessment” can take two values, and they refer to 1=First assessment and 2=Second assessment. Moreover, a subset of the MRI data used in this study has been previously deposited in XNAT (<https://central.xnat.org/data/projects/PN21>).

**Figures 2-4**

| **File Name** | **Task** | **Assessment** | **metab** | **age** | **c1** | **c2** | **dv** |
| --- | --- | --- | --- | --- | --- | --- | --- |
| F2A | 1 | 1 | IPS Glutamate | Chronological age | Mean drift rate | Boundary separation | Visuomotor Processing |
| F2B | 1 | 1 | IPS GABA | Chronological age | Mean drift rate | Boundary separation | Visuomotor Processing |
| F2C | 1 | 2 | IPS Glutamate | Chronological age | Mean drift rate | Boundary separation | Visuomotor Processing |
| F2D | 1 | 2 | IPS GABA | Chronological age | Mean drift rate | Boundary separation | Visuomotor Processing |
| F3A | 2 | 1 | IPS Glutamate | Chronological age | Mean drift rate | Boundary separation | Visuomotor Processing |
| F3B | 2 | 1 | IPS GABA | Chronological age | Mean drift rate | Boundary separation | Visuomotor Processing |
| F3C | 2 | 2 | IPS Glutamate | Chronological age | Mean drift rate | Boundary separation | Visuomotor Processing |
| F3D | 2 | 2 | IPS GABA | Chronological age | Mean drift rate | Boundary separation | Visuomotor Processing |
| F4A | 3 | 1 | IPS Glutamate | Chronological age | Mean drift rate | Boundary separation | Visuomotor Processing |
| F4B | 3 | 1 | IPS GABA | Chronological age | Mean drift rate | Boundary separation | Visuomotor Processing |
| F4C | 3 | 2 | IPS Glutamate | Chronological age | Mean drift rate | Boundary separation | Visuomotor Processing |
| F4D | 3 | 2 | IPS GABA | Chronological age | Mean drift rate | Boundary separation | Visuomotor Processing |

**Figure 5A-B**

| **File Name** | **Assessment** | **metab** | **age** | **dv** |
| --- | --- | --- | --- | --- |
| F5A | 1 | IPS Glutamate | Chronological age | Visuomotor Connectivity |
| F5B | 1 | IPS GABA | Chronological age | Visuomotor Connectivity |

**Figure 5C**

| **File Name** | **Assessment** | **conn** | **age** | **dv** |
| --- | --- | --- | --- | --- |
| F5C | 1 | Visuomotor Connectivity | Chronological age | Visuomotor Processing |

**Figure 6**

| **File Name** | **Assessment** | **metab** | **age** | **dv** |
| --- | --- | --- | --- | --- |
| F6A | 1 | IPS Glutamate | Chronological age | Intelligence |
| F6B | 1 | IPS GABA | Chronological age | Intelligence |
| F6C | 2 | IPS Glutamate | Chronological age | Intelligence |
| F6D | 2 | IPS GABA | Chronological age | Intelligence |

**Figure 7**

| **File Name** | **Assessment** | **X** | **W** | **M** | **dv** |
| --- | --- | --- | --- | --- | --- |
| F7A | 1 | IPS GABA | Chronological age | Visuomotor Processing | Intelligence |
| F7B | 1 | IPS Glutamate | Chronological age | Visuomotor Processing | Intelligence |

**Supplementary file 7**

| **File Name** | **Assessment** | **X** | **W** | **M** | **dv** |
| --- | --- | --- | --- | --- | --- |
| SF71 | 1 | IPS GABA | Chronological age | Visuomotor Connectivity | Visuomotor Processing |
| SF72 | 1 | IPS Glutamate | Chronological age | Visuomotor Connectivity | Visuomotor Processing |
